# Mathematical Framework for the Representation of the Travel of an Accelerometer-Based Texture Testing Device

**DOI:** 10.3390/s25113273

**Published:** 2025-05-22

**Authors:** Harald Paulsen, Christian Peham, Johannes Peter Schramel, Margit Gföhler

**Affiliations:** 1 Research Unit for Biomechanics and Rehabilitation Engineering, TU Wien, 1060 Vienna, Austria; margit.gfoehler@tuwien.ac.at; 2 Movement Science Group, University Equine Hospital, University of Veterinary Medicine, 1210 Vienna, Austria; christian.peham@vetmeduni.ac.at (C.P.); johannes.schramel@vetmeduni.ac.at (J.P.S.)

**Keywords:** free fall model, hammer head model, spring constant, energy recovery, Young’s modulus, resonance frequency

## Abstract

Recently, an accelerometer-based device (Vienna Surface Tester (VST)) has been developed for testing the surface characteristics of floors, beddings and turf grounds. The accelerometers are placed in a sphere, which will be dropped in free fall on a test surface. By observing changes in acceleration during impact, researchers can deduce various material characteristics. A down-sized version of this device (Surface Tester of Food Resilience (STFR)) has been proposed for texture testing of foods. Whereas the movement of the VST can be described by the laws of free fall, the STFR follows a constrained circular path due to its attachment to a rod and swivel. We refined the mathematical representation of the different phases of the STFR spherical probe’s trajectory (fall, impact and rebound), and we modified the mathematical models for the STFR probe to extend the measurement range.

## 1. Introduction

A recently developed device [1] for assessing the mechanical properties of large animal bedding and turf surfaces has been documented in the scientific literature. Studies have examined the characteristics of this device in testing livestock bedding [2] and turf grounds [3], while its relation to other turf surface testers has been reviewed [4]. The Vienna Surface Tester (VST) consists of a spherical probe, approximately the size of a standard bowling ball, equipped with two accelerometers [1]. The device is released in free fall onto the test surface, where it indents and rebounds before undergoing successive impacts [1,2]. Acceleration data from the device are analyzed using computational models to determine material properties. A modified, smaller version of the VST, the Surface Tester of Food Resilience (STFR), was developed for testing food texture. Unlike the VST, the STFR features a smaller spherical probe that follows a rotational trajectory due to its attachment to a rod and swivel.

Preliminary modifications to the release mechanism [5] have been conducted, along with studies correlating STFR data with Warner–Bratzler shear force [6] and texture profile analysis [7].

**Figure 1:**
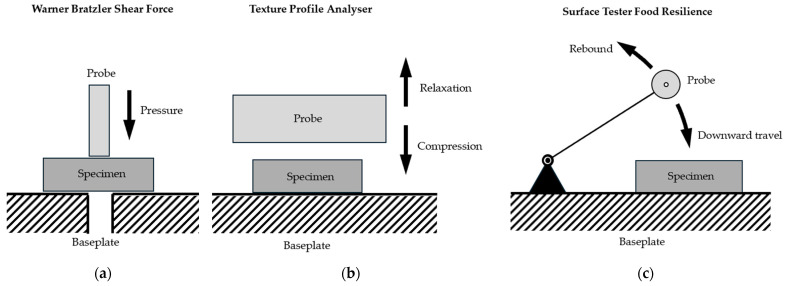
Principles of operation: Established methods for material characteristics of meats rely on a probe which travels vertically at constant speed against the specimen. Either the specimen is dissected (Warner–Bratzler shear force, (**a**)) or the probe performs compression/relaxation cycles (texture profile analysis, (**b**)). In both cases time–force diagrams are generated from which material characteristics can be calculated. In contrast, the Surface Tester Food Resilience (**c**) records changes in speed and these changes are used to calculate material characteristics.

The principles of operation of these methods are shown in Figure 1.

A key research question is whether refinements should be made to both the mechanical design and the mathematical models used for material characterization. The trajectory of the STFR probe follows a circular path, resembling the motion of a hammerhead, which differs from the free fall of the VST. Since the STFR uses the same formulas for the calculations as the VST, the question is whether the concept of free fall is appropriate for the STFR.

Therefore, the goal of this article is to highlight the differences in the results between the simplified free fall to an improved rotational trajectory model, in particular for the initial distance of the sphere to the specimen surface. We modified the mathematical models for the STFR probe to widen the measurement range (i.e., the elevation angle), with the goal of implementing the model in the STFR software, V. 1.0. Our approach seeks to develop a precise mathematical model capturing the different phases of the spherical probe’s motion.

## 2. Materials, Methods and Results

Components and the mode of operation of the STFR are described in Section 2.1, whereas the mathematical and physical foundations are elaborated in Section 2.2. Surface characterization parameters derived from the set of formulae in Section 2.2 are presented in Section 2.3. Comprehensive mathematical derivations, including modifications for STFR, are presented in Document S2.

### 2.1. The STFR and Its Mode of Operation

#### 2.1.1. The STFR Device

**Figure 2:**
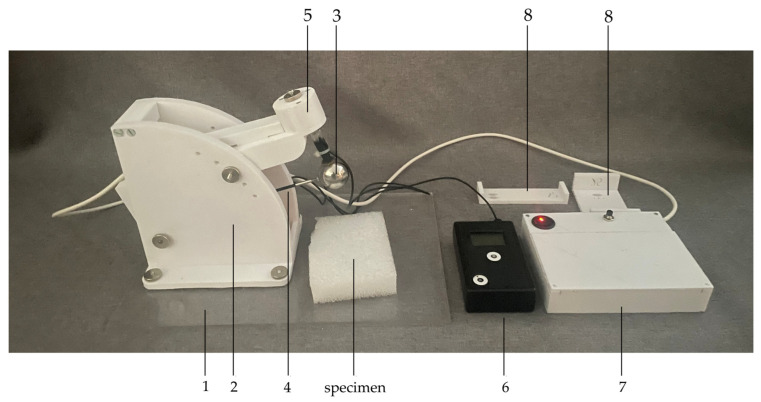
Main components of the “Surface Tester of Food Resilience” (STFR). 1 = baseplate; 2 = rig with a height-adjustable electromagnet; 3 = sphere containing two accelerometers; 4 = carbon-fiber rod connecting the sphere to a swivel; 5 = magnetic holding device attached to the sphere; 6 = digital data acquisition system; 7 = trigger for the electromagnetic holder and power supply; 8 = gauges (25 mm, 50 mm, 75 mm) to check the distance between specimen surface and sphere.

The components of the Surface Tester of Food Resilience (STFR) are shown in Figure 2. In detail, the STFR consists of the following:

- A baseplate (“1” in Figure 2);
- A rig attached to the baseplate, with a height-adjustable electromagnet, 2;
- A sphere 3, made from steel, with 30 mm diameter and a mass of 0.104 kg; inside the sphere, two accelerometers (one sensitive; ±2× *g*; one less sensitive; up to 200× *g*) are placed and the sphere is attached to a carbon-fiber rod 4 with a swivel on the other end; the distance from the center of the sphere to the rotation center of the swivel is 170 mm;
- A magnetic holding device 5 attached to the sphere;
- A digital data acquisition system which records and stores acceleration data for further analysis 6;
- A trigger for the electromagnetic holder and power supply 7;
- Spacers (25 mm, 50 mm, 75 mm) to check the distance between specimen surface and sphere.

The device is designed for specimens with a thickness of 25 mm. The rig allows pre-adjustment of the height of the sphere (25, 50 and 75 mm), and precise adjustment by a spring-loaded set screw. The specimen was a foam board of rectangular shape with a base area of 100 mm × 100 mm and a thickness of 25 mm.

#### 2.1.2. Principle of Operation

The surface test device consists of an impactor in the shape of a sphere, equipped with two accelerometers to perform a measurement. It is released from a predetermined height based on experimental requirements. The first accelerometer (A) is a sensitive type (±2× *g*) to detect small changes in acceleration with a good signal-to-noise ratio. Accelerometer B measures the peak acceleration of the impact. Both are mounted close together. When the impactor is stationary, the accelerometers register a gravitational acceleration of 9.81 m∙s^−2^. When the mass is freely moving then the reading is immediately 0× *g*.

**Figure 3:**
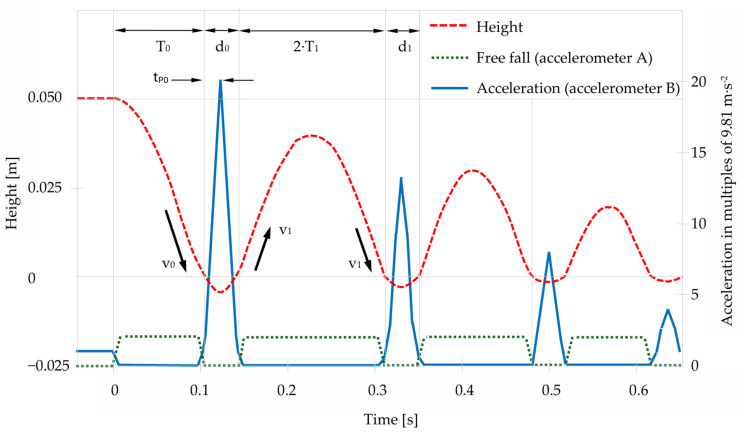
Schematic representation of first few collisions. After *T*_0_, the mass hits the ground and remains in contact during *d*_0_. Maximum acceleration is achieved after *t_P_*_0_ when *v* = 0. Zero gravity is present during free falling and between bounces. Mass, surface, drop height (energy) and material properties determine the evoked peak acceleration. Bouncing height and peak acceleration are reduced by every impact as energy is dissipated.

Accelerometer A detects free fall and subsequent upward motion after impact. Figure 3 depicts distance and acceleration over time of a mass dropping from a dedicated height on a compressible surface and bouncing multiple times.

#### 2.1.3. Data Recorded by the STFR

Data measured by the STFR are recorded in *.csv format on an SD card. Measurements were performed by the STFR and their units are given in Table 1.

In addition, the STFR recording unit performs calculations according to Table 2 and stores the results on the SD card. The formulae given in Table 2 apply to small falling heights [1] (p. 4).

**Figure 4:**
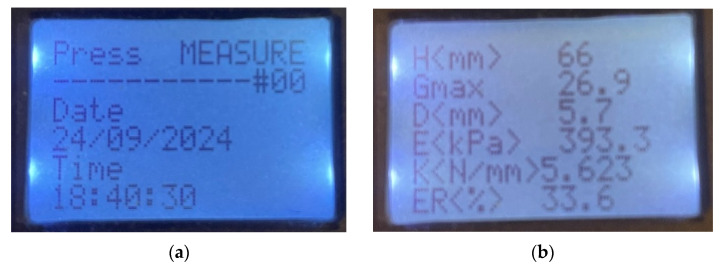
Display readings of the STFR (**a**) before measurement and (**b**) results displayed after measurement.

The display of the STFR conveniently displays the results calculated according to Table 2; see Figure 4.

### 2.2. Mathematical–Physical Modeling of the Movement of the Sphere

Based on the physics of the movement, we elaborate a mathematical model in the form of a differential equation in Section 2.2.1, whereas Section 2.2.2 presents a way to solve said equation for small angles. Section 2.2.3 employs the power series method.

**Figure 5:**
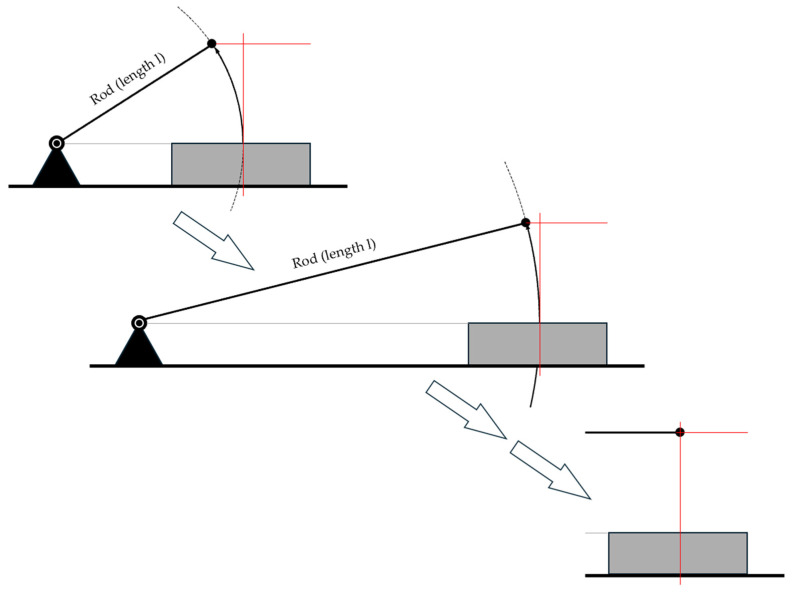
The effect of elongation of the guiding rod on the travel path of the sphere.

Although VST and STFR use the same principle (i.e., measurement of time and of changes in acceleration), they differ in mechanical layout. Thus, we study the similarities and differences by using mathematical–physical models. The starting point is the design of the STFR, since the circular motion of the STFR sphere will approach a free fall, when the length of the rod is increased (Figure 5).

**Figure 6:**
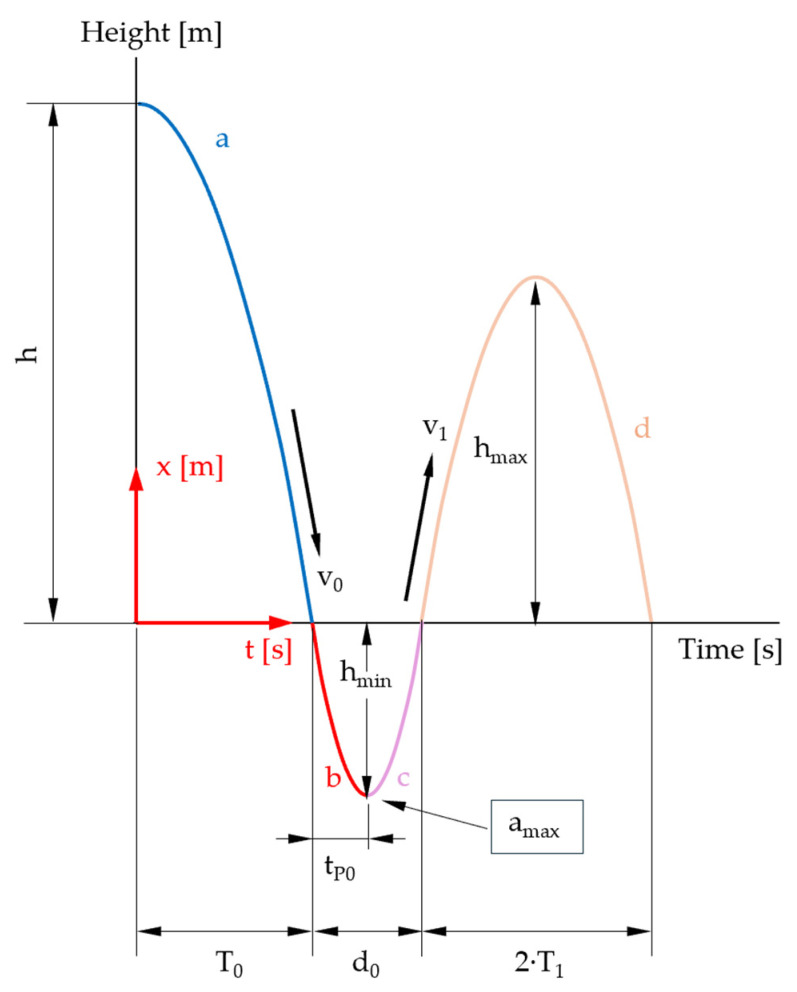
Representative phases of the travel of the sphere: a = free fall in air; b = indentation of the specimen; c = rise in the sphere to the initial level of the specimen surface; d = rise in the sphere in air (illustration not to scale).

In order to adapt the formulae given in Table 2 for larger falling heights, we study the movement of the sphere in air and then in an ideal, viscoelastic specimen. To this end, the travel of the sphere is separated in four different phases (see Figure 6).

#### 2.2.1. Derivation of the Equation of Motion

For modeling, the sphere is represented by its center of mass, and only its vertical travel is considered. The center of mass and the center of gravity of the sphere are different due to the mass of the rod. We consider the approximation through the center of the sphere, as the mass of the rod (carbon fiber; 3 mm diameter, 155 mm length; density approx. 2 g/cm^3^) with *m_rod_* = 0.15^2^∙15.5∙π∙2 g = 2.2 g is very low compared to the mass of the sphere of 104 g.

**Figure 7:**
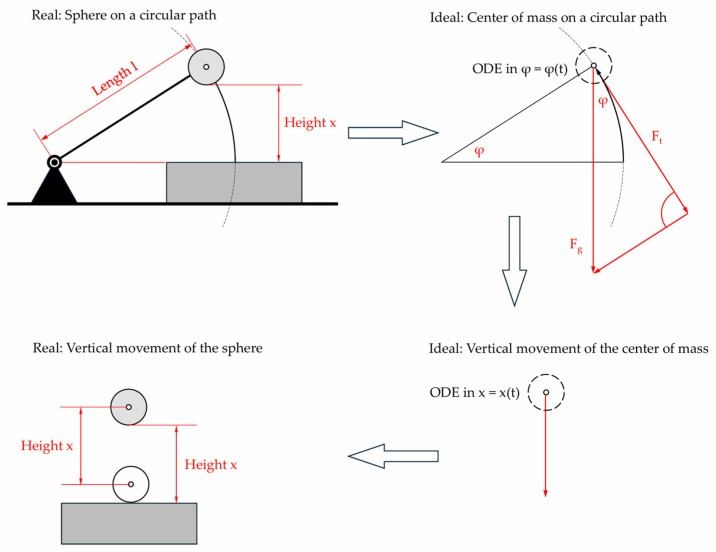
Stages of modeling. ODE = ordinary differential equation.

The results from this simplified model are then applied on the real sphere (Figure 7).

**Figure 8:**
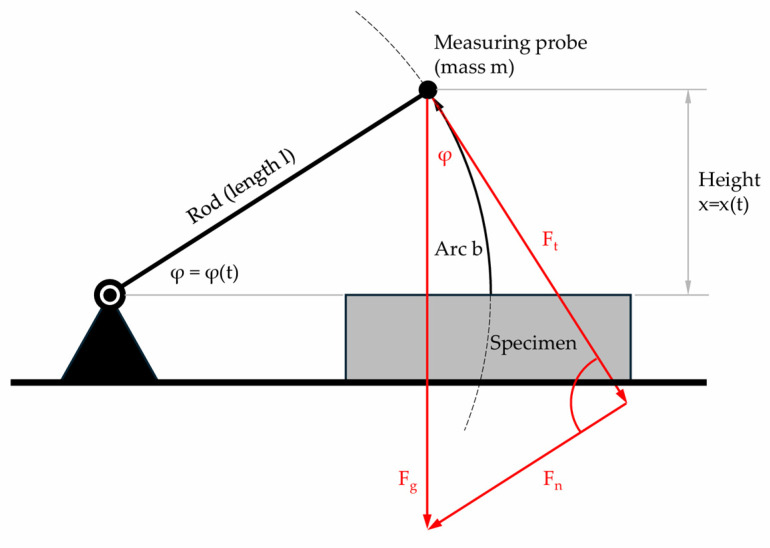
Representation of the parameters.

The schemes to the right of Figure 7 represent the mathematical–physical approach. The parameters used are shown in Figure 8.

The movement of the center of mass in the air is described by the following, Equation (1), with parameters as explained in Figure 8.

(1) $$F_{t}=F_{g}\cdot \cos\left(\varphi \right)\;\;\;with\;\;\;\;0^{rad}\leq \varphi <{\frac{\pi }{2}}^{rad}$$

The buoyancy force [8] (pp. 340, 351) can be neglected because $F_{buoyancy}=m\cdot g\cdot \frac{\rho_{air}}{\rho_{iron}}\approx 0.104\;kg\cdot 9.81\;m\cdot s^{-2}\cdot \frac{1}{7960}=1.282\cdot 10^{-4}\;N\approx 0\;N$. Due to the low speed of the ball, which reaches a maximum of around 1.124 m∙s^−1^ in free fall from a height of 0.075 m, the air friction [8] can also be neglected ($F_{Stokes}=6\cdot \pi \cdot r\cdot \eta_{air}\cdot v_{max}\approx 6\cdot \pi \cdot 0.015\cdot 1.8\cdot 10^{-5}\cdot 1.124\;N=5.720\cdot 10^{-6}\;N\approx 0\;N$).

With

(2) $$\begin{array}{c} b\left(t\right)=l\cdot \varphi \left(t\right)\\ F_{t}=m\cdot \ddot{b}=m\cdot l\cdot \ddot{\varphi }\\ F_{g}=m\cdot g \end{array}$$

we obtain the non-linear differential equation for the angle of rotation φ(t) as

(3) $$l\cdot \ddot{\varphi }=-g\cdot cos\left(\varphi \right)$$

To determine the vertical distance *x* = *x*(*t*), we use the substitutions

(4) $$\begin{array}{c} x=l\cdot sin\left(\varphi \right)\\ \dot{x}=l\cdot cos\left(\varphi \right)\cdot \dot{\varphi }\\ \ddot{x}=l\cdot cos\left(\varphi \right)\cdot \ddot{\varphi }-l\cdot sin\left(\varphi \right)\cdot {\left(\dot{\varphi }\right)}^{2} \end{array}$$

and obtain the differential equation for the height *x(t)* as

(5) $$m\cdot \ddot{x}+m\cdot \frac{x\cdot {\left(\dot{x}\right)}^{2}}{l^{2}-x^{2}}-\frac{g\cdot m}{l^{2}}\cdot x^{2}=-m\cdot g\;$$

with 0≤x<l.

In our model, only the vertical part of the movement is considered. The derivation of the differential equation of the horizontal distances can be found in Document S2.

The behavior of the measuring probe in the specimen depends on its material properties. For the mathematical modeling of the movement in the specimen, it is assumed that the specimen behaves viscoelastic near the surface (Kelvin body) and that this behavior can therefore be described by a parallel connection of a spring element (with spring constant *k*) with a damping element (with damping constant *c*).

Therefore, in addition to the forces shown in Figure 7, a spring force $F_{k}=k\cdot x$ and a frictional force according to Stokes ([9]; low speeds) with $F_{c}=c\cdot \dot{x}$ are also considered. When these forces are considered in the differential equation for the height *x*(*t*), Equation (5) is extended to Equation (6):

(6) $$m\cdot \ddot{x}+m\cdot \frac{x\cdot {\left(\dot{x}\right)}^{2}}{l^{2}-x^{2}}-\frac{g\cdot m}{l^{2}}\cdot x^{2}=-m\cdot g-k\cdot x-c\cdot \dot{x}\;$$

with −l<x≤0.

At the same time, Equation (5) is obtained from Equation (6) for the movement in the air (*c* = *k* = 0). We therefore refer to Equation (6) as the universal equation.

#### 2.2.2. Solving the Equation of Motion with Small-Angle Approximation

In this section, the equation of motion is solved using the usual physics linearization of the differential Equation (3) using a small-angle approximation [10]. The motion in the air and the motion in the specimen are analyzed separately.

**Figure 9:**
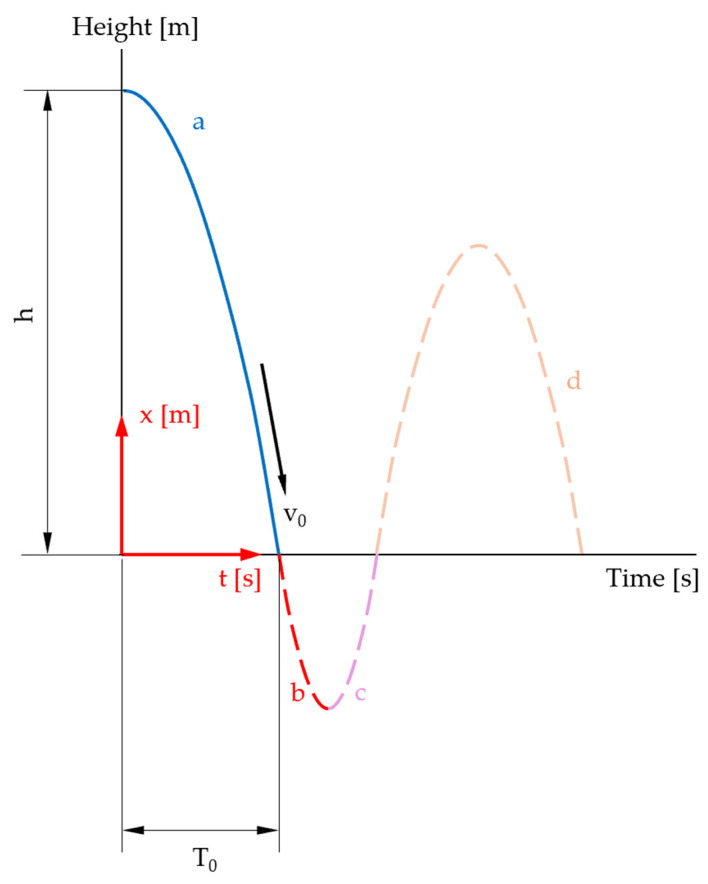
Time–distance diagram: first movement phase highlighted (solid blue curve) (illustration not to scale).

The first movement phase in the air is shown in Figure 9 and covers the period from triggering to the impact of the sphere on the specimen.

Basically, this is a rotary movement of the measuring probe around an axis and not a free fall.

When applying the small-angle approximation (7),

(7) $$\begin{array}{c} cos\left(\varphi \right)\approx 1\\ x=l\cdot sin\left(\varphi \right)\approx l\cdot \varphi \end{array}$$

to the differential Equation (3), a simplified differential Equation (8) is obtained that describes the free fall.

(8) $$l\cdot \ddot{\varphi }=-g=\ddot{x}$$

For the first movement phase (initial fall phase with initial height *h*), Equation (9) results as follows, assuming the initial conditions $x\left(0\right)=h,\;\dot{x}\left(0\right)=0$.

(9) $$x=-\frac{g}{2}\cdot t^{2}+h$$

As can be seen in Table 3 and in File S3, this approximation is acceptable for small deflections (angles) only.

The small-angle approximation allows to establish a rule for dimensioning the ratio of the rod length *l* to the maximum drop height *h*. If a limit is specified for the deviation in % (*pd*) then the approximation

(10) x=l·sin(φ)≈l·φ

results in the condition

(11) h:l≤φ:1

for the maximum initial height *h* = *x*.

As an example, we consider a rod length *l* = 170 mm and a maximum deviation of 1%. This results in an initial height *h* of around 24 mm, since *h* = 0.17 m∙0.1408 = 0.023936 m = 23.936 mm.

The ratio *l*:*h* = 10:1 given in the patent specification [1] (p. 4) corresponds to a maximum angle of 0.1 rad ≈ 5.7° with an error of around 0.5%.

**Figure 10:**
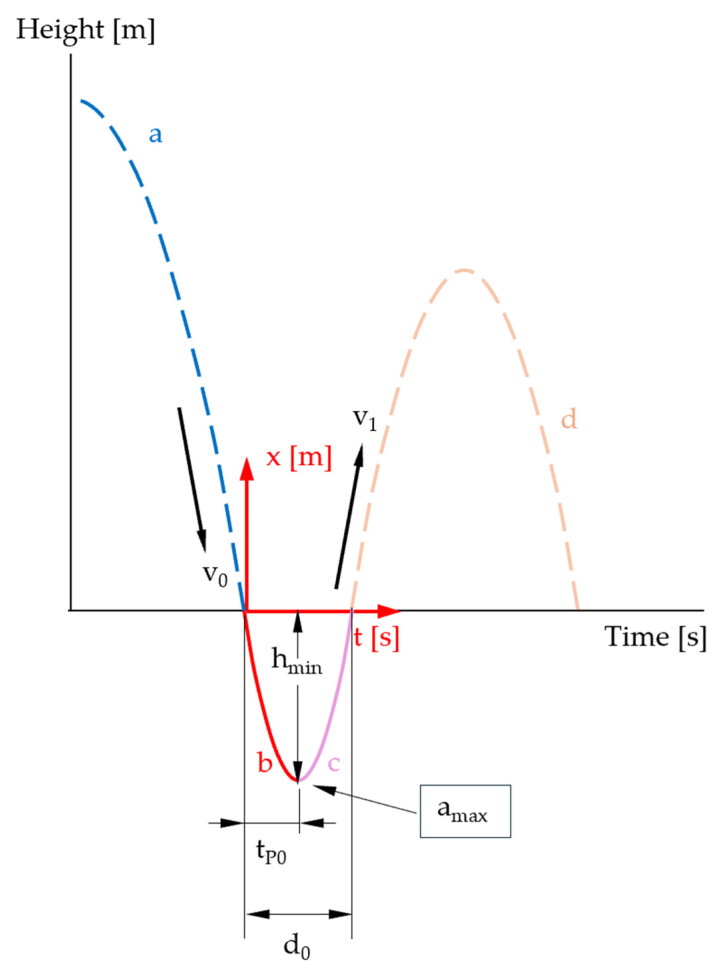
Time–distance diagram: second and third movement phases (summarized) highlighted (illustration not to scale).

The movement of the measuring body in the specimen is shown in Figure 10 (solid red and pink curve).

In contrast to the derivation for the movement in the air, the differential equation defining the movement in the specimen is not simplified using small-angle approximation at first, and then the forces acting through the specimen are considered in the calculation. Instead, the differential equation of the circular path (5) is converted into a differential equation of a free fall in the specimen, assuming that the length of the rod *l* approaches infinity (*l*→∞).

The following Equation (12) results from (6) because of *l*→∞.

(12) $$m\cdot \ddot{x}=-m\cdot g-k\cdot x-c\cdot \dot{x}\;$$

Further transformations lead to the second-order linear differential Equation (13).

(13) $$\ddot{x}+\frac{c}{m}\cdot \dot{x}+\frac{k}{m}\cdot x=-g,\;\;\;-l<x\leq 0$$

Considering $c^{2}<4\cdot m\cdot k$ (damped oscillation; [11] pp. 518–519) and using a local coordinate system with $x\left(0\right)=0,\;\dot{x}\left(0\right)=v_{0}$ gives the following solution (14).

(14) $$\begin{array}{c} x=\frac{e^{-\delta \cdot t}}{{\omega_{0}}^{2}}\cdot \left(g\cdot \cos\left(\omega \cdot t\right)+\frac{v_{0}\cdot {\omega_{0}}^{2}+\delta \cdot g}{\omega }\cdot \sin\left(\omega \cdot t\right)\right)-\frac{g}{{\omega_{0}}^{2}}\;\\ \delta =\frac{c}{2\cdot m},\;{\;\omega }_{0}=\sqrt{\frac{k}{m}},\;\omega =\sqrt{{\omega_{0}}^{2}-{\;\delta }^{2}} \end{array}\;\dot{x}=e^{-\delta \cdot t}\cdot \left(v_{0}\cdot \cos\left(\omega \cdot t\right)-\frac{v_{0}\cdot \delta +g}{\omega }\cdot \sin\left(\omega \cdot t\right)\right)$$

Numerical approximation methods are required to calculate the time of impact on the specimen from this transcendental function. As we wanted to create the simplest type of implementation in the STFR software, we did not consider this approach any further.

It is also possible to replace this function with its MacLaurin series. The calculation of the MacLaurin series corresponding to Equation (14) can be found in Document S2. It can be reported that the 3rd degree MacLaurin series corresponds to the 3rd degree polynomial function that results from the limit transition *l*→∞ from Equation (28).

#### 2.2.3. Solving the Equations of Motion Using the Power Series Approach

The small-angle solution described in Section 2.2.2 allows elegant approximate solutions but has the disadvantage that the accuracy is determined by the approximation before the differential equation is solved. In contrast, solving by means of a power series approach allows the desired accuracy to be adjusted by changing the degree of the power series after the differential equation has been solved.

The universal differential Equation (6) is first generally solved using a power series approach [12], (15).

(15) $$x=x\left(t\right)=a_{0}+a_{1}\cdot t^{1}+a_{2}\cdot t^{2}{+a}_{3}\cdot t^{3}+a_{4}\cdot t^{4}\;{+a}_{3}\cdot t^{3}+a_{4}\cdot t^{4}+\ldots +a_{n}\cdot t^{n}$$

The coefficients *a*_0_, *a*_1_, …, *a_n_* are calculated using Matlab^®^ R2023a (File S4) and the output terms are further simplified.

- For the individual movement phases, we consider the initial and boundary conditions. For the first flight phase (Figure 6: curve section a), we obtain Equation (16).

(16) $$x\left(t\right)=h-\left(1-\frac{h^{2}}{l^{2}}\right)\cdot \frac{g\cdot t^{2}}{2}-\frac{2\cdot h}{3\cdot l^{2}}\cdot \left(1-\frac{h^{2}}{l^{2}}\right)\cdot \frac{{g^{2}\cdot t}^{4}}{4}+\cdots$$

using the initial conditions of $x\left(0\right)=h,\dot{\;x}\left(0\right)=0,\;k=0,\;c=0$.

- The first movement phase in the specimen (Figure 6: curve sections b and c) is represented by Equation (17).

(17) $$\begin{array}{c} x\left(t\right)=v_{0}\cdot t-\frac{g\cdot m+c\cdot v_{0}}{2\cdot m}\cdot t^{2}+\frac{c^{2}\cdot l^{2}\cdot v_{0}+g\cdot c\cdot l^{2}\cdot m-k\cdot l^{2}\cdot m\cdot v_{0}-m^{2}\cdot {v_{0}}^{3}}{6\cdot l^{2}\cdot m^{2}}\cdot t^{3}\\ +\frac{-c^{3}\cdot l^{2}\cdot v_{0}-{g\cdot c}^{2}\cdot l^{2}\cdot m+2\cdot k\cdot c\cdot l^{2}\cdot m\cdot v_{0}+6\cdot c\cdot m^{2}\cdot {v_{0}}^{3}+g\cdot k\cdot l^{2}\cdot m^{2}+7\cdot g\cdot m^{3}\cdot {v_{0}}^{2}}{24\cdot l^{2}\cdot m^{3}}\cdot t^{4}+\cdots \end{array}$$

since $x\left(0\right)=0,\;\dot{x}\left(0\right)=v_{0}$ (in a local coordinate system).

- The second phase in the air (Figure 6: curve section d) is described by the function (18).

(18) $$x\left(t\right)=v_{1}\cdot t-\frac{g}{2}\cdot t^{2}-\frac{{v_{1}}^{3}}{6\cdot l^{2}}\cdot t^{3}+\frac{7\cdot g\cdot {v_{1}}^{2}}{24\cdot l^{2}}\cdot t^{4}+\cdots .$$

since $x\left(0\right)=0,\;\dot{\;x\;}\left(0\right)=v_{1}\;$(in a local coordinate system).

For practical application, the power series are approximated by their 2nd or 3rd degree polynomials, as these are easy to work with analytically. Convergence analyses are therefore not carried out. The quality of the approximations is analyzed by experimental means in a further article.

In the following chapters, the individual sections of the curves (Figure 6) are analyzed separately, and the estimates of the maximum error are also given.

#### 2.2.4. Initial Drop Movement in the Power Series Approach

The first drop phase (Figure 9) is of particular interest because its description is independent of specimen properties and should therefore provide the same results for all types of specimens.

The quadratic approximation of Equation (16), which we will refer to as the hammer model, is (19):

(19) $$\begin{array}{c} x\left(t\right)=h-\left(1-\frac{h^{2}}{l^{2}}\right)\cdot \frac{g\cdot t^{2}}{2}\\ \Delta x=\frac{g\cdot t^{2}\cdot h^{2}}{l^{3}}\cdot \Delta l+\left(1+\frac{g\cdot h\cdot t^{2}}{l^{2}}\right)\cdot \Delta h+g\cdot t\cdot \left(1-\frac{h^{2}}{l^{2}}\right)\cdot \Delta t \end{array}\;\begin{array}{c} v\left(t\right)=-\left(1-\frac{h^{2}}{l^{2}}\right)\cdot g\cdot t\\ \Delta v=\frac{2\cdot g\cdot h^{2}\cdot t}{l^{3}}\cdot \Delta l+\frac{2\cdot g\cdot h\cdot t}{l^{2}}\cdot \Delta h+g\cdot \left(1-\frac{h^{2}}{l^{2}}\right)\cdot \Delta t \end{array}$$

To determine the error bounds, the absolute maximum error Δ*x* is specified using the (positive) errors Δ*h*, Δ*l* and Δ*t*.

From the quadratic approximation (19), the equation for free fall (20) is obtained with *l*→∞ as already mentioned in Section 2.2.

(20) $$\begin{array}{c} x\left(t\right)=h-\frac{g\cdot t^{2}}{2}\\ \Delta x=\Delta h+g\cdot t\cdot \Delta t \end{array}\;\begin{array}{c} v\left(t\right)=-g\cdot t\\ \Delta v=g\cdot \Delta t \end{array}$$

**Figure 11:**
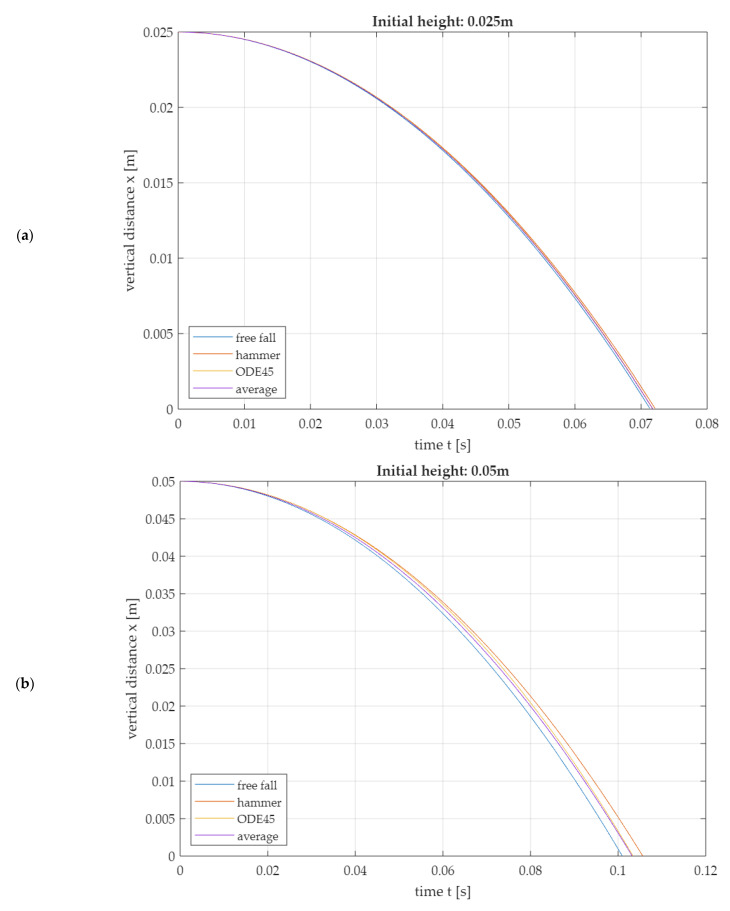

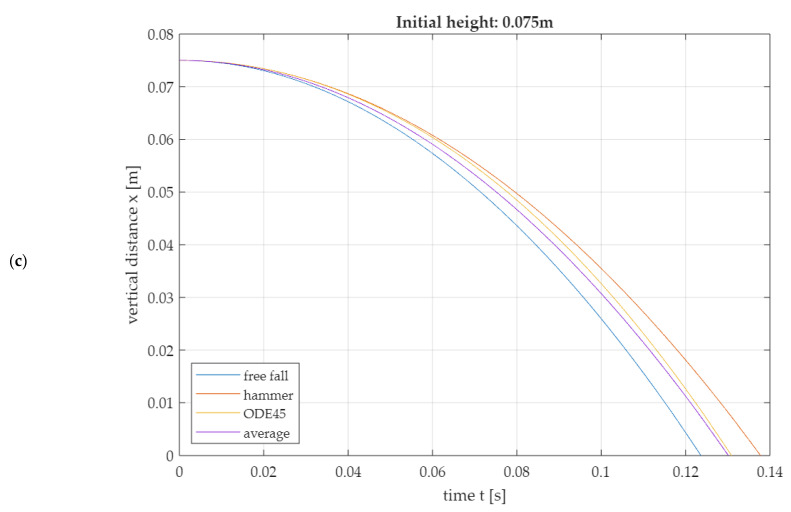
Models of the first fall phase in comparison: Numerical solution of the differential equation with ODE45 (Matlab^®^ R2023a), solution as free fall, solution in the 2nd-degree hammer model and solution in the average model, which results empirically. (**a**) Initial drop height 0.025 m, (**b**) initial drop height 0.050 m, (**c**) initial drop height 0.075 m.

For verification, the 2nd-degree polynomials (19) are compared with the trajectory of the free fall (20) and a numerical solution of the differential Equation (5) using Matlab^®^ R2023a, as seen in Figure 11. The numerical solution is determined using the ODE45 solver for ordinary differential equations (see File S5).

Since the solutions of the free fall model and the hammer model differ more and more with increasing initial height but lay on different sides of the numerically generated simulation solution with ODE45 (Figure 11), it is proposed to additionally consider an average model (21). This is created as the arithmetic mean of the function values of the free fall and the hammer model; see Equation (21).

(21) $$\begin{array}{c} x\left(t\right)=h-\left(1-\frac{h^{2}}{2\cdot l^{2}}\right)\cdot \frac{g\cdot t^{2}}{2}\\ \Delta x=\frac{g\cdot t^{2}}{2}\cdot \frac{h^{2}}{l^{3}}\cdot \Delta l+\left(1+\frac{g\cdot t^{2}}{2}\cdot \frac{h}{l^{2}}\right)\cdot \Delta h+g\cdot t\cdot \left(1-\frac{h^{2}}{2\cdot l^{2}}\right)\cdot \Delta t \end{array}\;\begin{array}{c} v\left(t\right)=-\left(1-\frac{h^{2}}{2\cdot l^{2}}\right)\cdot g\cdot t\\ \Delta v=\frac{g\cdot h^{2}\cdot t}{l^{3}}\cdot \Delta l+\frac{g\cdot h\cdot t}{l^{2}}\cdot \Delta h+g\cdot \left(1-\frac{h^{2}}{2\cdot l^{2}}\right)\cdot \Delta t \end{array}$$

This results in three different mathematical approximations for the initial drop movement. The decision as to which approximation should be used for implementation in the software is made by comparing the approximations of special curve parameters.

The first parameter is the initial drop height h, which is calculated by the STFR from the impact time *T*_0_. It is used as a validity criterion for the measurement process: If the calculated value *h* deviates significantly from the preset height, the measurement is considered invalid and discarded.

The recalculation of the height *h* with knowledge of *t = T*_0_ results in formula sets for the free fall model (22), the 2nd-degree hammer model (23) and the average model (24) due to *x* = 0.

(22) $$h=\frac{g\cdot {T_{0}}^{2}}{2}\;\frac{\Delta h}{h}=2\cdot \frac{\Delta T_{0}}{T_{0}}$$

(23) $$h=-\frac{l^{2}}{g\cdot {T_{0}}^{2}}+\frac{l}{g\cdot {T_{0}}^{2}}\cdot \sqrt{l^{2}+g^{2}\cdot {T_{0}}^{4}}\;\Delta h=\left|\frac{2\cdot l}{g\cdot {T_{0}}^{2}}-\frac{2\cdot l^{2}+g^{2}\cdot {T_{0}}^{4}}{g\cdot {T_{0}}^{2}}\cdot \frac{\sqrt{l^{2}+g^{2}\cdot {T_{0}}^{4}}}{l^{2}+g^{2}\cdot {T_{0}}^{4}}\right|\cdot \Delta l+\left|\frac{2\cdot l^{2}}{g\cdot {T_{0}}^{3}}-\frac{2\cdot l^{3}}{g\cdot {T_{0}}^{3}}\cdot \frac{\sqrt{l^{2}+g^{2}\cdot {T_{0}}^{4}}}{l^{2}+g^{2}\cdot {T_{0}}^{4}}\right|\cdot \Delta T_{0}$$

(24) $$h=-\frac{2\cdot l^{2}}{g\cdot {T_{0}}^{2}}+\frac{l}{g\cdot {T_{0}}^{2}}\cdot \sqrt{4\cdot l^{2}+2\cdot g^{2}\cdot {T_{0}}^{4}}\;\Delta h=\left|\frac{4\cdot l}{g\cdot {T_{0}}^{2}}-\frac{8\cdot l^{2}+2\cdot g^{2}\cdot {T_{0}}^{4}}{g\cdot {T_{0}}^{2}}\cdot \frac{\sqrt{4\cdot l^{2}+2\cdot g^{2}\cdot {T_{0}}^{4}}}{4\cdot l^{2}+2\cdot g^{2}\cdot {T_{0}}^{4}}\right|\cdot \Delta l+\left|\frac{4\cdot l^{2}}{g\cdot {T_{0}}^{3}}-\frac{8\cdot l^{3}}{g\cdot {T_{0}}^{3}}\cdot \frac{\sqrt{4\cdot l^{2}+2\cdot g^{2}\cdot {T_{0}}^{4}}}{4\cdot l^{2}+2\cdot g^{2}\cdot {T_{0}}^{4}}\right|\cdot \Delta T_{0}$$

The decision as to which of the three models should be used to calculate the initial height can only be made in comparison with experimentally obtained measurement data and is part of a following article.

The second and third parameters are the time *T*_0_ until the first impact on the specimen and the transition velocity *v*_0_ < 0 at the first contact. The formulae for the free fall model (25), the 2nd-degree hammer model (26) and the average model (27) are as follows:

(25) $$\begin{array}{c} T_{0}=\sqrt{\frac{2\cdot h}{g}}\\ \frac{\Delta T_{0}}{T_{0}}=\frac{1}{2}\cdot \frac{\Delta h}{h} \end{array}\;v_{0}=-g\cdot T_{0}\;\frac{\Delta v_{0}}{\left|v_{0}\right|}=\frac{\Delta T_{0}}{T_{0}}$$

(26) $$\begin{array}{c} T_{0}=\sqrt{\frac{l^{2}}{l^{2}-h^{2}}}\cdot \sqrt{\frac{2\cdot h}{g}}\\ \frac{\Delta T_{0}}{T_{0}}=\frac{h^{2}}{l^{2}-h^{2}}\cdot \frac{\Delta l}{l}+\frac{1}{2}\cdot \frac{l^{2}+h^{2}}{l^{2}-h^{2}}\cdot \frac{\Delta h}{h} \end{array}\;v_{0}=-\left(1-\frac{h^{2}}{l^{2}}\right)\cdot g\cdot T_{0}\;\frac{\Delta v_{0}}{\left|v_{0}\right|}=\frac{2\cdot h^{2}}{l^{2}-h^{2}}\cdot \left(\frac{\Delta h}{h}+\frac{\Delta l}{l}\right)+\frac{\Delta T_{0}}{T_{0}}$$

(27) $$\begin{array}{c} T_{0}=\sqrt{\frac{{2\cdot l}^{2}}{{2\cdot l}^{2}-h^{2}}}\cdot \sqrt{\frac{2\cdot h}{g}}\\ \frac{\Delta T_{0}}{T_{0}}=\frac{h^{2}}{{2\cdot l}^{2}-h^{2}}\cdot \frac{\Delta l}{l}+\frac{1}{2}\cdot \frac{{2\cdot l}^{2}+h^{2}}{{2\cdot l}^{2}-h^{2}}\cdot \frac{\Delta h}{h} \end{array}\;v_{0}=-\left(1-\frac{h^{2}}{2\cdot l^{2}}\right)\cdot g\cdot T_{0}\;\frac{\Delta v_{0}}{\left|v_{0}\right|}=\frac{2\cdot h^{2}}{2\cdot l^{2}-h^{2}}\cdot \left(\frac{\Delta h}{h}+\frac{\Delta l}{l}\right)+\frac{\Delta T_{0}}{T_{0}}$$

Formulas (25)–(27) allow a comparison of the analytically determined approximation solutions with the numerically determined solution (see File S5) of the differential Equation (5) in Table 4. The results suggest that the impact times are best described by the average model and the impact velocities are best described by the free fall model.

#### 2.2.5. Movement in the Specimen in the Power Series Approach

This movement phase begins when the measuring probe first comes into contact with the specimen and ends when it exits the specimen.

Specimens usually have a multi-layered and inhomogeneous structure, thus, a simple and at the same time exact mathematical description taking into account the material properties is hardly possible. To obtain (approximated) characteristic values, several different methods are subsequently applied:

In the currently implemented software of the STFR, the acceleration function is assumed to be a triangular function without consideration of the specimen structure and material properties. An approximation for the maximum penetration depth is determined from this approach [1] (p. 3). A new approach is to assume the specimen (at least near the surface) as a viscoelastic solid. Then, the solution of the descriptive differential Equation (6) using a power series approach (17) results in the cubic approximation (28).

(28) $$x\left(t\right)=v_{0}\cdot t-\frac{g\cdot m+c\cdot v_{0}}{2\cdot m}\cdot t^{2}+\frac{c^{2}\cdot l^{2}\cdot v_{0}+g\cdot c\cdot l^{2}\cdot m-k\cdot l^{2}\cdot m\cdot v_{0}-m^{2}\cdot {v_{0}}^{3}}{6\cdot l^{2}\cdot m^{2}}\cdot t^{3}$$

in which both the spring constant *k* and the damping constant *c* occur.

**Figure 12:**
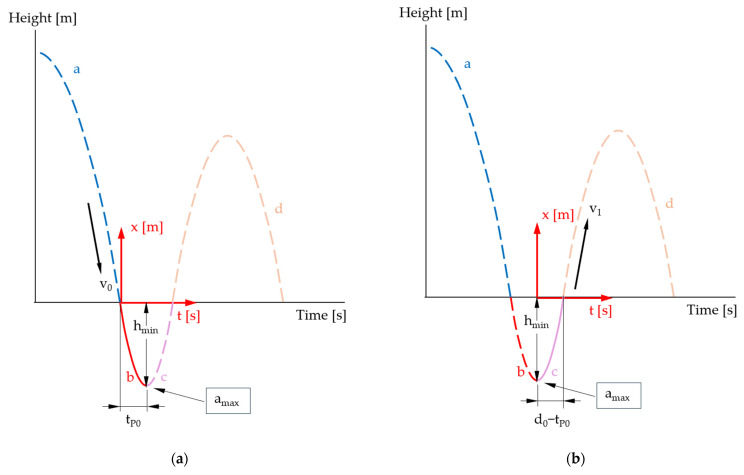
Descending curve section (**a**); ascending curve section (**b**) (illustration not to scale).

- To be able to compare the model described above with measured values of the STFR, further modeling is carried out using cubic functions. To take possible asymmetry into account, the movement in the specimen is divided into a descending curve section (Figure 12a) and an ascending curve section (Figure 12b). At the transition point, both curves coincide in speed and acceleration.

For the descending part *p* (Figure 11a), using the boundary conditions $\begin{array}{cc} p\left(0\right)=0, & \dot{p}\left(0\right)=v_{0}<0 \end{array},\;\begin{array}{cc} \dot{p}\left(t_{P0}\right)=0, & \ddot{p}\left(t_{P0}\right)=a_{max}>0 \end{array}$, we obtain the function (29).

(29) $$p\left(t\right)=v_{0}\cdot t-\frac{a_{max}\cdot t_{P0}+2\cdot v_{0}}{2\cdot t_{P0}}\cdot t^{2}+\frac{a_{max}\cdot t_{P0}+v_{0}}{3\cdot {t_{P0}}^{2}}\cdot t^{3}$$

This model describes the curve from the entry point to the lowest point in the specimen, whereby the measured value *d*_0_ (dwell time in the specimen) is not used.

The penetration depth (height at the extreme point *t_P_*_0_) in the cubic model, considering $a_{max}=G_{max}\cdot g$, is then

(30) $$h_{min}=p\left(t_{P0}\right)=\frac{t_{P0}}{6}\cdot \left(2\cdot v_{0}-G_{max}\cdot g\cdot t_{P0}\right)=D\;\Delta h_{min}=\Delta p=\frac{\left|v_{0}-G_{max}\cdot g\cdot t_{P0}\right|}{3}\cdot \Delta t_{P0}+\frac{t_{P0}}{3}\cdot \Delta v_{0}+\frac{{t_{P0}}^{2}}{6}\cdot g\cdot \Delta G_{max}$$

For the ascent in the specimen (Figure 12b), another cubic function q is modeled using $\begin{array}{cc} q\left(0\right)=h_{min}<0, & \dot{q}\left(0\right)=0, \end{array}\;\begin{array}{cc} q\left(d_{0}-t_{P0}\right)=0, & \ddot{q}\left(0\right)=a_{max} \end{array}$ with a new local origin and definition range [0; *d*_0_ − *t_P_*_0_] as

(31) $$q\left(t\right)=-\frac{a_{max}\cdot {\left(d_{0}-t_{P0}\right)}^{2}+2\cdot h_{min}}{2\cdot {\left(d_{0}-t_{P0}\right)}^{3}}\cdot t^{3}+\frac{a_{max}}{2}\cdot t^{2}+h_{min}$$

The velocity at the exit point is then calculated as follows because $a_{max}=G_{max}\cdot g$

(32) $$v_{1}=\dot{q}\left(d_{0}-t_{P0}\right)=-\frac{G_{max}\cdot g\cdot {\left(d_{0}-t_{P0}\right)}^{2}+6\cdot h_{min}}{2\cdot \left(d_{0}-t_{P0}\right)}=-\frac{G_{max}\cdot g\cdot \left(d_{0}-t_{P0}\right)}{2}-\frac{3\cdot h_{min}}{d_{0}-t_{P0}}\;\Delta v_{1}=\frac{d_{0}-t_{P0}}{2}\cdot g\cdot \Delta G_{max}+\frac{3}{d_{0}-t_{P0}}\cdot \Delta h_{min}+\frac{1}{2}\cdot \left|\frac{G_{max}\cdot g\cdot {\left(d_{0}-t_{P0}\right)}^{2}-6\cdot h_{min}}{{\left(d_{0}-t_{P0}\right)}^{2}}\right|\cdot \left(\Delta d_{0}+\Delta t_{P0}\right)$$

#### 2.2.6. Flight Phase After the First Rebound in the Power Series Approach

**Figure 13:**
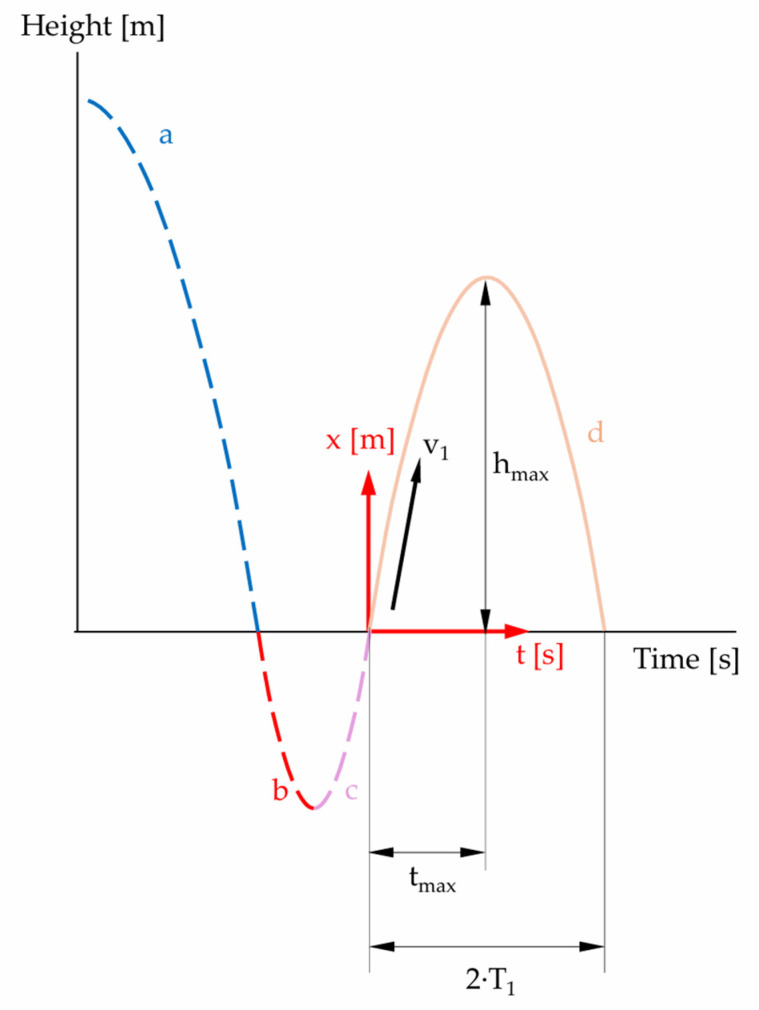
Path of the sphere after leaving the specimen (illustration not to scale).

After the measurement probe exits the specimen (Figure 13), there is a movement in the air, which is described mathematically using the results from Section 2.2.3, Formula (18), whereby the transition parameter used is the transition velocity *v*_1_.

The quadratic approximation is

(33) $$x\left(t\right)=-\frac{g}{2}\cdot t^{2}+v_{1}\cdot t\;\;\;\;\;\;\;v_{1}>0$$

and describes the movement of a vertical throw. This approximation makes sense, as several impact, penetration and rebound cycles take place in succession and the rebound height decreases with each cycle, bringing the model of circular motion closer and closer to the model of free fall. In this quadratic approximation the highest point is given by (34).

(34) $$t_{max}=\frac{v_{1}}{g}\;\;\;\;\;\;\;\;\frac{\Delta t_{max}}{t_{max}}=\frac{\Delta v_{1}}{v_{1}}\;h_{max}=\frac{1}{2}\cdot \frac{{v_{1}}^{2}}{g}\;\;\;\;\frac{\Delta h_{max}}{h_{max}}=2\cdot \frac{\Delta v_{1}}{v_{1}}$$

To determine a quality criterion for quadratic approximation, the trajectory is additionally approximated by a 2nd-degree polynomial function, for which the following representation results due to $\begin{array}{ccc} x\left(0\right)=0, & \dot{x}\left(0\right)=v_{1}, & x\left(2\cdot T_{1}\right)=0,\; \end{array}\;0\leq t\leq T_{1}$ as

(35) $$x\left(t\right)=-\frac{v_{1}}{2\cdot T_{1}}\cdot t^{2}+v_{1}\cdot t\;\;\;\;\;\;\;\;\;\Delta x=v_{1}\cdot \left(1-\frac{t}{T_{1}}\right)\cdot \Delta t+t\cdot \left(1-\frac{t}{2\cdot T_{1}}\right)\cdot \Delta v_{1}+\frac{v_{1}\cdot t^{2}}{2\cdot {T_{1}}^{2}}\cdot \Delta T_{1}$$

The highest point of the trajectory is at $\left(T_{1}|\frac{v_{1}\cdot T_{1}}{2}\right)$. From the description of its heights in the two models (33) and (34), after equating $h_{max}=\frac{1}{2}\cdot \frac{{v_{1}}^{2}}{g}=\frac{v_{1}\cdot T_{1}}{2}$, the condition (36) follows.

(36) $$\frac{v_{1}}{T_{1}}=g\;\;\;\;\frac{\Delta T_{1}}{T_{1}}=\frac{\Delta v_{1}}{v_{1}}$$

This relationship is important because it correlates the value *T*_1_ obtained by measurement with the value *v*_1_ determined by calculation.

### 2.3. Derivation of Formulae for Material Characteristics

Various material parameters can be derived from the previous mathematical formulae. We propose a new approximation for the values of energy restitution and spring constant in the STFR. In addition, an approximation for the damping constant is derived.

#### 2.3.1. Approximations for Energy Restitution

The energy restitution provides information about the reflection behavior of the specimen. The formula for energy restitution presented in Document S1 (p. 2) is based on the free fall model.

Alternatively, the new Formula (37) for the energy restitution is calculated from (34) as

(37) $$E_{r}=\frac{h_{max}}{h}=\frac{{v_{1}}^{2}}{2\cdot g\cdot h}\;\;\;\;\frac{\Delta E_{r}}{E_{r}}=\frac{\Delta h}{h}+2\cdot \frac{\Delta v_{1}}{v_{1}}$$

#### 2.3.2. Approximations for Spring Constant and Damping Constant

The STFR calculates an estimate of the spring constant near to the specimen surface [1] (p. 3), Document S1 (p. 2). We develop additional approximations, one for the calculation of the spring constant for larger distances from the surface and two approximations for the calculation of the damping constant.

- The second order approximation (38)

(38) $$x\left(t\right)=v_{0}\cdot t-\frac{g\cdot m+c\cdot v_{0}}{2\cdot m}\cdot t^{2}$$

of (28) allows us to estimate the damping constant *c_s_* near to the specimen surface. Given $\ddot{x}\left(t_{P0}\right)=a_{max}=G_{max}\cdot g$, *c_s_* can be approximated in Equation (39).

(39) $$c_{s}=-\frac{G_{max}+1}{v_{0}}\cdot g\cdot m\;\Delta c_{s}=g\cdot \left(\left|\frac{m}{v_{0}}\right|\cdot \Delta G_{max}+\left|\frac{G_{max}+1}{{v_{0}}^{2}}\right|\cdot m\cdot \Delta v_{0}+\left\lceil \frac{G_{max}+1}{v_{0}}\right\rceil \cdot \Delta m\right)$$

- Considering the third-order Equations (28) and (29), comparison of the coefficients results in Equation (40),

(40) $$\begin{array}{c} \frac{g\cdot m+c\cdot v_{0}}{2\cdot m}=\frac{a_{max}\cdot t_{P0}+2\cdot v_{0}}{2\cdot t_{P0}}\\ \frac{c^{2}\cdot l^{2}\cdot v_{0}+g\cdot c\cdot l^{2}\cdot m-k\cdot l^{2}\cdot m\cdot v_{0}-m^{2}\cdot {v_{0}}^{3}}{6\cdot l^{2}\cdot m^{2}}=\frac{a_{max}\cdot t_{P0}+v_{0}}{3\cdot {t_{P0}}^{2}} \end{array}$$

and for *c* and *k* with $a_{max}=G_{max}\cdot g$, approximations (41) for spring constant *k_h_* and damping constant *c_h_*, for larger distances from the surface

(41) $$c_{h}=\left(\frac{G_{max}-1}{v_{0}}\cdot g+\frac{2}{t_{P0}}\right)\cdot m\;\Delta c_{h}=\left|\frac{G_{max}-1}{v_{0}}\cdot g+\frac{2}{t_{P0}}\right|\cdot \Delta m+\frac{m}{\left|v_{0}\right|}\cdot g\cdot \Delta G_{max}+\frac{2\cdot m}{{t_{P0}}^{2}}\cdot \Delta t_{P0}+\frac{m\cdot \left(G_{max}-1\right)}{{v_{0}}^{2}}\cdot g\cdot \Delta v_{0}$$

and

(42) $$k_{h}=\left(\frac{G_{max}\cdot g^{2}\cdot \left(G_{max}-1\right)}{{v_{0}}^{2}}+\frac{2\cdot g\cdot \left(G_{max}-1\right)}{t_{P0}\cdot v_{0}}+\frac{2}{{t_{P0}}^{2}}-\frac{{v_{0}}^{2}}{l^{2}}\right)\cdot m\;\begin{array}{c} \Delta k_{h}=\left|\frac{G_{max}\cdot g^{2}\cdot \left(G_{max}-1\right)}{{v_{0}}^{2}}+\frac{2\cdot g\cdot \left(G_{max}-1\right)}{t_{P0}\cdot v_{0}}+\frac{2}{{t_{P0}}^{2}}-\frac{{v_{0}}^{2}}{l^{2}}\right|\cdot \Delta m+m\cdot \left|\frac{2\cdot G_{max}-1}{{v_{0}}^{2}}\cdot g+\frac{2}{t_{P0}\cdot v_{0}}\right|\cdot g\cdot \Delta G_{max}\\ +\frac{2\cdot m}{{t_{P0}}^{2}}\cdot \left|\frac{G_{max}-1}{v_{0}}\cdot g+\frac{2}{t_{P0}}\right|\cdot \Delta t_{P0}+2\cdot m\cdot \left|\frac{G_{max}\cdot g^{2}\cdot \left(G_{max}-1\right)}{{v_{0}}^{3}}+\frac{G_{max}-1}{t_{P0}\cdot {v_{0}}^{2}}\cdot g+\frac{v_{0}}{l^{2}}\right|\cdot \Delta v_{0}\\ \;\;\;\;\;+\frac{2\cdot {v_{0}}^{2}\cdot m}{l^{3}}\cdot \Delta l \end{array}$$

It should be noted that these approximation formulas do not take into account the shape of the probe, which plays a role in the penetration behavior in the specimen.

### 2.4. Practical Relevance of the Selection of Mathematical Models for Calculation of T_0_

In addition to the formulae discussed in the previous sections, approximate values for the spring constant *K* (43), the energy restitution *E_R_* (44), the resonance frequency *f_n_* (45) and Young’s modulus E* (46) as seen in Table 2 are calculated in the STFR. As the value *T*_0_ is not included in the calculation of the resonance frequency *f_n_* and the Young’s modulus E*, these are not considered further.

The spring constant *K* and its relative error Δ*K*/*K* are calculated according to Formula (43).

(43) $$K=\frac{{G_{max}}^{2}\cdot m}{{T_{0}}^{2}}\;\frac{\Delta K}{K}=2\cdot \frac{\Delta G_{max}}{G_{max}}+2\cdot \frac{\Delta T_{0}}{T_{0}}+\frac{\Delta m}{m}$$

The energy restitution *E_R_* and its relative error Δ*E_R_/E_R_* are calculated according to Formula (44).

(44) $$E_{R}={\left(\frac{T_{1}}{T_{0}}\right)}^{2}\;\frac{\Delta E_{R}}{E_{R}}=2\cdot \left(\frac{{\Delta T}_{0}}{T_{0}}+\frac{\Delta T_{1}}{T_{1}}\right)$$

The resonance frequency *f_n_* and its relative error Δ*f_n_*/*f_n_* are calculated according to Formula (45).

(45) $$f_{n}=\frac{1}{2\cdot d_{0}}\;\frac{\Delta f_{n}}{f_{n}}=\frac{\Delta d_{0}}{d_{0}}$$

The Young’s modulus E* and its relative error ΔE*/E* are calculated according to Formula (46).

(46) $$E*=\frac{3}{4}\cdot \frac{m\cdot g\cdot G_{max}}{\sqrt{R}\cdot \sqrt{{\left|D\right|}^{3}}}\;\frac{\Delta E*}{E*}=\frac{\Delta m}{m}+\frac{\Delta G_{max}}{G_{max}}+\frac{1}{2}\cdot \frac{\Delta R}{R}+\frac{3}{2}\cdot \frac{\Delta D}{\left|D\right|}$$

Since the STFR uses *T*_0_ to calculate the spring constant as well as energy recovery, the correct estimation of *T*_0_ is crucial. With reference to Equations (25)–(27), the relations of *T*_0_ calculated by the three models is given in Equation (47).

(47) $$T_{0,free}:T_{0,hammer}:T_{0,average}=1:\sqrt{\frac{l^{2}}{l^{2}-h^{2}}}:\sqrt{\frac{2\cdot l^{2}}{{2\cdot l}^{2}-h^{2}}}$$

Table 5 illustrates the differences in *T*_0_ for different falling heights and models. Differences up to 4.6 and 11.5 percent occurred at falling heights of 50 and 75 mm, respectively. This is not unexpected, when referring to Table 3. Additional data to Table 5 are available in File S6. More detailed analysis will be given in a follow-up article.

The formulae for the spring constant and the energy restitution do not only depend on *T*_0_ but also on *G_max_* and *T*_1_. Since the material properties are not known, no simple mathematical relationship can be given to select the appropriate model.

However, assuming that small changes in *T*_0_ have no effect on *G_max_* or *T*_1_, the Formulas (48) and (49) are obtained. It is noticeable that both energy restitution and Young’s modulus behave in the same way.

(48) $$E_{R,free}:E_{R,hammer}:E_{R,average}=1:\frac{l^{2}-h^{2}}{l^{2}}:\frac{2\cdot l^{2}-h^{2}}{{2\cdot l}^{2}}$$

(49) $$k_{free}:k_{hammer}:k_{average}=1:\frac{l^{2}-h^{2}}{l^{2}}:\frac{2\cdot l^{2}-h^{2}}{{2\cdot l}^{2}}$$

Such differences in *T*_0_ have a greater effect on the calculation of the spring constant and energy restitution, as *T*_0_ is always squared in the calculation as seen in Table 6.

## 3. Discussion

### 3.1. Rationale for This Study

The STFR has been developed from a surface testing device, VST. The latter device is an impact tester, like, e.g., the Clegg hammer [13], in which the probe approaches the specimen surface in free fall, i.e., it accelerates. These devices have proven useful in standardized testing of soil, beddings and turf [4], are easy to use and require less trained staff. With respect to testing beddings for large animals and turf, it can be argued that impact testers do not consider the soil-to-hoof/claw interactions and the structure of the hoof or claw apparatus [4].

The STFR was developed as a scaled down version of the VST to allow testing of textural properties of meat and meat products, taking into account that conventional texture testing methods such as shearing, simulating the action of the front teeth (Warner–Bratzler shear force) [14,15], compression tests or “two-bite-tests”, simulating mastication (Texture Profile Analysis, TPA) [16], also consider only basic characteristics of the biting or chewing process. Although the significance of the parameters reported in TPA tests has been questioned [17], TPA remains a standard in food testing.

On a first view, the STFR has some similarities with the TPA. The latter operates, however, at constant speed. Conversely, the sphere is accelerated towards and then reflected from the specimen. Preliminary studies [7] reported high and significant correlations for spring constant and elasticity modulus (assessed by STFR) to work-to-hardness-point and total work (compression test) for selected meat products. The experimental setup in our study included falling heights of 50 mm and 100 mm, which raised the question of whether the free fall formulae used in the STFR software would calculate correct results for a sphere following a circular trajectory.

### 3.2. Selection of Mathematical Approaches and Constraints

To extend the range (i.e., falling height) of the sphere, a mathematical–physical model was needed, which considers both the circular path of the sphere and a free fall model (vertical travel only). In addition, the specimen was modeled as a viscoelastic solid. Frictional forces occurring in the swivel joint were not considered. The mathematical description was achieved using a differential equation. A similar approach has been followed in a recently published paper on fluid dynamics in tubes [18].

Commonly, small-angle approximations are employed to transform non-linear differential equations into linear differential equations with constant coefficients. This simplifies the mathematical apparatus and allows us to use already well-known solutions or damping oscillations. However, when approximations are used in the very initial phase of the mathematical analysis, it is not possible to refine the accuracy of results, if desired.

We used power series calculations to solve the equations describing the movement of the sphere and to elaborate second- or third-order approximations. In parallel, data retrieved from the STFR were used to form second-order approximations. Both approaches allowed us to derive formulae with less complex algebraical structure, which can be implemented in the STFR software. A focus was put on estimations of error, to allow a better comparison of results generated by different models.

We refrained from using polynomial equations of higher degrees (i.e., higher than third degree), with a view on the ease of implementation, e.g., by spreadsheet calculation software. Otherwise, numerical analysis procedures need to be employed, requiring more effort in programming the STFR software.

### 3.3. Consequences of the Modeling Approaches for the Refinement of the STFR Software

The current version of STFR uses the set of calculations designed for the “large-scale”, free fall VST. Since the apparatus measures time and (changes in) acceleration, formulae are needed to calculate distances and forces. The current STFR software uses the free fall model as an approximation, i.e., only the vertical travel is considered. This generates deviations from the true circular path, which combines both vertical travel and changes in the relative horizontal position. The magnitude of deviation depends on the elevation angle of the STFR. Thus, the correctness of results generated by the STFR is sensitive to the elevation angle (corresponding to the initial distance of sphere to specimen). Consequently, the patent description of the apparatus specifies a maximum elevation of 5.7°.

For the first phase of the travel of the sphere, we compared the empirical model averaged from the hammer and free fall model to results generated by a simulation and found that this averaged model performs superior to the free fall model. For elevations up to 5.7°, the free fall model is an approximation with sufficient accuracy, whereas the average model performs superior from 5.7 to ca. 26.2° elevation, corresponding to falling heights from 17 mm to 75 mm (given a sphere diameter of 30 mm and a swivel-sphere center distance of 170 mm).

Further validations are planned by comparing models with STFR measurements and high-speed image analyses.

## 4. Final Conclusions

We presented a model describing a circular trajectory of an accelerometer-equipped sphere. The device is designed to test surface characteristics of viscoelastic solids. The device requires that the sphere rebounds and the current software do not handle situations where the sphere does not rebound. Admittedly, the speed of deceleration and the depth of indentation could be used to characterize resilience in gels and soft polymers.

The mathematical model aims at a correct representation of the trajectory of the sphere without introducing excessive complexity in the software. Since the friction forces in the swivel have not been taken into account, minimizing this forces is important in prototype design.

Ultimately, all efforts shall result in an easy to use, portable, economic device for non-destructive testing of surface characteristics.

The new mathematical model should yield more accurate results at high elevation angles than the free fall model. It can be expected that, at higher angles, there will be less variation in repeated tests, whereas at low angles, some variation might occur due to the resolution of the measuring device and inertia effects. Thus, the new mathematical model indirectly improves repeatability of measurements.

## Figures and Tables

**Table 1:** Measurements carried out by the STFR and the units as reported by the STFR.

Variable	Meaning	Physical Unit
*T*_0_	Duration from release of the sphere to the first impact (=first free-flight/-fall phase)	[s]
*d*_0_	Duration of the first contact phase of the sphere with the specimen surface	[ms]
*t_P_*_0_	Duration from first sphere-specimen contact to the extremum of acceleration	[ms]
2∙*T*_1_	Duration of the second free-flight phase	[s]
*G_max_*	Factor for calculating the peak acceleration	[g-unit]

The value *G_max_* is a factor with which the acceleration *a_max_ = G_max_∙g* can be calculated. For example, a measured value of *G_max_* = 10 means a peak acceleration of *a_max_* = 10∙9.81 m∙s^−2^ = 98.1 m∙s^−2^.

**Table 2:** Calculations performed by the STFR, their meaning and physical units of the results as reported by the STFR; see also Document S1.

Variable	Meaning	Physical Unit
$h=\frac{g}{2}\cdot {T_{0}}^{2}$	Initial height (calculated as the distance from the lowest point of the sphere to the specimen surface)	[mm]
$v_{0}=g\cdot T_{0}$	Velocity at first impact of the sphere	[m/s]
$D=g\cdot t_{P0}\cdot (T_{0}-\frac{G_{max}\cdot t_{P0}}{6})$	Maximum penetration depth of the specimen at impact	[mm]
$E*=\frac{3}{4}\cdot \frac{m\cdot g\cdot G_{max}}{\sqrt{R}\cdot \sqrt{{\left|D\right|}^{3}}}$	Young’s modulus	[kPa]
$K=\frac{{G_{max}}^{2}\cdot m}{{T_{0}}^{2}}$	Spring constant	[N/mm]
$E_{R}={\left(\frac{T_{1}}{T_{0}}\right)}^{2}$	Energy restitution	%
$f_{n}=\frac{1}{2\cdot d_{0}}$	Resonance frequency	[Hz]

**Table 3:** Deviation of small-angle approximation from *cos*(*φ*) in %, as a function of the angle *φ*.

*φ* [Radiant]	*φ* [Degrees]	*cos*(*φ*)	Approx. *cos*(*φ*)→1	$\mathrm{Deviation}\;pd=\frac{1-cos\left(\varphi \right)}{cos\left(\varphi \right)}$
0.1408	8.1	0.9901	1	1%
0.1984	11.4	0.9804	1	2%
0.3098	17.8	0.9524	1	5%
0.4296	24.6	0.9091	1	10%

**Table 4:** Comparison of the mathematical models with a simulation. *T*_0_ is the duration, *x* the height and *v_0_* the velocity when the probe hits the specimen. In the models, *T*_0_ is calculated exactly from the impact condition *x* = 0. As the simulation is analyzed point by point, the values in the simulation can only be determined approximately, which is particularly noticeable for the height *x* (≈0 mm).

Measuring Height [mm]	Subject	Free Fall Model	Hammer Model	Average Model	Simulation with ODE45
25 ± 1	Time *T*_0_ [ms]	71.392 ± 1.4278	72.177 ± 1.5167	71.781 ± 1.4716	71.808
	Height *x* [mm]	0 ± 0	0 ± 0	0 ± 0	0.00108
	Velocity *v*_0_ [m/s]	−0.70036 ± 0.014007	−0.69274 ± 0.015963	−0.69656 ± 0.014979	−0.70034
50 ± 1	Time *T*_0_ [ms]	100.96 ± 1.0096	105.64 ± 1.3153	103.22 ± 1.153	103.41
	Height *x* [mm]	0 ± 0	0 ± 0	0 ± 0	0.000379
	Velocity *v*_0_ [m/s]	−0.99045 ± 0.009905	−0.94665 ± 0.016427	−0.9688 ± 0.013089	−0.99045
75 ± 1	Time *T*_0_ [ms]	123.65 ± 0.82437	137.79 ± 1.5585	130.15 ± 1.1373	130.9
	Height *x* [mm]	0 ± 0	0 ± 0	0 ± 0	0.00143
	Velocity *v*_0_ [m/s]	−1.2131 ± 0.008087	−1.0886 ± 0.022424	−1.1525 ± 0.014847	−1.213

**Table 5:** Time to first impact *T*_0_ for different heights, calculated by the three different models and relative differences.

Initial Height [mm]	*T*_0,*free*_ [s]	*T*_0,*hammer*_ [s]	*T*_0,*average*_ [s]	*T*_0,*free*_:*T*_0,*hammer*_:*T*_0,*average*_ *
25	0.071393156	0.072176881	0.071781302	1:1.011:1.005
50	0.100963755	0.105636123	0.103220712	1:1.046:1.022
75	0.123654842	0.137789208	0.130149893	1:1.114:1.053

* displays the relative differences to *T*_0,*free*_. E.g., 1.011 means that *T*_0,*hammer*_ is 1.1% greater than *T*_0,*free*_.

**Table 6:** Energy restitution *E_R_* and spring constant *k* for different heights, calculated by the three different models.

Initial Height [mm]	$E_{R,free}:E_{R,hammer}:E_{R,average}$ * $k_{free}:k_{hammer}:k_{average}$
25	1:0.978:0.989
50	1:0.913:0.957
75	1:0.805:0.903

* expressed as the relative differences to *E*_*R*,*free*_. E.g., 0.978 means that *E*_*R*,*hammer*_ is 2.2% smaller than *E*_*R*,*free*_.

## Data Availability

Data are contained in the Supplementary Materials.

## Supplementary Materials

The following supporting information can be downloaded at https://www.mdpi.com/article/10.3390/s25113273/s1, Document S1: Methodology_ revised 2025.docx; Document S2: Derivations_of_the_formulas.pdf; File S3: deviation_cosine.xlsx; File S4: compute_powerseries_solving_ode.m; File S5: comparison_models.m; File S6: comparison_T0.xlsx.

## Abbreviations and Symbols

The following abbreviations are used in this manuscript:

|*x*|	Absolute amount (modulus) of x
. over letter	First derivation
.. over letter	Second derivation
Δ	Difference, error
2∙*T*_1_	Duration of the second free-flight phase, [s]
*a_max_*	Maximum acceleration, [m∙s^−2^]
*c_s_*	Damping constant near the specimen’s surface, [kg∙s^−1^]
*c_h_*	Damping constant for larger distances from the specimen’s surface, [kg∙s^−1^]
*D*	Maximum penetration depth of the specimen at impact, [mm]
*d*_0_	Duration of the first contact phase of the sphere with the specimen surface, [ms]
*E**	Young’s modulus, [kPa]
*E_R_*	Energy restitution, %
*E*_*R*,*average*_	Energy restitution calculated by the average model, %
*E*_*R*,*free*_	Energy restitution calculated by the free fall model, %
*E*_*R*,*hammer*_	Energy restitution calculated by the hammer model, %
*F*	Force, [N]
*f_n_*	Resonance frequency, [Hz]
*g*	g = 9.81 m∙s^−2^
*G_max_*	Factor for calculating the peak acceleration, [g-units]
*h*	Initial height, [mm]
*h_min_*	Depth of penetration of the sphere in the specimen, [mm]
*K, k*	Spring constant, [N∙mm^−1^]
*k_average_*	Spring constant calculated by the average model, [N∙mm^−1^]
*k_free_*	Spring constant calculated by the free fall model, [N∙mm^−1^]
*k_hammer_*	Spring constant calculated by the hammer model, [N∙mm^−1^]
*l*	Length of the rod, [mm]
ODE	Ordinary differential equations
*pd*	Deviation, %
rad	Radiant
*T*_0,*average*_	Time to first impact calculated by the average model, [ms]
*T*_0,*free*_	Time to first impact calculated by the free fall model, [ms]
*T*_0,*hammer*_	Time to first impact calculated by the hammer model, [ms]
*T*_0_	Duration from release of the sphere to first impact, [s]
*t_P_*_0_	Duration from first sphere-specimen contact phase to the extremum of acceleration, [ms]
*v*_0_	Velocity at first impact of the sphere, [m∙s^−1^]
*x*	Height, [mm]
*δ*	Damping coefficient, [s^−1^]
*η_air_*	Dynamic viscosity of air, [Pa∙s]
*ρ*	Density, [kg∙s^−2^]
*φ*	Angle, [rad]
*ω*	Angular frequency, [s^−1^]
*ω*_0_	Characteristic frequency, [s^−1^]
