# Supplementary material for: Mathematical Framework for the Representation of the Travel of an Accelerometer-Based Texture Testing Device
Source: Sensors (Basel). 2025 May 22;25(11):3273. doi: 10.3390/s25113273 (PMC12157044; doi:10.3390/s25113273)
Supplement: Supplementary file 1 [file sensors-25-03273-s001.zip › Derivations_of_the_formulas.pdf]

*Supplementary file*

# Derivations of the formulas

In this document, the derivations are sorted by chapter number. To make them easier to follow, all intermediate steps are generally listed. Formulas that originate from the article retain their numbering.

## Chapter 2.2.1

*Formula 2,3*

The force equation is derived in this section.

$$F_t = F_g \cdot \cos(\varphi) \quad with \quad 0^{rad} \leq \varphi < \frac{\pi^{rad}}{2} \tag{1}$$

$$\begin{aligned} b(t) &= l \cdot \varphi(t) \\ \dot{b}(t) &= l \cdot \dot{\varphi}(t) \\ \ddot{b}(t) &= l \cdot \ddot{\varphi}(t) \end{aligned}$$

$$\begin{aligned} b(t) &= l \cdot \varphi(t) \\ F_t &= m \cdot \ddot{b} = m \cdot l \cdot \ddot{\varphi} \\ F_g &= m \cdot g \end{aligned} \tag{2}$$

$$F_t = m \cdot l \cdot \ddot{\varphi} = -m \cdot g \cdot \cos(\varphi)$$

Result:

$$l \cdot \ddot{\varphi} = -g \cdot cos(\varphi) \tag{3}$$

Remark: The sign results from the direction of the force effect.

*Formula 6*

The differential equation is derived in this section.

$$\begin{aligned} x &= l \cdot sin(\varphi) \\ \dot{x} &= l \cdot cos(\varphi) \cdot \dot{\varphi} \\ \ddot{x} &= l \cdot cos(\varphi) \cdot \ddot{\varphi} - l \cdot sin(\varphi) \cdot (\dot{\varphi})^2 \end{aligned} \tag{4}$$

$$\begin{aligned} \ddot{x} &= l \cdot cos(\varphi) \cdot \ddot{\varphi} - l \cdot sin(\varphi) \cdot (\dot{\varphi})^2 \\ \ddot{x} &= -g \cdot cos^2(\varphi) - x \cdot (\dot{\varphi})^2 \end{aligned}$$

$$\ddot{x} = -g \cdot \left(1 - sin^2(\varphi)\right) - x \cdot (\dot{\varphi})^2$$

$$\ddot{x} = -g \cdot \left(1 - sin^2(\varphi)\right) - x \cdot \left(\frac{\dot{x}}{l \cdot cos(\varphi)}\right)^2$$

$$\ddot{x} = -g \cdot \left(1 - sin^2(\varphi)\right) - x \cdot \left(\frac{\dot{x}}{l \cdot \sqrt{1 - sin^2(\varphi)}}\right)^2$$

$$\ddot{x} = -g \cdot \left(1 - sin^2(\varphi)\right) - x \cdot \left(\frac{\dot{x}}{l \cdot \sqrt{1 - sin^2(\varphi)}}\right)^2$$

$$\ddot{x} = -g \cdot \left(1 - \left(\frac{x}{l}\right)^2\right) - x \cdot \left(\frac{\dot{x}}{l \cdot \sqrt{1 - \left(\frac{x}{l}\right)^2}}\right)^2$$

$$\ddot{x} = -g \cdot \left(1 - \left(\frac{x}{l}\right)^2\right) - x \cdot \frac{(\dot{x})^2}{l^2 \cdot \left(1 - \left(\frac{x}{l}\right)^2\right)}$$

$$\ddot{x} = -g \cdot \left(1 - \frac{x^2}{l^2}\right) - x \cdot \frac{(\dot{x})^2}{l^2 \cdot \left(1 - \frac{x^2}{l^2}\right)}$$

$$\ddot{x} = -g \cdot \left(1 - \frac{x^2}{l^2}\right) - x \cdot \frac{(\dot{x})^2}{l^2 \cdot \left(\frac{l^2 - x^2}{l^2}\right)}$$

$$\ddot{x} = -g \cdot \left(1 - \frac{x^2}{l^2}\right) - x \cdot \frac{(\dot{x})^2}{(l^2 - x^2)}$$

$$\ddot{x} + g \cdot \left(1 - \frac{x^2}{l^2}\right) + x \cdot \frac{(\dot{x})^2}{(l^2 - x^2)} = 0$$

$$\ddot{x} + \frac{x \cdot (\dot{x})^2}{l^2 - x^2} + g \cdot \left(1 - \frac{x^2}{l^2}\right) = 0$$

$$\ddot{x} + \frac{x \cdot (\dot{x})^2}{l^2 - x^2} + g - \frac{g \cdot x^2}{l^2} = 0$$

$$\ddot{x} + \frac{x \cdot (\dot{x})^2}{l^2 - x^2} - \frac{g}{l^2} \cdot x^2 = -g$$

finally after multiplication by m (in order to interpret the formula)

$$m \cdot \ddot{x} + m \cdot \frac{x \cdot (\dot{x})^2}{l^2 - x^2} - \frac{g \cdot m}{l^2} \cdot x^2 = -m \cdot g \tag{5}$$

$$m \cdot \ddot{x} + m \cdot \frac{x \cdot (\dot{x})^2}{l^2 - x^2} - \frac{g \cdot m}{l^2} \cdot x^2 = -m \cdot g - k \cdot x - c \cdot \dot{x} \tag{6}$$

## Chapter 2.2.2.

*Formula 9*

Solving the differential equation with the small angle approximation.

$$cos(\varphi) \approx 1$$
$$x = l \cdot sin(\varphi) \approx l \cdot \varphi \tag{7}$$

$$l \cdot \ddot{\varphi} = -g = \ddot{x} \tag{8}$$

$$-g = \ddot{x}$$

$$\dot{x} = \int \ddot{x}\, dx = -g \cdot x + C_1$$

$$x = \int \dot{x}\, dx = -\frac{g}{2} \cdot x^2 + C_1 \cdot x + C_2$$

The constraints are as follows

$$x(0) = h = initial\ height$$
$$\dot{x}(0) = 0 = initial\ speed$$

$$\dot{x}(0) = 0 = -g \cdot 0 + C_1 \ \rightarrow C_1 \ = 0$$

$$x(0) = h = -\frac{g}{2} \cdot 0^2 + C_1 \cdot 0 + C_2 \ \rightarrow C_2 = h$$

Result:

$$x = -\frac{g}{2} \cdot t^2 + h \tag{9}$$

*Formula 11*

Determination of an estimate for the maximum deflection.

$$x = l \cdot sin(\varphi) \approx l \cdot \varphi \tag{10}$$

$$x : l \approx \varphi : 1$$

Result:

$$h : l \leq \varphi : 1 \tag{11}$$

*Formula 14*

Transition from the circular path to vertical movement

Using (6) we get

$$\lim_{l\to\infty}\left(m\cdot\ddot{x}+m\cdot\frac{x\cdot(\dot{x})^2}{l^2-x^2}-\frac{g\cdot m}{l^2}\cdot x^2\right)=m\cdot\ddot{x}$$

$$m\cdot\ddot{x}=-m\cdot g-k\cdot x-c\cdot\dot{x} \tag{12}$$

$$\boldsymbol{m\cdot\ddot{x}+c\cdot\dot{x}+k\cdot x=-m\cdot g}$$

$$\boldsymbol{\ddot{x}+\frac{c}{m}\cdot\dot{x}+\frac{k}{m}\cdot x=-g}$$

Result:

$$\ddot{x}+\frac{c}{m}\cdot\dot{x}+\frac{k}{m}\cdot x\ =-g\ \ mit\ \ -l<x\leq 0 \tag{13}$$

Solving the homogeneous differential equation

$$\boldsymbol{\ddot{x}+\frac{c}{m}\cdot\dot{x}+\frac{k}{m}\cdot x\ =0}$$

Exponential approach

$$\boldsymbol{x=\widehat{C}\cdot e^{\lambda\cdot t}}$$
$$\boldsymbol{\dot{x}=\lambda\cdot\widehat{C}\cdot e^{\lambda\cdot t}}$$
$$\boldsymbol{\ddot{x}=\lambda^2\cdot\widehat{C}\cdot e^{\lambda\cdot t}}$$

Characteristic equation

$$\boldsymbol{\lambda^2\cdot\widehat{C}\cdot e^{\lambda\cdot t}+\frac{c}{m}\cdot\lambda^2\cdot\widehat{C}\cdot e^{\lambda\cdot t}+\frac{k}{m}\cdot\widehat{C}\cdot e^{\lambda\cdot t}\ =0}$$

$$\boldsymbol{\lambda^2+\frac{c}{m}\cdot\lambda^2+\frac{k}{m}\ =0,\ \ \widehat{C}\cdot e^{\lambda\cdot t}\neq 0}$$

Solutions for $\lambda$

$$\boldsymbol{\lambda_{1,2}=-\frac{c}{2\cdot m}\pm\sqrt{\left(\frac{c}{2\cdot m}\right)^2-\frac{k}{m}}}$$

with

$$\delta=\frac{c}{2\cdot m}\quad,\ \omega_0=\sqrt{\frac{k}{m}}>0,\ \omega_d=\sqrt{{\omega_0}^2-\delta^2}$$

and $c^2<4\cdot m\cdot k$ (damped oscillation)

$$\lambda_{1,2}=-\delta\pm\sqrt{\delta^2-{\omega_0}^2}=-\delta\pm i\cdot\omega_d,\quad i=\sqrt{-1}$$

$$x(t)=\widehat{C_1}\cdot e^{\lambda_1\cdot t}+\widehat{C_2}\cdot e^{\lambda_2\cdot t}$$

$$x(t) = \widehat{C_1} \cdot e^{(-\delta + i \cdot \omega_d) \cdot t} + \widehat{C_2} \cdot e^{(-\delta - i \cdot \omega_d) \cdot t}$$

$$x(t) = \widehat{C_1} \cdot e^{-\delta \cdot t} \cdot e^{i \cdot \omega_d \cdot t} + \widehat{C_2} \cdot e^{-\delta \cdot t} \cdot e^{-i \cdot \omega_d \cdot t}$$

$$x(t) = e^{-\delta \cdot t} \cdot \left(\widehat{C_1} \cdot e^{i \cdot \omega_d \cdot t} + \widehat{C_2} \cdot e^{-i \cdot \omega_d \cdot t}\right)$$

$$x(t) = e^{-\delta \cdot t} \cdot \left(\widehat{C_1} \cdot (\cos(\omega_d \cdot t) + i \cdot \sin(\omega_d \cdot t)) + \widehat{C_2} \cdot (\cos(\omega_d \cdot t) - i \cdot \sin(\omega_d \cdot t))\right)$$

$$x(t) = e^{-\delta \cdot t} \cdot \left(\widehat{C_1} \cdot \left(\cos(\omega_d \cdot t) + i \cdot \widehat{C_1} \cdot \sin(\omega_d \cdot t)\right) + \left(\widehat{C_2} \cdot \cos(\omega_d \cdot t) - i \cdot \widehat{C_2} \cdot \sin(\omega_d \cdot t)\right)\right)$$

$$x(t) = e^{-\delta \cdot t} \cdot \left(\widehat{C_1} \cdot \left(\cos(\omega_d \cdot t) + \widehat{C_2} \cdot \cos(\omega_d \cdot t)\right) + \left(i \cdot \widehat{C_1} \cdot \sin(\omega_d \cdot t) - i \cdot \widehat{C_2} \cdot \sin(\omega_d \cdot t)\right)\right)$$

$$x(t) = e^{-\delta \cdot t} \cdot \left(\left(\widehat{C_1} + \widehat{C_2}\right) \cdot \cos(\omega_d \cdot t) + i \cdot \left(\widehat{C_1} - \widehat{C_2}\right)\right) \cdot \sin(\omega_d \cdot t)\right)$$

With $C_1 = \widehat{C_1} + \widehat{C_2}$ and $C_2 = i \cdot \left(\widehat{C_1} - \widehat{C_2}\right)$ we get the solution of the homogenous differential equation as

$$(t) = e^{-\delta \cdot t} \cdot \left(C_1 \cdot cos(\omega_d \cdot t) + C_2 \cdot sin(\omega_d \cdot t)\right)$$

Approach for the particulate solution:

$$x_p(t) = A, \quad \dot{x}_p(t) = \ddot{x}_p(t) = 0$$

because of

$$s(t) = -g$$

This leads to

$$0 + \frac{c}{m} \cdot 0 + \frac{k}{m} \cdot A = -g$$

$$A = -\frac{g \cdot m}{k} = -\frac{g}{{\omega_0}^2}$$

General solution:

$$x(t) = e^{-\delta \cdot t} \cdot \left(C_1 \cdot cos(\omega_d \cdot t) + C_2 \cdot sin(\omega_d \cdot t)\right) - \frac{g}{{\omega_0}^2}$$

The boundary conditions are

$$x(0) = 0, \, \dot{x}(0) = v_0 < 0$$

We need

$$\dot{x}(t) = -\delta \cdot e^{-\delta \cdot t} \cdot \left(C_1 \cdot cos(\omega_d \cdot t) + C_2 \cdot sin(\omega_d \cdot t)\right) + e^{-\delta \cdot t} \cdot \left(-C_1 \cdot \omega_d \cdot sin(\omega_d \cdot t) + C_2 \cdot \omega_d \cdot cos(\omega_d \cdot t)\right)$$

$$\dot{x}(t) = e^{-\delta \cdot t} \cdot \left(-\delta \cdot C_1 \cdot cos(\omega_d \cdot t) - \delta \cdot C_2 \cdot sin(\omega_d \cdot t)\right) + e^{-\delta \cdot t} \cdot \left(-C_1 \cdot \omega_d \cdot sin(\omega_d \cdot t) + C_2 \cdot \omega_d \cdot cos(\omega_d \cdot t)\right)$$

$$\dot{x}(t) = e^{-\delta \cdot t} \cdot \left(-\delta \cdot C_1 \cdot cos(\omega_d \cdot t) - \delta \cdot C_2 \cdot sin(\omega_d \cdot t) - C_1 \cdot \omega_d \cdot sin(\omega_d \cdot t) + C_2 \cdot \omega_d \cdot cos(\omega_d \cdot t)\right)$$

$$\dot{x}(t) = e^{-\delta \cdot t} \cdot \left((\omega_d \cdot C_2 - \delta \cdot C_1) \cdot cos(\omega_d \cdot t) - (\delta \cdot C_2 + \omega_d \cdot C_1) \cdot sin(\omega_d \cdot t)\right)$$

Inserting the initial conditions:

$$x(0) = e^{-\delta \cdot 0} \cdot \left(C_1 \cdot cos(\omega_d \cdot 0) + C_2 \cdot sin(\omega_d \cdot 0)\right) - \frac{g}{{\omega_0}^2} = 0$$

$$0 = C_1 - \frac{g}{{\omega_0}^2}$$

$$C_1 = \frac{g}{{\omega_0}^2}$$

$$\dot{x}(0) = e^{-\delta \cdot 0} \cdot \left((\omega_d \cdot C_2 - \delta \cdot C_1) \cdot cos(\omega_d \cdot 0) - (\delta \cdot C_2 + \omega_d \cdot C_1) \cdot sin(\omega_d \cdot 0)\right) = v_0$$

$$v_0 = \omega_d \cdot C_2 - \delta \cdot C_1$$

$$v_0 = \omega_d \cdot C_2 - \delta \cdot \frac{g}{{\omega_0}^2}$$

$$v_0 + \delta \cdot \frac{g}{{\omega_0}^2} = \omega_d \cdot C_2$$

$$C_2 = \frac{v_0 + \delta \cdot \frac{g}{{\omega_0}^2}}{\omega_d}$$

$$C_2 = \frac{v_0 \cdot {\omega_0}^2 + \delta \cdot g}{\omega_d \cdot {\omega_0}^2}$$

$$x(t) = e^{-\delta \cdot t} \cdot \left(\frac{g}{{\omega_0}^2} \cdot cos(\omega_d \cdot t) + \frac{v_0 \cdot {\omega_0}^2 + \delta \cdot g}{\omega_d \cdot {\omega_0}^2} \cdot sin(\omega_d \cdot t)\right) - \frac{g}{{\omega_0}^2}$$

Result:

$$x = \frac{e^{-\delta \cdot t}}{{\omega_0}^2} \cdot \left(g \cdot \cos(\omega \cdot t) + \frac{v_0 \cdot {\omega_0}^2 + \delta \cdot g}{\omega} \cdot \sin(\omega \cdot t)\right) - \frac{g}{{\omega_0}^2}$$

$$\delta = \frac{c}{2 \cdot m}, \omega_0 = \sqrt{\frac{k}{m}}, \omega = \sqrt{{\omega_0}^2 - \delta^2} \quad (14)$$

$$\dot{x} = e^{-\delta \cdot t} \cdot \left(v_0 \cdot \cos(\omega \cdot t) - \frac{v_0 \cdot \delta + g}{\omega} \cdot \sin(\omega \cdot t)\right)$$

Calculation of MacLaurin series of (14):

Required MacLaurin series:

$$e^x = 1 + x + \frac{x^2}{2} + \frac{x^3}{6} + \cdots$$

$$\sin(x) = x - \frac{x^3}{6} + \cdots$$

$$\cos(x) = 1 - \frac{x^2}{2} - \frac{x^4}{24} + \cdots$$

(All radii of convergence are unlimited.)

$$x = \frac{1}{{\omega_0}^2} \cdot e^{-\delta \cdot t} \cdot \left(g \cdot \cos(\omega \cdot t) + \frac{v_0 \cdot {\omega_0}^2 + \delta \cdot g}{\omega} \cdot \sin(\omega \cdot t)\right) - \frac{g}{{\omega_0}^2} = A \cdot e^{B \cdot t} \cdot (g \cdot \cos(\omega \cdot t) + C \cdot \sin(\omega \cdot t)) + D$$

$$A = \frac{1}{{\omega_0}^2} \qquad B = -\delta$$

$$C = \frac{v_0 \cdot {\omega_0}^2 + \delta \cdot g}{\omega} \quad D = -\frac{g}{{\omega_0}^2}$$

$$x = A \cdot e^{B \cdot t} \cdot (g \cdot \cos(\omega \cdot t) + C \cdot \sin(\omega \cdot t)) + D$$

$$= A \cdot \left(1 + B \cdot t + \frac{B^2}{2} \cdot t^2 + \frac{B^3}{6} \cdot t^3 + \cdots\right) \cdot \left(g \cdot \left(1 - \frac{\omega^2}{2} \cdot t^2 + \cdots\right) + C \cdot \left(\omega \cdot t - \frac{\omega^3}{6} \cdot t^3 + \cdots\right)\right) + D =$$

$$= \left(A + A \cdot B \cdot t + \frac{A \cdot B^2}{2} \cdot t^2 + \frac{A \cdot B^3}{6} \cdot t^3 + \cdots\right) \cdot \left(g - \frac{g \cdot \omega^2}{2} \cdot t^2 + \cdots + C \cdot \omega \cdot t - \frac{C \cdot \omega^3}{6} \cdot t^3 + \cdots\right) + D =$$

$$= \left(A + A \cdot B \cdot t + \frac{A \cdot B^2}{2} \cdot t^2 + \frac{A \cdot B^3}{6} \cdot t^3 + \cdots\right) \cdot \left(g + C \cdot \omega \cdot t - \frac{g \cdot \omega^2}{2} \cdot t^2 - \frac{C \cdot \omega^3}{6} \cdot t^3 + \cdots\right) + D =$$

$$= g \cdot A + g \cdot A \cdot B \cdot t + \frac{g \cdot A \cdot B^2}{2} \cdot t^2 + \frac{g \cdot A \cdot B^3}{6} \cdot t^3 + \cdots + C \cdot \omega \cdot t \cdot A + C \cdot \omega \cdot t \cdot A \cdot B \cdot t + C \cdot \omega \cdot t$$

$$\cdot \frac{A \cdot B^2}{2} \cdot t^2 + C \cdot \omega \cdot t \cdot \frac{A \cdot B^3}{6} \cdot t^3 + \cdots - \frac{g \cdot \omega^2}{2} \cdot t^2 \cdot A - \frac{g \cdot \omega^2}{2} \cdot t^2 \cdot A \cdot B \cdot t - \frac{g \cdot \omega^2}{2} \cdot t^2 \cdot \frac{A \cdot B^2}{2} \cdot t^2 - \frac{g \cdot \omega^2}{2}$$

$$\cdot t^2 \cdot \frac{A \cdot B^3}{6} \cdot t^3 + \cdots - \frac{C \cdot \omega^3}{6} \cdot t^3 \cdot A - \frac{C \cdot \omega^3}{6} \cdot t^3 \cdot A \cdot B \cdot t - \frac{C \cdot \omega^3}{6} \cdot t^3 \cdot \frac{A \cdot B^2}{2} \cdot t^2 - \frac{C \cdot \omega^3}{6} \cdot t^3 \cdot \frac{A \cdot B^3}{6} \cdot t^3$$

$$+ \cdots + D =$$

$$= g \cdot A + g \cdot A \cdot B \cdot t + \frac{g \cdot A \cdot B^2}{2} \cdot t^2 + \frac{g \cdot A \cdot B^3}{6} \cdot t^3 + \cdots + C \cdot \omega \cdot t \cdot A + C \cdot \omega \cdot t \cdot A \cdot B \cdot t + C \cdot \omega \cdot t$$

$$\cdot \frac{A \cdot B^2}{2} \cdot t^2 + \cdots - \frac{g \cdot \omega^2}{2} \cdot t^2 \cdot A - \frac{g \cdot \omega^2}{2} \cdot t^2 \cdot A \cdot B \cdot t + \cdots - \frac{C \cdot \omega^3}{6} \cdot t^3 \cdot A + \cdots + D =$$

$$= (D + g \cdot A) + A \cdot (g \cdot B + C \cdot \omega) \cdot t + A \cdot \left(\frac{g \cdot B^2}{2} - \frac{g \cdot \omega^2}{2} + C \cdot \omega \cdot B\right) \cdot t^2 + A \cdot \left(\frac{g \cdot B^3}{6} + \frac{C \cdot \omega \cdot B^2}{2} - \frac{B \cdot g \cdot \omega^2}{2} - \frac{C \cdot \omega^3}{6}\right)$$

$$\cdot t^3 + \cdots =$$

$$= (D + g \cdot A) + A \cdot (g \cdot B + C \cdot \omega) \cdot t + A \cdot \frac{g \cdot (B^2 - \omega^2) + 2 \cdot C \cdot \omega \cdot B}{2} \cdot t^2 + A$$

$$\cdot \left(\frac{g \cdot B^3}{6} + \frac{C \cdot \omega \cdot B^2}{2} - \frac{B \cdot g \cdot \omega^2}{2} - \frac{C \cdot \omega^3}{6}\right) \cdot t^3 + \cdots =$$

Calculation of the coefficients results in

$$(D + g \cdot A) = -\frac{g}{{\omega_0}^2} + g \cdot \frac{1}{{\omega_0}^2} = 0$$

$$A \cdot (g \cdot B + C \cdot \omega) = \frac{1}{{\omega_0}^2} \cdot \left(-g \cdot \delta + \frac{v_0 \cdot {\omega_0}^2 + \delta \cdot g}{\omega} \cdot \omega\right) = \frac{-g \cdot \delta + v_0 \cdot {\omega_0}^2 + \delta \cdot g}{{\omega_0}^2} = v_0$$

$$A \cdot \frac{g \cdot (B^2 - \omega^2) + 2 \cdot C \cdot \omega \cdot B}{2} = \frac{g \cdot (\delta^2 - \omega^2) + 2 \cdot \frac{v_0 \cdot {\omega_0}^2 + \delta \cdot g}{\omega} \cdot \omega \cdot (-\delta)}{2 \cdot {\omega_0}^2} == \frac{g \cdot (\delta^2 - \omega^2) - 2 \cdot v_0 \cdot {\omega_0}^2 \cdot \delta - 2 \cdot \delta^2 \cdot g}{2 \cdot {\omega_0}^2}$$

$$= \frac{g \cdot (\delta^2 - {\omega_0}^2 + \delta^2) - 2 \cdot v_0 \cdot {\omega_0}^2 \cdot \delta - 2 \cdot \delta^2 \cdot g}{2 \cdot {\omega_0}^2} = -\frac{g \cdot {\omega_0}^2 + 2 \cdot v_0 \cdot {\omega_0}^2 \cdot \delta}{2 \cdot {\omega_0}^2} == -\frac{g + 2 \cdot v_0 \cdot \delta}{2}$$

$$= -\frac{g + 2 \cdot v_0 \cdot \frac{c}{2 \cdot m}}{2} = -\frac{g \cdot m + v_0 \cdot c}{2 \cdot m}$$

$$A \cdot \left(\frac{g \cdot B^3}{6} + \frac{C \cdot \omega \cdot B^2}{2} - \frac{B \cdot g \cdot \omega^2}{2} - \frac{C \cdot \omega^3}{6}\right) =$$

$$= \frac{1}{{\omega_0}^2} \cdot \left(\frac{g \cdot (-\delta)^3}{6} + \frac{\frac{v_0 \cdot {\omega_0}^2 + \delta \cdot g}{\omega} \cdot \omega \cdot \delta^2}{2} - \frac{-\delta \cdot g \cdot \omega^2}{2} - \frac{\frac{v_0 \cdot {\omega_0}^2 + \delta \cdot g}{\omega} \cdot \omega^3}{6}\right) =$$

$$= \frac{1}{{\omega_0}^2} \cdot \left(\frac{g \cdot (-\delta)^3}{6} + \frac{(v_0 \cdot {\omega_0}^2 + \delta \cdot g) \cdot \delta^2}{2} - \frac{-\delta \cdot g \cdot \omega^2}{2} - \frac{(v_0 \cdot {\omega_0}^2 + \delta \cdot g) \cdot \omega^2}{6}\right) =$$

$$= \frac{1}{{\omega_0}^2} \cdot \frac{-g \cdot \delta^3 + 3 \cdot v_0 \cdot {\omega_0}^2 \cdot \delta^2 + 3 \cdot \delta^3 \cdot g + 3 \cdot \delta \cdot g \cdot \omega^2 - v_0 \cdot {\omega_0}^2 \cdot \omega^2 - \delta \cdot g \cdot \omega^2}{6} =$$

$$= \frac{1}{{\omega_0}^2} \cdot \frac{3 \cdot v_0 \cdot {\omega_0}^2 \cdot \delta^2 - v_0 \cdot {\omega_0}^2 \cdot \omega^2 + 2 \cdot \delta^3 \cdot g + 2 \cdot \delta \cdot g \cdot \omega^2}{6} =$$

$$= \frac{1}{{\omega_0}^2} \cdot \frac{{\omega_0}^2 \cdot v_0 \cdot (3 \cdot \delta^2 - \omega^2) + 2 \cdot g \cdot \delta \cdot (\delta^2 + \omega^2)}{6} == \frac{1}{{\omega_0}^2} \cdot \frac{{\omega_0}^2 \cdot v_0 \cdot (3 \cdot \delta^2 - \omega^2) + 2 \cdot g \cdot \delta \cdot {\omega_0}^2}{6}$$

$$= \frac{v_0 \cdot (3 \cdot \delta^2 - \omega^2) + 2 \cdot g \cdot \delta}{6} == \frac{v_0 \cdot \left(4 \cdot \left(\frac{c}{2 \cdot m}\right)^2 - \frac{k}{m}\right) + 2 \cdot g \cdot \frac{c}{2 \cdot m}}{6} = \frac{v_0 \cdot \left(\frac{c^2}{m^2} - \frac{k}{m}\right) + \frac{g \cdot c}{m}}{6} =$$

$$= \frac{v_0 \cdot (c^2 - k \cdot m) + g \cdot c \cdot m}{6 \cdot m^2}$$

Result: Mac Laurin series 3rd degree

$$x(t) = v_0 \cdot t - \frac{g \cdot m + v_0 \cdot c}{2 \cdot m} \cdot t^2 + \frac{c^2 \cdot v_0 + g \cdot c \cdot m - k \cdot m \cdot v_0}{6 \cdot m^2} \cdot t^3$$

In comparison, the limit value l → ∞of the power series approach:

$$x(t) = v_0 \cdot t - \frac{g \cdot m + c \cdot v_0}{2 \cdot m} \cdot t^2 + \frac{c^2 \cdot l^2 \cdot v_0 + g \cdot c \cdot l^2 \cdot m - k \cdot l^2 \cdot m \cdot v_0 - m^2 \cdot {v_0}^3}{6 \cdot l^2 \cdot m^2} \cdot t^3 + \frac{-c^3 \cdot l^2 \cdot v_0 - g \cdot c^2 \cdot l^2 \cdot m + 2 \cdot k \cdot c \cdot l^2 \cdot m \cdot v_0 + 6 \cdot c \cdot m^2 \cdot {v_0}^3 + g \cdot k \cdot l^2 \cdot m^2 + 7 \cdot g \cdot m^3 \cdot {v_0}^2}{24 \cdot l^2 \cdot m^3} \cdot t^4 + \cdots \quad (17)$$

$$\lim_{l\to\infty} x(t) = \lim_{l\to\infty}\left(v_0 \cdot t - \frac{g \cdot m + c \cdot v_0}{2 \cdot m} \cdot t^2 + \frac{c^2 \cdot l^2 \cdot v_0 + g \cdot c \cdot l^2 \cdot m - k \cdot l^2 \cdot m \cdot v_0 - m^2 \cdot {v_0}^3}{6 \cdot l^2 \cdot m^2} \cdot t^3\right) =$$

$$= \lim_{l\to\infty}\left(v_0 \cdot t - \frac{g \cdot m + c \cdot v_0}{2 \cdot m} \cdot t^2 + \frac{c^2 \cdot v_0 + g \cdot c \cdot m - k \cdot m \cdot v_0 - \frac{m^2 \cdot {v_0}^3}{l^2}}{6 \cdot m^2} \cdot t^3\right)$$

$$= v_0 \cdot t - \frac{g \cdot m + c \cdot v_0}{2 \cdot m} \cdot t^2 + \frac{c^2 \cdot v_0 + g \cdot c \cdot m - k \cdot m \cdot v_0}{6 \cdot m^2} \cdot t^3$$

The results match.

# Chapter 2.2.4

*Formulae 19:*

Maximum error estimation for the hammer model

$$x(t) = h - \left(1 - \frac{h^2}{l^2}\right) \cdot \frac{g \cdot t^2}{2}$$

$$\Delta x = \frac{g \cdot t^2 \cdot h^2}{l^3} \cdot \Delta l + \left(1 + \frac{g \cdot h \cdot t^2}{l^2}\right) \cdot \Delta h + g \cdot t \cdot \left(1 - \frac{h^2}{l^2}\right) \cdot \Delta t$$

$$v(t) = -\left(1 - \frac{h^2}{l^2}\right) \cdot g \cdot t \quad (19)$$

$$\Delta v = \frac{2 \cdot g \cdot h^2 \cdot t}{l^3} \cdot \Delta l + \frac{2 \cdot g \cdot h \cdot t}{l^2} \cdot \Delta h + g \cdot \left(1 - \frac{h^2}{l^2}\right) \cdot \Delta t$$

Derivation of the hammer model – speed

$$v(t) = \dot{x}(t) = -\left(1 - \frac{h^2}{l^2}\right) \cdot g \cdot \mathrm{t}$$

Derivation of the error estimates – height

$$x(t) = h - \left(1 - \frac{h^2}{l^2}\right) \cdot \frac{g \cdot t^2}{2}$$

$$\frac{\partial x}{\partial l} = -\frac{h^2 \cdot g \cdot t^2}{l^3}$$

$$\frac{\partial x}{\partial h} = 1 + \frac{h \cdot g \cdot t^2}{l^2}$$

$$\frac{\partial x}{\partial t} = -\left(1 - \frac{h^2}{l^2}\right) \cdot g \cdot \mathrm{t}$$

$$0 < h \le l, 0 < \frac{h}{l} < 1, 0 < \left(\frac{h}{l}\right)^2 < 1, -1 < \frac{h^2}{l^2} - 1 < 0, 0 < 1 - \frac{h^2}{l^2} < 1$$

$$\Delta x = \frac{g \cdot t^2 \cdot h^2}{l^3} \cdot \Delta l + \left(1 + \frac{g \cdot h \cdot t^2}{l^2}\right) \cdot \Delta h + g \cdot t \cdot \left(1 - \frac{h^2}{l^2}\right) \cdot \Delta t$$

Derivation of the error estimates - speed

$$v(t) = -\left(1 - \frac{h^2}{l^2}\right) \cdot g \cdot t$$

$$\frac{\partial v}{\partial l} = -\frac{2 \cdot h^2}{l^3} \cdot g \cdot t$$

$$\frac{\partial v}{\partial h} = \frac{2 \cdot h}{l^2} \cdot g \cdot t$$

$$\frac{\partial v}{\partial t} = -\left(1 - \frac{h^2}{l^2}\right) \cdot g$$

$$\Delta v = \frac{2 \cdot g \cdot h^2 \cdot t}{l^3} \cdot \Delta l + \frac{2 \cdot g \cdot h \cdot t}{l^2} \cdot \Delta h + g \cdot \left(1 - \frac{h^2}{l^2}\right) \cdot \Delta t$$

*Formulae 20:*

Maximum error estimation for the free fall model

$$\begin{gathered} x(t) = h - \frac{g \cdot t^2}{2} \\ \Delta x = \Delta h + \mathrm{g} \cdot \mathrm{t} \cdot \Delta \mathrm{t} \\ v(t) = -g \cdot \mathrm{t} \\ \Delta v = \mathrm{g} \cdot \Delta \mathrm{t} \end{gathered} \tag{20}$$

Derivation of the free fall model – speed

$$v(t) = \dot{x}(t) = -g \cdot \mathrm{t}$$

Derivation of the error estimates - height

$$x(t) = h - \frac{g \cdot t^2}{2}$$

$$\frac{\partial x}{\partial h} = 1$$

$$\frac{\partial x}{\partial t} = -g \cdot t$$

$$\Delta x = \Delta h + \mathrm{g} \cdot \mathrm{t} \cdot \Delta \mathrm{t}$$

Derivation of the error estimates – speed

$$v(t) = -g \cdot \mathrm{t}$$

$$\frac{\partial v}{\partial t} = -g$$

$$\Delta v = \mathrm{g} \cdot \Delta \mathrm{t}$$

*Formulae 21*

Creating the hammer model; maximum error estimation for the hammer model

$$x(t) = h - \left(1 - \frac{h^2}{2 \cdot l^2}\right) \cdot \frac{g \cdot t^2}{2}$$
$$\Delta x = \frac{g \cdot t^2}{2} \cdot \frac{h^2}{l^3} \cdot \Delta l + \left(1 + \frac{g \cdot t^2}{2} \cdot \frac{h}{l^2}\right) \cdot \Delta h + g \cdot t \cdot \left(1 - \frac{h^2}{2 \cdot l^2}\right) \cdot \Delta t$$
$$v(t) = -\left(1 - \frac{h^2}{2 \cdot l^2}\right) \cdot g \cdot t \tag{21}$$
$$\Delta v = \frac{g \cdot h^2 \cdot t}{l^3} \cdot \Delta l + \frac{g \cdot h \cdot t}{l^2} \cdot \Delta h + g \cdot \left(1 - \frac{h^2}{2 \cdot l^2}\right) \cdot \Delta t$$

Derivation of the mean value model: height

$$x(t) = h - \left(1 - \frac{h^2}{l^2}\right) \cdot \frac{g \cdot t^2}{2} \ldots Hammer\ model$$

$$x(t) = h - \frac{g \cdot t^2}{2} \ldots free\ fall$$

Calculation of the mean value model – height

$$x(t) = \frac{h - \left(1 - \frac{h^2}{l^2}\right) \cdot \frac{g \cdot t^2}{2} + h - \frac{g \cdot t^2}{2}}{2} = \frac{2 \cdot h - \left(\left(1 - \frac{h^2}{l^2}\right) + 1\right) \cdot \frac{g \cdot t^2}{2}}{2} = \frac{2 \cdot h - \left(2 - \frac{h^2}{l^2}\right) \cdot \frac{g \cdot t^2}{2}}{2}$$

$$x(t) = h - \left(1 - \frac{h^2}{2 \cdot l^2}\right) \cdot \frac{g \cdot t^2}{2}$$

Calculation of the mean value model – speed

$$v(t) = \dot{x}(t) = -\left(1 - \frac{h^2}{2 \cdot l^2}\right) \cdot \mathrm{g} \cdot \mathrm{t}$$

Derivation of the error estimates – height

$$x(t) = h - \left(1 - \frac{h^2}{2 \cdot l^2}\right) \cdot \frac{g \cdot t^2}{2}$$

$$\frac{\partial v}{\partial l} = -\frac{h^2 \cdot g \cdot t^2}{2 \cdot l^3}$$

$$\frac{\partial v}{\partial h} = 1 + \frac{h \cdot g \cdot t^2}{2 \cdot l^2}$$

$$\frac{\partial v}{\partial t} = -\left(1 - \frac{h^2}{2 \cdot l^2}\right) \cdot g \cdot t$$

$$0 < h \leq l, 0 < \frac{h}{l} < 1, 0 < \left(\frac{h}{l}\right)^2 < 1, 0 < \frac{1}{2} \cdot \frac{h^2}{l^2} < \frac{h^2}{l^2} < 1, -1 < \frac{h^2}{2 \cdot l^2} - 1 < 0, 0 < 1 - \frac{h^2}{2 \cdot l^2} < 1$$

$$\Delta x = \frac{g \cdot t^2}{2} \cdot \frac{h^2}{l^3} \cdot \Delta l + \left(1 + \frac{g \cdot t^2}{2} \cdot \frac{h}{l^2}\right) \cdot \Delta h + g \cdot t \cdot \left(1 - \frac{h^2}{2 \cdot l^2}\right) \cdot \Delta t$$

Derivation of the error estimates – speed

$$v(t) = -\left(1 - \frac{h^2}{2 \cdot l^2}\right) \cdot \mathrm{g} \cdot \mathrm{t}$$

$$\frac{\partial v}{\partial l} = -\frac{h^2}{l^3} \cdot g \cdot t$$

$$\frac{\partial v}{\partial h} = \frac{h}{l^2} \cdot g \cdot t$$

$$\frac{\partial v}{\partial t} = -\left(1 - \frac{h^2}{2 \cdot l^2}\right) \cdot g$$

$$\Delta v = \frac{g \cdot h^2 \cdot t}{l^3} \cdot \Delta l + \frac{g \cdot h \cdot t}{l^2} \cdot \Delta h + g \cdot \left(1 - \frac{h^2}{2 \cdot l^2}\right) \cdot \Delta t$$

*Formulae 22:*

Calculation of the initial height

$$h = \frac{g \cdot {T_0}^2}{2}$$

$$\frac{\Delta h}{h} = 2 \cdot \frac{\Delta T_0}{T_0} \qquad (22)$$

Using (20)

$$x(T_0) = h - \frac{g \cdot {T_0}^2}{2} = 0$$

Result:

$$h = \frac{g \cdot {T_0}^2}{2}$$

Maximum error estimation for the initial height

$$\frac{\partial h}{\partial T_0} = g \cdot T_0$$

$$\Delta h = g \cdot T_0 \cdot \Delta T_0$$

$$\frac{\Delta h}{h} = \frac{g \cdot T_0 \cdot \Delta T_0}{\frac{g \cdot {T_0}^2}{2}}$$

Result:

$$\frac{\Delta h}{h} = 2 \cdot \frac{\Delta T_0}{T_0}$$

*Formulae 23:*

Maximum error estimation for the initial height

$$h = -\frac{l^2}{g \cdot T_0^{\,2}} + \frac{l}{g \cdot T_0^{\,2}} \cdot \sqrt{l^2 + g^2 \cdot T_0^{\,4}}$$

$$\Delta h = \left| \frac{2 \cdot l}{g \cdot T_0^{\,2}} - \frac{2 \cdot l^2 + g^2 \cdot T_0^{\,4}}{g \cdot T_0^{\,2}} \cdot \frac{\sqrt{l^2 + g^2 \cdot T_0^{\,4}}}{l^2 + g^2 \cdot T_0^{\,4}} \right| \cdot \Delta l + \left| \frac{2 \cdot l^2}{g \cdot T_0^{\,3}} - \frac{2 \cdot l^3}{g \cdot T_0^{\,3}} \cdot \frac{\sqrt{l^2 + g^2 \cdot T_0^{\,4}}}{l^2 + g^2 \cdot T_0^{\,4}} \right| \cdot \Delta T_0 \qquad (23)$$

Calculation of the height

Using (19)

$$x(T_0) = h - \left(1 - \frac{h^2}{l^2}\right) \cdot \frac{g \cdot T_0^{\,2}}{2} = 0$$

$$2 \cdot h \cdot l^2 - (l^2 - h^2) \cdot g \cdot T_0^{\,2} = 0$$

$$2 \cdot h \cdot l^2 - l^2 \cdot g \cdot T_0^{\,2} + h^2 \cdot g \cdot T_0^{\,2} = 0$$

$$h^2 \cdot g \cdot T_0^{\,2} + 2 \cdot h \cdot l^2 - l^2 \cdot g \cdot T_0^{\,2} = 0$$

$$h^2 + \frac{2 \cdot l^2}{g \cdot T_0^{\,2}} \cdot h - l^2 = 0$$

$$h_{1,2} = -\frac{l^2}{g \cdot T_0^{\,2}} \pm \sqrt{\left(\frac{l^2}{g \cdot T_0^{\,2}}\right)^2 + l^2}$$

$$h_{1,2} = -\frac{l^2}{g \cdot T_0^{\,2}} \pm \frac{l}{g \cdot T_0^{\,2}} \cdot \sqrt{l^2 + g^2 \cdot T_0^{\,4}}$$

Due to l>0, g>0,

$$h_2 = -\frac{l^2}{g \cdot T_0^{\,2}} - \frac{l}{g \cdot T_0^{\,2}} \cdot \sqrt{l^2 + g^2 \cdot T_0^{\,4}}$$

returns negative values and is therefore discarded.

Result:

$$h = -\frac{l^2}{g \cdot T_0^{\,2}} + \frac{l}{g \cdot T_0^{\,2}} \cdot \sqrt{l^2 + g^2 \cdot T_0^{\,4}}$$

Derivation of the error estimates - height

$$h = -\frac{l^2}{g \cdot T_0^{\,2}} + \frac{l}{g \cdot T_0^{\,2}} \cdot \sqrt{l^2 + g^2 \cdot T_0^{\,4}}$$

The calculation is performed in PTC Mathcad Prime 10.0.1.0:

$$h(T_0) := -\frac{l^2}{g \cdot T_0^{\,2}} + \frac{l}{g \cdot T_0^{\,2}} \cdot \sqrt{l^2 + g^2 \cdot T_0^{\,4}}$$

$$\frac{\partial}{\partial l} h(T_0) \rightarrow \frac{-\left(2 \cdot l \cdot \sqrt{l^2 + T_0^{\,4} \cdot g^2}\right) + 2 \cdot l^2 + T_0^{\,4} \cdot g^2}{T_0^{\,2} \cdot g \cdot \sqrt{l^2 + T_0^{\,4} \cdot g^2}}$$

$$\frac{\partial}{\partial T_0} h(T_0) \rightarrow -\frac{2 \cdot l^3}{T_0^{\,3} \cdot g \cdot \sqrt{l^2 + T_0^{\,4} \cdot g^2}} + \frac{2 \cdot l^2}{T_0^{\,3} \cdot g}$$

Result:

$$\Delta h = \left| \frac{2 \cdot l}{g \cdot T_0^{\,2}} - \frac{2 \cdot l^2 + g^2 \cdot T_0^{\,4}}{g \cdot T_0^{\,2}} \cdot \frac{\sqrt{l^2 + g^2 \cdot T_0^{\,4}}}{l^2 + g^2 \cdot T_0^{\,4}} \right| \cdot \Delta l + \left| \frac{2 \cdot l^2}{g \cdot T_0^{\,3}} - \frac{2 \cdot l^3}{g \cdot T_0^{\,3}} \cdot \frac{\sqrt{l^2 + g^2 \cdot T_0^{\,4}}}{l^2 + g^2 \cdot T_0^{\,4}} \right| \cdot \Delta T_0$$

*Formulae 24:*

Calculation of the height

$$h = -\frac{2 \cdot l^2}{g \cdot T_0^{\,2}} + \frac{l}{g \cdot T_0^{\,2}} \cdot \sqrt{4 \cdot l^2 + 2 \cdot g^2 \cdot T_0^{\,4}}$$

$$\Delta h = \left| \frac{4 \cdot l}{g \cdot T_0^{\,2}} - \frac{8 \cdot l^2 + 2 \cdot g^2 \cdot T_0^{\,4}}{g \cdot T_0^{\,2}} \cdot \frac{\sqrt{4 \cdot l^2 + 2 \cdot g^2 \cdot T_0^{\,4}}}{4 \cdot l^2 + 2 \cdot g^2 \cdot T_0^{\,4}} \right| \cdot \Delta l + \left| \frac{4 \cdot l^2}{g \cdot T_0^{\,3}} - \frac{8 \cdot l^3}{g \cdot T_0^{\,3}} \cdot \frac{\sqrt{4 \cdot l^2 + 2 \cdot g^2 \cdot T_0^{\,4}}}{4 \cdot l^2 + 2 \cdot g^2 \cdot T_0^{\,4}} \right| \cdot \Delta T_0 \tag{24}$$

Calculation of the height

$$x(t) = h - \left(1 - \frac{h^2}{2 \cdot l^2}\right) \cdot \frac{g \cdot t^2}{2}$$

Using (21)

$$x(T_0) = h - \left(1 - \frac{h^2}{2 \cdot l^2}\right) \cdot \frac{g \cdot T_0^{\,2}}{2} = 0$$

$$4 \cdot h \cdot l^2 - (2 \cdot l^2 - h^2) \cdot g \cdot T_0^{\,2} = 0$$

$$4 \cdot h \cdot l^2 - 2 \cdot l^2 \cdot g \cdot T_0^{\,2} + h^2 \cdot g \cdot T_0^{\,2} = 0$$

$$h^2 \cdot g \cdot T_0^{\,2} + 4 \cdot h \cdot l^2 - 2 \cdot l^2 \cdot g \cdot T_0^{\,2} = 0$$

$$h^2 + \frac{4 \cdot l^2}{g \cdot T_0^{\,2}} \cdot h - 2 \cdot l^2 = 0$$

$$h_{1,2} = -\frac{2 \cdot l^2}{g \cdot T_0^{\,2}} \pm \sqrt{\left(\frac{2 \cdot l^2}{g \cdot T_0^{\,2}}\right)^2 + 2 \cdot l^2}$$

$$h_{1,2} = -\frac{2 \cdot l^2}{g \cdot T_0^{\,2}} \pm \frac{l}{g \cdot T_0^{\,2}} \cdot \sqrt{4 \cdot l^2 + 2 \cdot g^2 \cdot T_0^{\,4}}$$

Due to l>0, g>0,

$$h_2 = -\frac{2 \cdot l^2}{g \cdot {T_0}^2} - \frac{l}{g \cdot {T_0}^2} \cdot \sqrt{4 \cdot l^2 + 2 \cdot g^2 \cdot {T_0}^4}$$

returns negative values and is therefore discarded.

Result:

$$h = -\frac{2 \cdot l^2}{g \cdot {T_0}^2} + \frac{l}{g \cdot {T_0}^2} \cdot \sqrt{4 \cdot l^2 + 2 \cdot g^2 \cdot {T_0}^4}$$

Derivation of the error estimates – height

$$h = -\frac{2 \cdot l^2}{g \cdot {T_0}^2} + \frac{l}{g \cdot {T_0}^2} \cdot \sqrt{4 \cdot l^2 + 2 \cdot g^2 \cdot {T_0}^4}$$

The calculation is performed in PTC Mathcad Prime 10.0.1.0:

$$h\left(T_0\right) := -\frac{2 \cdot l^2}{g \cdot {T_0}^2} + \frac{l}{g \cdot {T_0}^2} \cdot \sqrt{4 \cdot l^2 + 2 \cdot g^2 \cdot {T_0}^4}$$

$$\frac{\partial}{\partial l} h\left(T_0\right) \rightarrow \frac{-\left(4 \cdot l \cdot \sqrt{2 \cdot l^2 + {T_0}^4 \cdot g^2}\right) + 4 \cdot \sqrt{2} \cdot l^2 + \sqrt{2} \cdot {T_0}^4 \cdot g^2}{{T_0}^2 \cdot g \cdot \sqrt{2 \cdot l^2 + {T_0}^4 \cdot g^2}}$$

$$\frac{\partial}{\partial T_0} h\left(T_0\right) \rightarrow -\frac{4 \cdot \sqrt{2} \cdot l^3}{{T_0}^3 \cdot g \cdot \sqrt{2 \cdot l^2 + {T_0}^4 \cdot g^2}} + \frac{4 \cdot l^2}{{T_0}^3 \cdot g}$$

Result:

$$\Delta h = \left| \frac{4 \cdot l}{g \cdot {T_0}^2} - \frac{8 \cdot l^2 + 2 \cdot g^2 \cdot {T_0}^4}{g \cdot {T_0}^2} \cdot \frac{\sqrt{4 \cdot l^2 + 2 \cdot g^2 \cdot {T_0}^4}}{4 \cdot l^2 + 2 \cdot g^2 \cdot {T_0}^4} \right| \cdot \Delta l + \left| \frac{4 \cdot l^2}{g \cdot {T_0}^3} - \frac{8 \cdot l^3}{g \cdot {T_0}^3} \cdot \frac{\sqrt{4 \cdot l^2 + 2 \cdot g^2 \cdot {T_0}^4}}{4 \cdot l^2 + 2 \cdot g^2 \cdot {T_0}^4} \right| \cdot \Delta T_0$$

*Formulae 25:*

Calculation of the impact time, impact speed, both derivations – free fall model

$$h = \frac{g \cdot {T_0}^2}{2}$$

Calculation of the impact time

Using (22)

$$T_0 = \sqrt{\frac{2 \cdot h}{g}}$$

Calculation of the speed

$$x(t) = h - \frac{g \cdot t^2}{2}$$

$$\Delta x = \Delta h + \mathrm{g} \cdot \mathrm{t} \cdot \Delta \mathrm{t}$$

(20)

$$v(t) = -g \cdot \mathrm{t}$$

$$\Delta v = \mathrm{g} \cdot \Delta \mathrm{t}$$

Using (20)

$$v_0 = v(T_0) = -g \cdot T_0$$

Derivation of the error estimates – height

$$\frac{\partial T_0}{\partial h} = \frac{1}{2} \cdot \sqrt{\frac{2}{g}} \cdot \frac{1}{\sqrt{h}}$$

$$\frac{\partial T_0}{\partial h} = \frac{1}{2} \cdot \sqrt{\frac{2}{g \cdot h}}$$

$$\Delta T_0 = \frac{1}{2} \cdot \sqrt{\frac{2}{g \cdot h}} \cdot \Delta h = \frac{1}{2} \cdot \sqrt{\frac{2 \cdot h}{g \cdot h^2}} \cdot \Delta h = \frac{1}{2} \cdot \frac{T_0}{h} \cdot \Delta h$$

Result:

$$\frac{\Delta T_0}{T_0} = \frac{1}{2} \cdot \frac{\Delta h}{h}$$

Derivation of the error estimates – speed

$$v_0 = v(T_0) = -g \cdot T_0$$

$$\frac{\partial v_0}{\partial T_0} = -g$$

$$\Delta v_0 = -g \cdot \Delta T_0$$

Result:

$$\frac{\Delta v_0}{|v_0|} = \frac{\Delta T_0}{T_0}$$

*Formulae 26:*

Calculation of the impact time, impact speed, both derivations – hammer model

$$\begin{aligned} T_0 &= \sqrt{\frac{l^2}{l^2 - h^2}} \cdot \sqrt{\frac{2 \cdot h}{g}} \\ \frac{\Delta T_0}{T_0} &= \frac{h^2}{l^2 - h^2} \cdot \frac{\Delta l}{l} + \frac{1}{2} \cdot \frac{l^2 + h^2}{l^2 - h^2} \cdot \frac{\Delta h}{h} \\ v_0 &= -\left(1 - \frac{h^2}{l^2}\right) \cdot g \cdot T_0 \\ \frac{\Delta v_0}{|v_0|} &= \frac{2 \cdot h^2}{l^2 - h^2} \cdot \left(\frac{\Delta h}{h} + \frac{\Delta l}{l}\right) + \frac{\Delta T_0}{T_0} \end{aligned} \tag{26}$$

Calculation of the impact time

Using (19)

$$x(t) = h - \left(1 - \frac{h^2}{l^2}\right) \cdot \frac{g \cdot t^2}{2}$$

$$\Delta x = \frac{g \cdot t^2 \cdot h^2}{l^3} \cdot \Delta l + \left(1 + \frac{g \cdot h \cdot t^2}{l^2}\right) \cdot \Delta h + g \cdot t \cdot \left(1 - \frac{h^2}{l^2}\right) \cdot \Delta t$$

$$v(t) = -\left(1 - \frac{h^2}{l^2}\right) \cdot g \cdot t$$

$$\Delta v = \frac{2 \cdot g \cdot h^2 \cdot t}{l^3} \cdot \Delta l + \frac{2 \cdot g \cdot h \cdot t}{l^2} \cdot \Delta h + g \cdot \left(1 - \frac{h^2}{l^2}\right) \cdot \Delta t$$

$$x(T_0) = h - \left(1 - \frac{h^2}{l^2}\right) \cdot \frac{g \cdot {T_0}^2}{2} = 0$$

$$h = \left(1 - \frac{h^2}{l^2}\right) \cdot \frac{g \cdot {T_0}^2}{2}$$

$$\frac{l^2 - h^2}{l^2} \cdot {T_0}^2 = \frac{2 \cdot h}{g}$$

$${T_0}^2 = \frac{l^2}{l^2 - h^2} \cdot \frac{2 \cdot h}{g}$$

$$T_0 = \sqrt{\frac{l^2}{l^2 - h^2}} \cdot \sqrt{\frac{2 \cdot h}{g}}$$

Calculation of the speed

$$v_0 = v(T_0) = -\left(1 - \frac{h^2}{l^2}\right) \cdot g \cdot T_0$$

Derivation of the error estimates – height

$$T_0 = \sqrt{\frac{l^2}{l^2 - h^2}} \cdot \sqrt{\frac{2 \cdot h}{g}}$$

$$\frac{\partial T_0}{\partial l} = \frac{1}{2} \cdot \frac{1}{\sqrt{\frac{l^2}{l^2 - h^2}}} \cdot \frac{2 \cdot l \cdot (l^2 - h^2) - l^2 \cdot 2 \cdot l}{(l^2 - h^2)^2} \cdot \sqrt{\frac{2 \cdot h}{g}} = \frac{1}{\sqrt{\frac{l^2}{l^2 - h^2}}} \cdot \frac{-l \cdot h^2}{(l^2 - h^2)^2} \cdot \sqrt{\frac{2 \cdot h}{g}}$$

$$= \frac{-h}{l} \cdot \frac{l^2}{l^2 - h^2} \cdot \frac{h}{l^2 - h^2} \cdot \frac{1}{\sqrt{\frac{l^2}{l^2 - h^2}}} \cdot \sqrt{\frac{2 \cdot h}{g}} = \frac{-1}{l} \cdot \frac{h^2}{l^2 - h^2} \cdot \frac{\frac{l^2}{l^2 - h^2}}{\sqrt{\frac{l^2}{l^2 - h^2}}} \cdot \sqrt{\frac{2 \cdot h}{g}}$$

$$= \frac{-1}{l} \cdot \frac{h^2}{l^2 - h^2} \cdot \sqrt{\frac{l^2}{l^2 - h^2}} \cdot \sqrt{\frac{2 \cdot h}{g}} = \frac{-1}{l} \cdot \frac{h^2}{l^2 - h^2} \cdot T_0$$

$$\frac{\partial T_0}{\partial h} = \frac{\partial}{\partial h}\left(\sqrt{\frac{l^2}{l^2-h^2}}\cdot\sqrt{\frac{2\cdot h}{g}}\right) = \frac{\partial}{\partial h}\left(\sqrt{\frac{h}{l^2-h^2}}\cdot\sqrt{\frac{2\cdot l^2}{g}}\right) = \sqrt{\frac{2\cdot l^2}{g}}\cdot\frac{\partial}{\partial h}\left(\sqrt{\frac{h}{l^2-h^2}}\right)$$

$$= \sqrt{\frac{2\cdot l^2}{g}}\cdot\frac{1}{2}\cdot\frac{1}{\sqrt{\frac{h}{l^2-h^2}}}\cdot\frac{l^2-h^2-h\cdot(-2\cdot h)}{(l^2-h^2)^2} = \sqrt{\frac{2\cdot l^2}{g}}\cdot\frac{1}{2}\cdot\frac{1}{\sqrt{\frac{h}{l^2-h^2}}}\cdot\frac{l^2+h^2}{(l^2-h^2)^2}$$

$$= \sqrt{\frac{2\cdot l^2}{g}}\cdot\frac{1}{2}\cdot\frac{1}{\sqrt{\frac{h}{l^2-h^2}}}\cdot\frac{l^2+h^2}{l^2-h^2}\cdot\frac{h}{l^2-h^2}\cdot\frac{1}{h} = \sqrt{\frac{2\cdot l^2}{g}}\cdot\frac{1}{2}\cdot\sqrt{\frac{h}{l^2-h^2}}\cdot\frac{l^2+h^2}{l^2-h^2}\cdot\frac{1}{h}$$

$$= \frac{1}{2}\cdot\frac{l^2+h^2}{l^2-h^2}\cdot\frac{1}{h}\cdot\sqrt{\frac{2\cdot l^2}{g}}\cdot\sqrt{\frac{h}{l^2-h^2}} = \frac{1}{2}\cdot\frac{l^2+h^2}{l^2-h^2}\cdot\frac{1}{h}\cdot\sqrt{\frac{2\cdot h}{g}}\cdot\sqrt{\frac{l^2}{l^2-h^2}} = \frac{1}{2}\cdot\frac{l^2+h^2}{l^2-h^2}\cdot\frac{1}{h}\cdot T_0$$

$$0 < h < l, 0 < h^2 < l^2, l^2-h^2 > 0, \frac{h^2}{l^2-h^2} > 0$$

$$\Delta T_0 = \frac{h^2}{l^2-h^2}\cdot T_0\cdot\frac{\Delta l}{l} + \frac{1}{2}\cdot\frac{l^2+h^2}{l^2-h^2}\cdot T_0\cdot\frac{\Delta h}{h}$$

$$\frac{\Delta T_0}{T_0} = \frac{h^2}{l^2-h^2}\cdot\frac{\Delta l}{l} + \frac{1}{2}\cdot\frac{l^2+h^2}{l^2-h^2}\cdot\frac{\Delta h}{h}$$

Derivation of the error estimates - speed

$$v_0 = -\left(1-\frac{h^2}{l^2}\right)\cdot g\cdot T_0$$

$$\frac{\partial v_0}{\partial T_0} = -\left(1-\frac{h^2}{l^2}\right)\cdot g = \frac{v_0}{T_0}$$

$$\frac{\partial v_0}{\partial h} = \frac{2\cdot h}{l^2}\cdot g\cdot T_0$$

$$\frac{\partial v_0}{\partial l} = -\frac{2\cdot h^2}{l^3}\cdot g\cdot T_0$$

$$\Delta v_0 = \frac{|v_0|}{T_0}\cdot\Delta T_0 + \frac{2\cdot h}{l^2}\cdot g\cdot T_0\cdot\Delta h + \frac{2\cdot h^2}{l^3}\cdot g\cdot T_0\cdot\Delta l = 2\cdot h^2\cdot\frac{g\cdot T_0}{l^2}\cdot\left(\frac{\Delta h}{h}+\frac{\Delta l}{l}\right) + |v_0|\cdot\frac{\Delta T_0}{T_0}$$

$$\frac{\Delta v_0}{|v_0|} = \frac{2\cdot h^2\cdot\frac{g\cdot T_0}{l^2}}{|v_0|}\cdot\left(\frac{\Delta h}{h}+\frac{\Delta l}{l}\right) + \frac{\Delta T_0}{T_0} = \frac{2\cdot h^2\cdot\frac{g\cdot T_0}{l^2}}{\left(1-\frac{h^2}{l^2}\right)\cdot g\cdot T_0}\cdot\left(\frac{\Delta h}{h}+\frac{\Delta l}{l}\right) + \frac{\Delta T_0}{T_0} = \frac{2\cdot h^2}{\left(1-\frac{h^2}{l^2}\right)\cdot l^2}\cdot\left(\frac{\Delta h}{h}+\frac{\Delta l}{l}\right) + \frac{\Delta T_0}{T_0}$$

$$= \frac{2\cdot h^2}{l^2-h^2}\cdot\left(\frac{\Delta h}{h}+\frac{\Delta l}{l}\right) + \frac{\Delta T_0}{T_0}$$

$$\frac{\Delta v_0}{|v_0|} = \frac{2\cdot h^2}{l^2-h^2}\cdot\left(\frac{\Delta h}{h}+\frac{\Delta l}{l}\right) + \frac{\Delta T_0}{T_0}$$

*Formulae 27:*

$$T_0 = \sqrt{\frac{2\cdot l^2}{2\cdot l^2-h^2}}\cdot\sqrt{\frac{2\cdot h}{g}}$$

$$\frac{\Delta T_0}{T_0} = \frac{h^2}{2\cdot l^2-h^2}\cdot\frac{\Delta l}{l} + \frac{1}{2}\cdot\frac{2\cdot l^2+h^2}{2\cdot l^2-h^2}\cdot\frac{\Delta h}{h}$$

$$v_0 = -\left(1 - \frac{h^2}{2 \cdot l^2}\right) \cdot g \cdot T_0$$

$$\frac{\Delta v_0}{|v_0|} = \frac{2 \cdot h^2}{2 \cdot l^2 - h^2} \cdot \left(\frac{\Delta h}{h} + \frac{\Delta l}{l}\right) + \frac{\Delta T_0}{T_0}$$

Using (21)

$$x(t) = h - \left(1 - \frac{h^2}{2 \cdot l^2}\right) \cdot \frac{g \cdot t^2}{2}$$

$$\Delta x = \frac{g \cdot t^2}{2} \cdot \frac{h^2}{l^3} \cdot \Delta l + \left(1 + \frac{g \cdot t^2}{2} \cdot \frac{h}{l^2}\right) \cdot \Delta h + g \cdot t \cdot \left(1 - \frac{h^2}{2 \cdot l^2}\right) \cdot \Delta t$$

$$v(t) = -\left(1 - \frac{h^2}{2 \cdot l^2}\right) \cdot g \cdot t$$

$$\Delta v = \frac{g \cdot h^2 \cdot t}{l^3} \cdot \Delta l + \frac{g \cdot h \cdot t}{l^2} \cdot \Delta h + g \cdot \left(1 - \frac{h^2}{2 \cdot l^2}\right) \cdot \Delta t$$

Calculation of the impact time

$$x(T_0) = h - \left(1 - \frac{h^2}{2 \cdot l^2}\right) \cdot \frac{g \cdot {T_0}^2}{2} = 0$$

$$h - \left(1 - \frac{h^2}{2 \cdot l^2}\right) \cdot \frac{g \cdot {T_0}^2}{2} = 0$$

$$h = \frac{2 \cdot l^2 - h^2}{2 \cdot l^2} \cdot \frac{g \cdot {T_0}^2}{2}$$

$${T_0}^2 = \frac{2 \cdot l^2}{2 \cdot l^2 - h^2} \cdot \frac{2 \cdot h}{g}$$

$$T_0 = \sqrt{\frac{2 \cdot l^2}{2 \cdot l^2 - h^2}} \cdot \sqrt{\frac{2 \cdot h}{g}}$$

Calculation of the speed

$$v_0 = v(T_0) = -\left(1 - \frac{h^2}{2 \cdot l^2}\right) \cdot g \cdot T_0$$

$$v_0 = -\left(1 - \frac{h^2}{2 \cdot l^2}\right) \cdot g \cdot T_0$$

Derivation of the error estimates – impact time

$$T_0 = \sqrt{\frac{2 \cdot l^2}{2 \cdot l^2 - h^2}} \cdot \sqrt{\frac{2 \cdot h}{g}}$$

$$\frac{\partial T_0}{\partial l} = \sqrt{\frac{2 \cdot h}{g}} \cdot \frac{1}{2} \cdot \frac{\frac{4 \cdot l \cdot (2 \cdot l^2 - h^2) - 2 \cdot l^2 \cdot 4 \cdot l}{(2 \cdot l^2 - h^2)^2}}{\sqrt{\frac{2 \cdot l^2}{2 \cdot l^2 - h^2}}} = \sqrt{\frac{2 \cdot l^2}{2 \cdot l^2 - h^2}} \cdot \sqrt{\frac{2 \cdot h}{g}} \cdot \frac{1}{2} \cdot \frac{\frac{4 \cdot l \cdot (2 \cdot l^2 - h^2) - 2 \cdot l^2 \cdot 4 \cdot l}{(2 \cdot l^2 - h^2)^2}}{\frac{2 \cdot l^2}{2 \cdot l^2 - h^2}}$$

$$= \sqrt{\frac{2 \cdot l^2}{2 \cdot l^2 - h^2}} \cdot \sqrt{\frac{2 \cdot h}{g}} \cdot \frac{1}{2} \cdot \frac{\frac{-4 \cdot l \cdot h^2}{2 \cdot l^2 - h^2}}{2 \cdot l^2} = -\sqrt{\frac{2 \cdot l^2}{2 \cdot l^2 - h^2}} \cdot \sqrt{\frac{2 \cdot h}{g}} \cdot \frac{h^2}{2 \cdot l^2 - h^2} \cdot \frac{1}{l} = -T_0 \cdot \frac{h^2}{2 \cdot l^2 - h^2} \cdot \frac{1}{l}$$

$$\frac{\partial T_0}{\partial h}=\frac{\partial}{\partial h}\left(\sqrt{\frac{2\cdot h}{2\cdot l^2-h^2}}\cdot\sqrt{\frac{2\cdot l^2}{g}}\right)=\sqrt{\frac{2\cdot l^2}{g}}\cdot\frac{1}{2}\cdot\frac{\frac{2\cdot(2\cdot l^2-h^2)+2\cdot h\cdot 2\cdot h}{(2\cdot l^2-h^2)^2}}{\sqrt{\frac{2\cdot h}{2\cdot l^2-h^2}}}=\sqrt{\frac{2\cdot l^2}{g}}\cdot\frac{1}{2}\cdot\frac{\frac{4\cdot l^2+2\cdot h^2}{(2\cdot l^2-h^2)^2}}{\sqrt{\frac{2\cdot h}{2\cdot l^2-h^2}}}$$

$$=\sqrt{\frac{2\cdot l^2}{g}}\cdot\frac{\frac{2\cdot l^2+h^2}{(2\cdot l^2-h^2)^2}}{\sqrt{\frac{2\cdot h}{2\cdot l^2-h^2}}}=\sqrt{\frac{2\cdot l^2}{g}}\cdot\frac{\frac{2\cdot l^2+h^2}{(2\cdot l^2-h^2)^2}}{\sqrt{\frac{2\cdot h}{2\cdot l^2-h^2}}}\cdot\frac{\sqrt{\frac{2\cdot h}{2\cdot l^2-h^2}}}{\sqrt{\frac{2\cdot h}{2\cdot l^2-h^2}}}=\sqrt{\frac{2\cdot l^2}{g}}\cdot\frac{\frac{2\cdot l^2+h^2}{(2\cdot l^2-h^2)^2}}{\frac{2\cdot h}{2\cdot l^2-h^2}}\cdot\sqrt{\frac{2\cdot h}{2\cdot l^2-h^2}}$$

$$=\sqrt{\frac{2\cdot h}{2\cdot l^2-h^2}}\cdot\sqrt{\frac{2\cdot l^2}{g}}\cdot\frac{\frac{2\cdot l^2+h^2}{(2\cdot l^2-h^2)^2}}{\frac{2\cdot h}{2\cdot l^2-h^2}}=\sqrt{\frac{2\cdot l^2}{2\cdot l^2-h^2}}\cdot\sqrt{\frac{2\cdot h}{g}}\cdot\frac{2\cdot l^2+h^2}{2\cdot l^2-h^2}\cdot\frac{1}{2\cdot h}=T_0\cdot\frac{2\cdot l^2+h^2}{2\cdot l^2-h^2}\cdot\frac{1}{2\cdot h}$$

$$\Delta T_0=T_0\cdot\frac{h^2}{2\cdot l^2-h^2}\cdot\frac{1}{l}\cdot\Delta l+T_0\cdot\frac{2\cdot l^2+h^2}{2\cdot l^2-h^2}\cdot\frac{1}{2\cdot h}\cdot\Delta h$$

$$\frac{\Delta T_0}{T_0}=\frac{h^2}{2\cdot l^2-h^2}\cdot\frac{\Delta l}{l}+\frac{1}{2}\cdot\frac{2\cdot l^2+h^2}{2\cdot l^2-h^2}\cdot\frac{\Delta h}{h}$$

Derivation of the error estimates – impact speed

$$v_0=-\left(1-\frac{h^2}{2\cdot l^2}\right)\cdot g\cdot T_0$$

$$\frac{\partial v_0}{\partial T_0}=-\left(1-\frac{h^2}{2\cdot l^2}\right)\cdot g=-\left(1-\frac{h^2}{2\cdot l^2}\right)\cdot g\cdot T_0\cdot\frac{1}{T_0}=\frac{v_0}{T_0}$$

$$\frac{\partial v_0}{\partial h}=\frac{h}{l^2}\cdot g\cdot T_0$$

$$\frac{\partial v_0}{\partial l}=-\frac{h^2}{l^3}\cdot g\cdot T_0$$

$$\Delta v_0=\frac{h}{l^2}\cdot g\cdot T_0\cdot\Delta h+\frac{h^2}{l^3}\cdot g\cdot T_0\cdot\Delta l+\frac{|v_0|}{T_0}\cdot\Delta T_0$$

$$\frac{\Delta v_0}{|v_0|}=\frac{h}{l^2}\cdot\frac{g\cdot T_0}{|v_0|}\cdot\Delta h+\frac{h^2}{l^3}\cdot\frac{g}{|v_0|}\cdot T_0\cdot\Delta l+\frac{\Delta T_0}{T_0}$$

$$\frac{\Delta v_0}{|v_0|}=\frac{h^2}{l^2}\cdot\frac{1}{\left(1-\frac{h^2}{2\cdot l^2}\right)}\cdot\frac{\Delta h}{h}+\frac{h^2}{l^2}\cdot\frac{1}{\left(1-\frac{h^2}{2\cdot l^2}\right)}\cdot\frac{\Delta l}{l}+\frac{\Delta T_0}{T_0}$$

$$\frac{\Delta v_0}{|v_0|}=\frac{h^2}{l^2}\cdot\frac{2\cdot l^2}{2\cdot l^2-h^2}\cdot\left(\frac{\Delta h}{h}+\frac{\Delta l}{l}\right)+\frac{\Delta T_0}{T_0}$$

$$\frac{\Delta v_0}{|v_0|}=\frac{2\cdot h^2}{2\cdot l^2-h^2}\cdot\left(\frac{\Delta h}{h}+\frac{\Delta l}{l}\right)+\frac{\Delta T_0}{T_0}$$

## Chapter 2.2.5

*Formulae 29:*

$$p(t) = v_0 \cdot t - \frac{a_{max} \cdot tp_0 + 2 \cdot v_0}{2 \cdot tp_0} \cdot t^2 + \frac{a_{max} \cdot tp_0 + v_0}{3 \cdot {tp_0}^2} \cdot t^3 \tag{29}$$

$$p(0) = 0 \quad \dot{p}(0) = v_0 < 0 \quad \dot{p}(t_{P0}) = 0 \quad \ddot{p}(t_{P0}) = a_{max} > 0$$

General approach

$$p(t) = a \cdot t^3 + b \cdot t^2 + c \cdot t + d$$
$$\dot{p}(t) = 3 \cdot a \cdot t^2 + 2 \cdot b \cdot t + c$$
$$\ddot{p}(t) = 6 \cdot a \cdot t + 2 \cdot b$$

$$p(0) = a \cdot 0^3 + b \cdot 0^2 + c \cdot 0 + d = 0$$
$$\dot{p}(0) = 3 \cdot a \cdot 0^2 + 2 \cdot b \cdot 0 + c = v_0$$
$$\dot{p}(t_{P0}) = 3 \cdot a \cdot {t_{P0}}^2 + 2 \cdot b \cdot t_{P0} + c = 0$$
$$\underline{\ddot{p}(t_{P0}) = 6 \cdot a \cdot t_{P0} + 2 \cdot b = a_{max}}$$

$$d = 0$$
$$c = v_0$$
$$3 \cdot a \cdot {t_{P0}}^2 + 2 \cdot b \cdot t_{P0} + c = 0$$
$$\underline{6 \cdot a \cdot t_{P0} + 2 \cdot b = a_{max}}$$

$$d = 0$$
$$c = v_0$$
$$3 \cdot a \cdot {t_{P0}}^2 + 2 \cdot b \cdot t_{P0} = -v_0$$
$$\underline{6 \cdot a \cdot {t_{P0}}^2 + 2 \cdot b \cdot t_{P0} = a_{max} \cdot t_{P0}}$$

$$d = 0$$
$$c = v_0$$
$$3 \cdot a \cdot {t_{P0}}^2 + 2 \cdot b \cdot t_{P0} = -v_0$$
$$\underline{3 \cdot a \cdot {t_{P0}}^2 = a_{max} \cdot t_{P0} + v_0}$$

$$d = 0$$
$$c = v_0$$
$$a = \frac{a_{max} \cdot t_{P0} + v_0}{3 \cdot {t_{P0}}^2}$$
$$\underline{3 \cdot a \cdot {t_{P0}}^2 + 2 \cdot b \cdot t_{P0} + v_0 = 0}$$

$$d = 0$$
$$c = v_0$$
$$b = -\frac{v_0 + 3 \cdot a \cdot {t_{P0}}^2}{2 \cdot t_{P0}} = -\frac{v_0 + 3 \cdot \frac{a_{max} \cdot t_{P0} + v_0}{3 \cdot {t_{P0}}^2} \cdot {t_{P0}}^2}{2 \cdot t_{P0}} = -\frac{a_{max} \cdot t_{P0} + 2 \cdot v_0}{2 \cdot t_{P0}}$$

$$a = \frac{a_{max} \cdot t_{P0} - v_0}{3 \cdot {t_{P0}}^2}$$

Result:

$$p(t) = v_0 \cdot t - \frac{a_{max} \cdot tp_0 + 2 \cdot v_0}{2 \cdot tp_0} \cdot t^2 + \frac{a_{max} \cdot tp_0 - v_0}{3 \cdot {tp_0}^2} \cdot t^3$$

*Formulae 30:*

$$h_{min} = p(t_{P0}) = \frac{t_{P0}}{6} \cdot (2 \cdot v_0 - a_{max} \cdot t_{P0})$$
$$\Delta h_{min} = \Delta p = \frac{|v_0 - a_{max} \cdot t_{P0}|}{3} \cdot \Delta t_{P0} + \frac{t_{P0}}{3} \cdot \Delta v_0 + \frac{{t_{P0}}^2}{6} \cdot \Delta a_{max}$$

Derivation of the error estimates – height

$$h_{min} = \frac{t_{P0}}{6} \cdot (2 \cdot v_0 - a_{max} \cdot t_{P0})$$

$$\frac{\partial h_{min}}{\partial t_{P0}} = \frac{1}{3} \cdot (v_0 - a_{max} \cdot t_{P0})$$

$$\frac{\partial h_{min}}{\partial v_0} = \frac{t_{P0}}{3}$$

$$\frac{\partial h_{min}}{\partial a_{max}} = -\frac{{t_{P0}}^2}{6}$$

$$\Delta h_{min} = \frac{1}{3} \cdot |v_0 - a_{max} \cdot t_{P0}| \cdot \Delta t_{P0} + \frac{t_{P0}}{3} \cdot \Delta v_0 + \frac{{t_{P0}}^2}{6} \cdot \Delta a_{max}$$

*Formulae 31:*

$$q(t) = -\frac{a_{max} \cdot (d_0 - t_{P0})^2 + 2 \cdot h_{min}}{2 \cdot (d_0 - t_{P0})^3} \cdot t^3 + \frac{a_{max}}{2} \cdot t^2 + h_{min}$$

$$q(0) = h_{min} \quad \dot{q}(0) = 0 \quad q(d_0 - t_{P0}) = 0 \quad \ddot{q}(0) = a_{max}$$

General approach

$$q(t) = a \cdot t^3 + b \cdot t^2 + c \cdot t + d$$
$$\dot{q}(t) = 3 \cdot a \cdot t^2 + 2 \cdot b \cdot t + c$$
$$\ddot{q}(t) = 6 \cdot a \cdot t + 2 \cdot b$$

$$q(0) = a \cdot 0^3 + b \cdot 0^2 + c \cdot 0 + d = h_{min}$$
$$\dot{q}(0) = 3 \cdot a \cdot 0^2 + 2 \cdot b \cdot 0 + c = 0$$
$$q(d_0 - t_{P0}) = a \cdot (d_0 - t_{P0})^3 + b \cdot (d_0 - t_{P0})^2 + c \cdot (d_0 - t_{P0}) + d = 0$$
$$\ddot{q}(0) = 6 \cdot a \cdot 0 + 2 \cdot b = a_{max}$$

$$d = h_{min}$$
$$c = 0$$
$$a \cdot (d_0 - t_{P0})^3 + b \cdot (d_0 - t_{P0})^2 + h_{min} = 0$$

$$\sout{2 \cdot b = a_{max}}$$

$$d = h_{min}$$
$$c = 0$$
$$a = -\frac{b \cdot (d_0 - t_{P0})^2 + h_{min}}{(d_0 - t_{P0})^3} \cdot \frac{2}{2} = -\frac{a_{max} \cdot (d_0 - t_{P0})^2 + 2 \cdot h_{min}}{2 \cdot (d_0 - t_{P0})^3}$$
$$\sout{2 \cdot b = a_{max}}$$

Result:

$$q(t) = -\frac{a_{max} \cdot (d_0 - t_{P0})^2 + 2 \cdot h_{min}}{2 \cdot (d_0 - t_{P0})^3} \cdot t^3 + \frac{a_{max}}{2} \cdot t^2 + h_{min}$$

*Formulae 32:*

$$v_1 = \dot{q}(d_0 - t_{P0}) = -\frac{a_{max} \cdot (d_0 - t_{P0})^2 + 6 \cdot h_{min}}{2 \cdot (d_0 - t_{P0})} = -\frac{a_{max} \cdot (d_0 - t_{P0})}{2} - \frac{3 \cdot h_{min}}{d_0 - t_{P0}}$$
$$\Delta v_1 = \frac{d_0 - t_{P0}}{2} \cdot \Delta a_{max} + \frac{3}{d_0 - t_{P0}} \cdot \Delta h_{min} + \frac{1}{2} \cdot \left| \frac{a_{max} \cdot (d_0 - t_{P0})^2 - 6 \cdot h_{min}}{(d_0 - t_{P0})^2} \right| \cdot (\Delta d_0 + \Delta t_{P0}) \quad (32)$$

Uses (31)

$$q(t) = -\frac{a_{max} \cdot (d_0 - t_{P0})^2 + 2 \cdot h_{min}}{2 \cdot (d_0 - t_{P0})^3} \cdot t^3 + \frac{a_{max}}{2} \cdot t^2 + h_{min}$$

Calculation of the speed

$$\dot{q}(t) = -3 \cdot \frac{a_{max} \cdot (d_0 - t_{P0})^2 + 2 \cdot h_{min}}{2 \cdot (d_0 - t_{P0})^3} \cdot t^2 + a_{max} \cdot t$$

$$\dot{q}(d_0 - t_{P0}) = -3 \cdot \frac{a_{max} \cdot (d_0 - t_{P0})^2 + 2 \cdot h_{min}}{2 \cdot (d_0 - t_{P0})^3} \cdot (d_0 - t_{P0})^2 + a_{max} \cdot (d_0 - t_{P0})$$
$$= -3 \cdot \frac{a_{max} \cdot (d_0 - t_{P0})^2 + 2 \cdot h_{min}}{2 \cdot (d_0 - t_{P0})} + a_{max} \cdot (d_0 - t_{P0})$$
$$= -3 \cdot \frac{a_{max} \cdot (d_0 - t_{P0})}{2} - 3 \cdot \frac{h_{min}}{d_0 - t_{P0}} + 2 \cdot \frac{a_{max} \cdot (d_0 - t_{P0})}{2} = -\frac{a_{max} \cdot (d_0 - t_{P0})}{2} - 3 \cdot \frac{h_{min}}{d_0 - t_{P0}}$$

$$v_1 = \dot{q}(d_0 - t_{P0}) = -\frac{a_{max} \cdot (d_0 - t_{P0})^2 + 6 \cdot h_{min}}{2 \cdot (d_0 - t_{P0})} = -\frac{a_{max} \cdot (d_0 - t_{P0})}{2} - \frac{3 \cdot h_{min}}{d_0 - t_{P0}}$$

Derivation of the error estimates – impact speed

$$v_1 = -\frac{a_{max} \cdot (d_0 - t_{P0})}{2} - \frac{3 \cdot h_{min}}{d_0 - t_{P0}}$$

$$\frac{\partial v_1}{\partial a_{max}} = -\frac{d_0 - t_{P0}}{2}$$

$$\frac{\partial v_1}{\partial h_{min}} = -\frac{3}{d_0 - t_{P0}}$$

$$\frac{\partial v_1}{\partial d_0} = -\frac{a_{max}}{2} + \frac{3 \cdot h_{min}}{(d_0 - t_{P0})^2} = -1 \cdot \frac{a_{max} \cdot (d_0 - t_{P0})^2 - 6 \cdot h_{min}}{2 \cdot (d_0 - t_{P0})^2}$$

$$\frac{\partial v_1}{\partial t_{P0}} = \frac{a_{max}}{2} - \frac{3 \cdot h_{min}}{(d_0 - t_{P0})^2} = \frac{a_{max} \cdot (d_0 - t_{P0})^2 - 6 \cdot h_{min}}{2 \cdot (d_0 - t_{P0})^2}$$

Result:

$$\Delta v_1 = \frac{d_0 - t_{P0}}{2} \cdot \Delta a_{max} + \frac{3}{d_0 - t_{P0}} \cdot \Delta h_{min} + \frac{1}{2} \cdot \left| \frac{a_{max} \cdot (d_0 - t_{P0})^2 - 6 \cdot h_{min}}{(d_0 - t_{P0})^2} \right| \cdot (\Delta d_0 + \Delta t_{P0})$$

## Chapter 2.2.6

*Formulae 34:*

$$t_{max} = \frac{v_1}{g} \qquad \frac{\Delta t_{max}}{t_{max}} = \frac{\Delta v_1}{v_1}$$
$$h_{max} = \frac{1}{2} \cdot \frac{{v_1}^2}{g} \qquad \frac{\Delta h_{max}}{h_{max}} = 2 \cdot \frac{\Delta v_1}{v_1} \tag{34}$$

Uses (33)

$$x(t) = -\frac{g}{2} \cdot t^2 + v_1 \cdot t \qquad v_1 > 0 \tag{33}$$

Calculation of $t_{max}$

$$\dot{x}(t_{max}) = -g \cdot t_{max} + v_1 = 0$$

Result:

$$t_{max} = \frac{v_1}{g}$$

Calculation of $h_{max}$

$$x(t_{max}) = -\frac{g}{2} \cdot {t_{max}}^2 + v_1 \cdot t_{max} = -\frac{g}{2} \cdot \left(\frac{v_1}{g}\right)^2 + v_1 \cdot \frac{v_1}{g} = -\frac{{v_1}^2}{2 \cdot g} + \frac{{v_1}^2}{g} = \frac{1}{2} \cdot \frac{{v_1}^2}{g}$$

Result:

$$h_{max} = \frac{1}{2} \cdot \frac{{v_1}^2}{g}$$

Derivation of the error estimates – $t_{max}$

$$t_{max} = \frac{v_1}{g}$$

$$\frac{\partial t_{max}}{\partial v_1} = \frac{1}{g}$$

$$\Delta t_{max} = \frac{1}{g} \cdot \Delta v_1$$

$$\frac{\Delta t_{max}}{t_{max}} = \frac{\frac{\Delta v_1}{g}}{\frac{v_1}{g}}$$

$$\frac{\Delta t_{max}}{t_{max}} = \frac{\Delta v_1}{v_1}$$

Derivation of the error estimates – $h_{max}$

$$h_{max} = \frac{1}{2} \cdot \frac{{v_1}^2}{g}$$

$$\frac{\partial h_{max}}{\partial v_1} = \frac{v_1}{g}$$

$$\Delta h_{max} = \frac{v_1}{g} \cdot \Delta v_1$$

$$\frac{\Delta h_{max}}{h_{max}} = \frac{\frac{v_1 \cdot \Delta v_1}{g}}{\frac{1}{2} \cdot \frac{{v_1}^2}{g}}$$

$$\frac{\Delta h_{max}}{h_{max}} = 2 \cdot \frac{\Delta v_1}{v_1}$$

*Formulae 35:*

$$x(0) = 0 \quad \dot{x}(0) = v_1 \quad x(2 \cdot T_1) = 0, \quad 0 \leq t \leq T_1$$

$$x(t) = -\frac{v_1}{2 \cdot T_1} \cdot t^2 + v_1 \cdot t \qquad \Delta x = v_1 \cdot \left(1 - \frac{t}{T_1}\right) \cdot \Delta t + t \cdot \left(1 - \frac{t}{2 \cdot T_1}\right) \cdot \Delta v_1 + \frac{v_1 \cdot t^2}{2 \cdot {T_1}^2} \cdot \Delta T_1 \tag{35}$$

Calculation of height

General approach:

$$x(t) = a \cdot t^2 + b \cdot t + c$$
$$\dot{x}(t) = 2 \cdot a \cdot t + b$$

$$x(0) = a \cdot 0^2 + b \cdot 0 + c = 0$$
$$\dot{x}(0) = 2 \cdot a \cdot 0 + b = v_1$$
$$\underline{x(2 \cdot T_1) = a \cdot (2 \cdot T_1)^2 + b \cdot 2 \cdot T_1 + c = 0}$$

$$c = 0$$
$$b = v_1$$
$$\underline{4 \cdot a \cdot {T_1}^2 + b \cdot 2 \cdot T_1 + c = 0}$$

$$c = 0$$
$$b = v_1$$
$$\underline{4 \cdot a \cdot {T_1}^2 + v_1 \cdot 2 \cdot T_1 = 0}$$

$$c = 0$$
$$b = v_1$$
$$\underline{4 \cdot a \cdot {T_1}^2 + v_1 \cdot 2 \cdot T_1 = 0}$$

$$c = 0$$
$$b = v_1$$
$$\underline{2 \cdot a \cdot {T_1}^2 = -v_1 \cdot T_1, \;\; T_1 \neq 0}$$

$$a = \frac{-v_1}{2 \cdot T_1}$$
$$c = 0$$
$$\underline{b = v_1}$$

$$x(t) = -\frac{v_1}{2 \cdot T_1} \cdot t^2 + v_1 \cdot t$$

Derivation of the error estimates – height

$$x(t) = -\frac{v_1}{2 \cdot T_1} \cdot t^2 + v_1 \cdot t$$

$$\frac{\partial x}{\partial t} = -\frac{v_1}{T_1} \cdot t + v_1$$

$$\frac{\partial x}{\partial v_1} = -\frac{t^2}{2 \cdot T_1} + t$$

$$\frac{\partial x}{\partial T_1} = \frac{v_1}{2 \cdot {T_1}^2} \cdot t^2$$

$$\Delta x = \left| -\frac{v_1}{T_1} \cdot t + v_1 \right| \cdot \Delta t + \left| -\frac{t^2}{2 \cdot T_1} + t \right| \cdot \Delta v_1 + \frac{v_1}{2 \cdot {T_1}^2} \cdot t^2 \cdot \Delta T_1$$

Result:

$$\Delta x = v_1 \cdot \left(1 - \frac{t}{T_1}\right) \cdot \Delta t + t \cdot \left(1 - \frac{t}{2 \cdot T_1}\right) \cdot \Delta v_1 + \frac{v_1 \cdot t^2}{2 \cdot {T_1}^2} \cdot \Delta T_1$$

Maximum at time $T_1$:
Result:

$$x(T_1) = -\frac{v_1}{2 \cdot T_1} \cdot {T_1}^2 + v_1 \cdot T_1 = -\frac{v_1}{2} \cdot T_1 + v_1 \cdot T_1 = \frac{v_1}{2} \cdot T_1$$

# Formulae 36:

$$\frac{v_1}{T_1} = g \qquad \frac{\Delta T_1}{T_1} = \frac{\Delta v_1}{v_1} \tag{36}$$

Calculation of maximal height

Uses (33), (34), (35)

$$h_{max} = \frac{1}{2} \cdot \frac{{v_1}^2}{g}$$

$$h_{max} = \frac{v_1 \cdot T_1}{2}$$

$$h_{max} = \frac{1}{2} \cdot \frac{{v_1}^2}{g} = \frac{v_1 \cdot T_1}{2}$$

$$\frac{v_1}{T_1} = g$$

Derivation of the error estimates – maximum height

$$\frac{v_1}{T_1} = g$$

$$T_1 = \frac{v_1}{g}$$

$$\frac{\partial T_1}{\partial v_1} = \frac{1}{g}$$

$$\Delta T_1 = \frac{1}{g} \cdot \Delta v_1 = \frac{T_1}{v} \cdot \Delta v_1$$

Result:

$$\frac{\Delta T_1}{T_1} = \frac{\Delta v_1}{v_1}$$

# Chapter 2.3.1

*Formulae 37:*

$$E_r = \frac{h_{max}}{h} = \frac{{v_1}^2}{2 \cdot g \cdot h} \qquad \frac{\Delta E_r}{E_r} = \frac{\Delta h}{h} + 2 \cdot \frac{\Delta v_1}{v_1} \tag{37}$$

Uses (34)

$$h_{max} = \frac{{v_1}^2}{2 \cdot g}$$

$$E_r = \frac{h_{max}}{h} = \frac{{v_1}^2}{2 \cdot g \cdot h}$$

# Chapter 2.3.2

*Formulae 39*

$$c_s = -\frac{a_{max} + g}{v_0} \cdot m$$

$$\Delta c_s = \left|\frac{m}{v_0}\right| \cdot \Delta a_{max} + \left|\frac{a_{max} + g}{{v_0}^2} \cdot m\right| \cdot \Delta v_0 + \left|\frac{a_{max} + g}{v_0}\right| \cdot \Delta m \tag{39}$$

Uses (38)

$$x(t) = v_0 \cdot t - \frac{g \cdot m + c \cdot v_0}{2 \cdot m} \cdot t^2 \tag{38}$$

$$\ddot{x}(t_{P0}) = a_{max}$$

Calculation of $c_s$

$$x(t) = v_0 \cdot t - \frac{g \cdot m + c \cdot v_0}{2 \cdot m} \cdot t^2$$

$$\dot{x}(t) = v_0 - \frac{g \cdot m + c \cdot v_0}{m} \cdot t$$

$$\ddot{x}(t) = -\frac{g \cdot m + c \cdot v_0}{m}$$

$$\ddot{x}(t_{P0}) = -\frac{g \cdot m + c_s \cdot v_0}{m} = a_{max}$$

$$-\frac{g \cdot m + c_s \cdot v_0}{m} = a_{max}$$

$$g \cdot m + c_s \cdot v_0 = -m \cdot a_{max}$$

$$c_s \cdot v_0 = -m \cdot a_{max} - g \cdot m$$

$$c_s \cdot v_0 = -m \cdot (a_{max} + g)$$

Result:

$$c_s = -\frac{a_{max} + g}{v_0} \cdot m$$

$$c_s = -\frac{G_{max} + 1}{v_0} \cdot g \cdot m$$

Derivation of the error estimates – $c_s$

$$c_s = -\frac{a_{max} + g}{v_0} \cdot m$$

$$\frac{\partial c_s}{\partial a_{max}} = -\frac{m}{v_0}$$

$$\frac{\partial c_s}{\partial v_0} = \frac{a_{max} + g}{{v_0}^2} \cdot m$$

$$\frac{\partial c_s}{\partial m} = -\frac{a_{max} + g}{v_0}$$

Result:

$$\Delta c_s = \left|\frac{m}{v_0}\right| \cdot \Delta a_{max} + \left|\frac{a_{max} + g}{{v_0}^2} \cdot m\right| \cdot \Delta v_0 + \left|\frac{a_{max} + g}{v_0}\right| \cdot \Delta m$$

$$\Delta c_s = \left|\frac{m}{v_0}\right| \cdot \Delta a_{max} + \left|\frac{G_{max}+1}{{v_0}^2}\right| \cdot g \cdot m \cdot \Delta v_0 + \left|\frac{G_{max}+1}{v_0}\right| \cdot g \cdot \Delta m$$

*Formulae 41 and 42*

$$c_h = \left(\frac{a_{max}-g}{v_0} + \frac{2}{t_{P0}}\right) \cdot m$$

$$\Delta c_h = \left|\frac{a_{max}-g}{v_0} + \frac{2}{t_{P0}}\right| \cdot \Delta m + \frac{m}{|v_0|} \cdot \Delta a_{max} + \frac{2 \cdot m}{{t_{P0}}^2} \cdot \Delta t_{P0} + \frac{m \cdot (a_{max}-g)}{{v_0}^2} \cdot \Delta v_0 \quad (41)$$

$$k_h = \left(\frac{a_{max} \cdot (a_{max}-g)}{{v_0}^2} + \frac{2 \cdot (a_{max}-g)}{t_{P0} \cdot v_0} + \frac{2}{{t_{P0}}^2} - \frac{{v_0}^2}{l^2}\right) \cdot m$$

$$\Delta k_h = \frac{2 \cdot m \cdot {v_0}^2}{l^3} \cdot \Delta l + 2 \cdot m \cdot \left|\frac{3 \cdot a_{max} - g}{v_0 \cdot {t_{P0}}^2} - \frac{2}{{t_{P0}}^3}\right| \cdot \Delta t_{P0} + \left|\frac{a_{max} \cdot (a_{max}-g)}{{v_0}^2} + \frac{2}{{t_{P0}}^2} - \frac{{v_0}^2}{l^2} - \frac{2 \cdot (3 \cdot a_{max} - g)}{v_0 \cdot t_{P0}}\right| \cdot \Delta m + \left|\frac{2 \cdot a_{max} - g}{{v_0}^2} - \frac{6}{v_0 \cdot t_{P0}}\right| \cdot \Delta a_{max} + \left|\frac{2 \cdot a_{max} \cdot (a_{max}-g)}{{v_0}^3} + \frac{2 \cdot v_0}{l^2} - \frac{2 \cdot (3 \cdot a_{max} - g)}{t_{P0} \cdot {v_0}^2}\right| \cdot \Delta v_0 \quad (42)$$

Uses (28), (29)

$$x(t) = v_0 \cdot t - \frac{g \cdot m + c \cdot v_0}{2 \cdot m} \cdot t^2 + \frac{c^2 \cdot l^2 \cdot v_0 + g \cdot c \cdot l^2 \cdot m - k \cdot l^2 \cdot m \cdot v_0 - m^2 \cdot {v_0}^3}{6 \cdot l^2 \cdot m^2} \cdot t^3 \quad (28)$$

$$p(t) = v_0 \cdot t - \frac{a_{max} \cdot tp_0 + 2 \cdot v_0}{2 \cdot tp_0} \cdot t^2 + \frac{a_{max} \cdot tp_0 + v_0}{3 \cdot {tp_0}^2} \cdot t^3 \quad (29)$$

The comparison of coefficients leads to

$$\frac{g \cdot m + c \cdot v_0}{2 \cdot m} = \frac{a_{max} \cdot t_{P0} + 2 \cdot v_0}{2 \cdot t_{P0}}$$

$$\frac{c^2 \cdot l^2 \cdot v_0 + g \cdot c \cdot l^2 \cdot m - k \cdot l^2 \cdot m \cdot v_0 - m^2 \cdot {v_0}^3}{6 \cdot l^2 \cdot m^2} = \frac{a_{max} \cdot t_{P0} + v_0}{3 \cdot {t_{P0}}^2} \quad (40)$$

Calculation of $c_h$

$$\frac{g \cdot m + c_h \cdot v_0}{m} = \frac{a_{max} \cdot t_{P0} + 2 \cdot v_0}{t_{P0}}$$

$$(g \cdot m + c_h \cdot v_0) \cdot t_{P0} = (a_{max} \cdot t_{P0} + 2 \cdot v_0) \cdot m$$

$$g \cdot m \cdot t_{P0} + c_h \cdot v_0 \cdot t_{P0} = a_{max} \cdot t_{P0} \cdot m + 2 \cdot v_0 \cdot m$$

$$c_h \cdot v_0 \cdot t_{P0} = a_{max} \cdot t_{P0} \cdot m + 2 \cdot v_0 \cdot m - g \cdot m \cdot t_{P0}$$

$$c_h = \frac{a_{max} \cdot t_{P0} \cdot m + 2 \cdot v_0 \cdot m - g \cdot m \cdot t_{P0}}{v_0 \cdot t_{P0}} = \frac{a_{max} \cdot t_{P0} - g \cdot t_{P0} + 2 \cdot v_0}{v_0 \cdot t_{P0}} \cdot m = \frac{(a_{max} - g) \cdot t_{P0} + 2 \cdot v_0}{v_0 \cdot t_{P0}} \cdot m$$

$$c_h = \left(\frac{a_{max} - g}{v_0} + \frac{2}{t_{P0}}\right) \cdot m$$

$$c_h = \left(\frac{G_{max} - 1}{v_0} \cdot g + \frac{2}{t_{P0}}\right) \cdot m$$

Calculation of $k_h$

Using

$$\frac{{c_h}^2 \cdot l^2 \cdot v_0 + g \cdot c_h \cdot l^2 \cdot m - k \cdot l^2 \cdot m \cdot v_0 - m^2 \cdot {v_0}^3}{6 \cdot l^2 \cdot m^2} = \frac{a_{max} \cdot t_{P0} + v_0}{3 \cdot {t_{P0}}^2}$$

$$c_h = \left(\frac{a_{max} - g}{v_0} + \frac{2}{t_{P0}}\right) \cdot m = \frac{a_{max} \cdot t_{P0} - g \cdot t_{P0} + 2 \cdot v_0}{v_0 \cdot t_{P0}} \cdot m$$

leads to

$$c_h = \frac{a_{max} \cdot t_{P0} - g \cdot t_{P0} + 2 \cdot v_0}{v_0 \cdot t_{P0}} \cdot m$$

$$({c_h}^2 \cdot l^2 \cdot v_0 + g \cdot c_h \cdot l^2 \cdot m - k_h \cdot l^2 \cdot m \cdot v_0 - m^2 \cdot {v_0}^3) \cdot {t_{P0}}^2 = (a_{max} \cdot t_{P0} + v_0) \cdot 2 \cdot l^2 \cdot m^2$$

$$c_h \cdot v_0 \cdot t_{P0} = (a_{max} \cdot t_{P0} - g \cdot t_{P0} + 2 \cdot v_0) \cdot m$$

$${c_h}^2 \cdot l^2 \cdot v_0 \cdot {t_{P0}}^2 + g \cdot c_h \cdot l^2 \cdot m \cdot {t_{P0}}^2 - k_h \cdot l^2 \cdot m \cdot v_0 \cdot {t_{P0}}^2 - m^2 \cdot {v_0}^3 \cdot {t_{P0}}^2 = a_{max} \cdot t_{P0} \cdot 2 \cdot l^2 \cdot m^2 + v_0 \cdot 2 \cdot l^2 \cdot m^2$$

$${c_h}^2 \cdot {v_0}^2 \cdot {t_{P0}}^2 = (a_{max} \cdot t_{P0} - g \cdot t_{P0} + 2 \cdot v_0)^2 \cdot m^2$$

$${c_h}^2 \cdot l^2 \cdot {v_0}^2 \cdot {t_{P0}}^2 + g \cdot c_h \cdot l^2 \cdot m \cdot {t_{P0}}^2 \cdot v_0 - k_h \cdot l^2 \cdot m \cdot {v_0}^2 \cdot {t_{P0}}^2 - m^2 \cdot {v_0}^4 \cdot {t_{P0}}^2 = a_{max} \cdot t_{P0} \cdot 2 \cdot l^2 \cdot m^2 \cdot v_0 + {v_0}^2 \cdot 2 \cdot l^2 \cdot m^2$$

$$l^2 \cdot (a_{max} \cdot t_{P0} - g \cdot t_{P0} + 2 \cdot v_0)^2 \cdot m^2 + g \cdot l^2 \cdot m \cdot t_{P0} \cdot (a_{max} \cdot t_{P0} - g \cdot t_{P0} + 2 \cdot v_0) \cdot m - k_h \cdot l^2 \cdot m \cdot {v_0}^2 \cdot {t_{P0}}^2 - m^2 \cdot {v_0}^4 \cdot {t_{P0}}^2 = a_{max} \cdot t_{P0} \cdot 2 \cdot l^2 \cdot m^2 \cdot v_0 + {v_0}^2 \cdot 2 \cdot l^2 \cdot m^2$$

$$l^2 \cdot (a_{max} \cdot t_{P0} - g \cdot t_{P0} + 2 \cdot v_0)^2 \cdot m^2 + (g \cdot l^2 \cdot m^2 \cdot {t_{P0}}^2 \cdot a_{max} - g^2 \cdot l^2 \cdot m^2 \cdot {t_{P0}}^2 + g \cdot l^2 \cdot m^2 \cdot t_{P0} \cdot 2 \cdot v_0) - a_{max} \cdot t_{P0} \cdot 2 \cdot l^2 \cdot m^2 \cdot v_0 - {v_0}^2 \cdot 2 \cdot l^2 \cdot m^2 - m^2 \cdot {v_0}^4 \cdot {t_{P0}}^2 = k_h \cdot l^2 \cdot m \cdot {v_0}^2 \cdot {t_{P0}}^2$$

$$l^2 \cdot (a_{max} \cdot t_{P0} - g \cdot t_{P0} + 2 \cdot v_0)^2 \cdot m^2 + g \cdot l^2 \cdot m^2 \cdot {t_{P0}}^2 \cdot a_{max} - g^2 \cdot l^2 \cdot m^2 \cdot {t_{P0}}^2 + g \cdot l^2 \cdot m^2 \cdot t_{P0} \cdot 2 \cdot v_0 - a_{max} \cdot t_{P0} \cdot 2 \cdot l^2 \cdot m^2 \cdot v_0 - {v_0}^2 \cdot 2 \cdot l^2 \cdot m^2 - m^2 \cdot {v_0}^4 \cdot {t_{P0}}^2 = k_h \cdot l^2 \cdot m \cdot {v_0}^2 \cdot {t_{P0}}^2$$

$$k_h \cdot l^2 \cdot m \cdot {v_0}^2 \cdot {t_{P0}}^2 = l^2 \cdot (a_{max} \cdot t_{P0} - g \cdot t_{P0} + 2 \cdot v_0)^2 \cdot m^2 + g \cdot l^2 \cdot m^2 \cdot {t_{P0}}^2 \cdot a_{max} - g^2 \cdot l^2 \cdot m^2 \cdot {t_{P0}}^2 + g \cdot l^2 \cdot m^2 \cdot t_{P0} \cdot 2 \cdot v_0 - a_{max} \cdot t_{P0} \cdot 2 \cdot l^2 \cdot m^2 \cdot v_0 - {v_0}^2 \cdot 2 \cdot l^2 \cdot m^2 - m^2 \cdot {v_0}^4 \cdot {t_{P0}}^2$$

$$k_h \cdot l^2 \cdot m \cdot {v_0}^2 \cdot {t_{P0}}^2 = l^2 \cdot m^2 \cdot ({a_{max}}^2 \cdot {t_{P0}}^2 + g^2 \cdot {t_{P0}}^2 + 4 \cdot {v_0}^2 - 2 \cdot a_{max} \cdot {t_{P0}}^2 \cdot g - 4 \cdot g \cdot t_{P0} \cdot v_0 + 4 \cdot a_{max} \cdot t_{P0} \cdot v_0) + g \cdot l^2 \cdot m^2 \cdot {t_{P0}}^2 \cdot a_{max} - g^2 \cdot l^2 \cdot m^2 \cdot {t_{P0}}^2 + g \cdot l^2 \cdot m^2 \cdot t_{P0} \cdot 2 \cdot v_0 - a_{max} \cdot t_{P0} \cdot 2 \cdot l^2 \cdot m^2 \cdot v_0 - {v_0}^2 \cdot 2 \cdot l^2 \cdot m^2 - m^2 \cdot {v_0}^4 \cdot {t_{P0}}^2$$

$$k_h \cdot l^2 \cdot m \cdot {v_0}^2 \cdot {t_{P0}}^2 = (l^2 \cdot m^2 \cdot {a_{max}}^2 \cdot {t_{P0}}^2 + l^2 \cdot m^2 \cdot g^2 \cdot {t_{P0}}^2 + l^2 \cdot m^2 \cdot 4 \cdot {v_0}^2 - l^2 \cdot m^2 \cdot 2 \cdot a_{max} \cdot {t_{P0}}^2 \cdot g - l^2 \cdot m^2 \cdot 4 \cdot g \cdot t_{P0} \cdot v_0 + l^2 \cdot m^2 \cdot 4 \cdot a_{max} \cdot t_{P0} \cdot v_0) + g \cdot l^2 \cdot m^2 \cdot {t_{P0}}^2 \cdot a_{max} - g^2 \cdot l^2 \cdot m^2 \cdot {t_{P0}}^2 + g \cdot l^2 \cdot m^2 \cdot t_{P0} \cdot 2 \cdot v_0 - a_{max} \cdot t_{P0} \cdot 2 \cdot l^2 \cdot m^2 \cdot v_0 - {v_0}^2 \cdot 2 \cdot l^2 \cdot m^2 - m^2 \cdot {v_0}^4 \cdot {t_{P0}}^2$$

$$k_h \cdot l^2 \cdot m \cdot {v_0}^2 \cdot {t_{P0}}^2 = l^2 \cdot m^2 \cdot {a_{max}}^2 \cdot {t_{P0}}^2 + l^2 \cdot m^2 \cdot g^2 \cdot {t_{P0}}^2 + l^2 \cdot m^2 \cdot 4 \cdot {v_0}^2 - l^2 \cdot m^2 \cdot 2 \cdot a_{max} \cdot {t_{P0}}^2 \cdot g - l^2 \cdot m^2 \cdot 4 \cdot g \cdot t_{P0} \cdot v_0 + l^2 \cdot m^2 \cdot 4 \cdot a_{max} \cdot t_{P0} \cdot v_0 + g \cdot l^2 \cdot m^2 \cdot {t_{P0}}^2 \cdot a_{max} - g^2 \cdot l^2 \cdot m^2 \cdot {t_{P0}}^2 + g \cdot l^2 \cdot m^2 \cdot t_{P0} \cdot 2 \cdot v_0 - a_{max} \cdot t_{P0} \cdot 2 \cdot l^2 \cdot m^2 \cdot v_0 - {v_0}^2 \cdot 2 \cdot l^2 \cdot m^2 - m^2 \cdot {v_0}^4 \cdot {t_{P0}}^2$$

$$k_h \cdot l^2 \cdot m \cdot {v_0}^2 \cdot {t_{P0}}^2 = l^2 \cdot m^2 \cdot {a_{max}}^2 \cdot {t_{P0}}^2 + l^2 \cdot m^2 \cdot g^2 \cdot {t_{P0}}^2 + l^2 \cdot m^2 \cdot 4 \cdot {v_0}^2 - l^2 \cdot m^2 \cdot 2 \cdot a_{max} \cdot {t_{P0}}^2 \cdot g - l^2 \cdot m^2 \cdot 4 \cdot g \cdot t_{P0} \cdot v_0 + l^2 \cdot m^2 \cdot 4 \cdot a_{max} \cdot t_{P0} \cdot v_0 + g \cdot l^2 \cdot m^2 \cdot {t_{P0}}^2 \cdot a_{max} - g^2 \cdot l^2 \cdot m^2 \cdot {t_{P0}}^2 + g \cdot l^2 \cdot m^2 \cdot t_{P0} \cdot 2 \cdot v_0 - a_{max} \cdot t_{P0} \cdot 2 \cdot l^2 \cdot m^2 \cdot v_0 - {v_0}^2 \cdot 2 \cdot l^2 \cdot m^2 - m^2 \cdot {v_0}^4 \cdot {t_{P0}}^2$$

$$k_h \cdot l^2 \cdot m \cdot {v_0}^2 \cdot {t_{P0}}^2 = l^2 \cdot m^2 \cdot {a_{max}}^2 \cdot {t_{P0}}^2 + l^2 \cdot m^2 \cdot 2 \cdot {v_0}^2 - l^2 \cdot m^2 \cdot 2 \cdot g \cdot t_{P0} \cdot v_0 + l^2 \cdot m^2 \cdot 2 \cdot a_{max} \cdot t_{P0} \cdot v_0 - g \cdot l^2 \cdot m^2 \cdot {t_{P0}}^2 \cdot a_{max} - m^2 \cdot {v_0}^4 \cdot {t_{P0}}^2$$

$$k_h \cdot l^2 \cdot {v_0}^2 \cdot {t_{P0}}^2 = l^2 \cdot m \cdot {a_{max}}^2 \cdot {t_{P0}}^2 + l^2 \cdot m \cdot 2 \cdot {v_0}^2 - l^2 \cdot m \cdot 2 \cdot g \cdot t_{P0} \cdot v_0 + l^2 \cdot m \cdot 2 \cdot a_{max} \cdot t_{P0} \cdot v_0 - g \cdot l^2 \cdot m \cdot {t_{P0}}^2 \cdot a_{max} - m \cdot {v_0}^4 \cdot {t_{P0}}^2$$

$$k_h \cdot l^2 \cdot {v_0}^2 \cdot {t_{P0}}^2 = l^2 \cdot m \cdot {a_{max}}^2 \cdot {t_{P0}}^2 - g \cdot l^2 \cdot m \cdot {t_{P0}}^2 \cdot a_{max} + l^2 \cdot m \cdot 2 \cdot a_{max} \cdot t_{P0} \cdot v_0 - l^2 \cdot m \cdot 2 \cdot g \cdot t_{P0} \cdot v_0 + l^2 \cdot m \cdot 2 \cdot {v_0}^2 - m \cdot {v_0}^4 \cdot {t_{P0}}^2$$

$$k_h \cdot l^2 \cdot {v_0}^2 \cdot {t_{P0}}^2 = l^2 \cdot m \cdot {t_{P0}}^2 \cdot a_{max} \cdot a_{max} - l^2 \cdot m \cdot {t_{P0}}^2 \cdot a_{max} \cdot g + l^2 \cdot m \cdot 2 \cdot a_{max} \cdot t_{P0} \cdot v_0 - l^2 \cdot m \cdot 2 \cdot g \cdot t_{P0} \cdot v_0 + l^2 \cdot m \cdot 2 \cdot {v_0}^2 - m \cdot {v_0}^2 \cdot {v_0}^2 \cdot {t_{P0}}^2$$

$$k_h \cdot l^2 \cdot {v_0}^2 \cdot {t_{P0}}^2 = l^2 \cdot m \cdot {t_{P0}}^2 \cdot a_{max} \cdot (a_{max} - g) + l^2 \cdot m \cdot 2 \cdot t_{P0} \cdot v_0 \cdot (a_{max} - g) + m \cdot {v_0}^2 \cdot (2 \cdot l^2 - {v_0}^2 \cdot {t_{P0}}^2)$$

$$k_h = \frac{l^2 \cdot m \cdot {t_{P0}}^2 \cdot a_{max} \cdot (a_{max} - g)}{l^2 \cdot {v_0}^2 \cdot {t_{P0}}^2} + \frac{l^2 \cdot m \cdot 2 \cdot t_{P0} \cdot v_0 \cdot (a_{max} - g)}{l^2 \cdot {v_0}^2 \cdot {t_{P0}}^2} + \frac{m \cdot {v_0}^2 \cdot (2 \cdot l^2 - {v_0}^2 \cdot {t_{P0}}^2)}{l^2 \cdot {v_0}^2 \cdot {t_{P0}}^2}$$

$$k_h = \left(\frac{a_{max} \cdot (a_{max} - g)}{{v_0}^2} + \frac{2 \cdot (a_{max} - g)}{t_{P0} \cdot v_0} + \frac{2 \cdot l^2 - {v_0}^2 \cdot {t_{P0}}^2}{l^2 \cdot {t_{P0}}^2}\right) \cdot m$$

Result:

$$k_h = \left(\frac{a_{max} \cdot (a_{max} - g)}{{v_0}^2} + \frac{2 \cdot (a_{max} - g)}{t_{P0} \cdot v_0} + \frac{2}{{t_{P0}}^2} - \frac{{v_0}^2}{l^2}\right) \cdot m$$

$$k_h = \left(\frac{G_{max} \cdot g^2 \cdot (G_{max} - 1)}{{v_0}^2} + \frac{2 \cdot g \cdot (G_{max} - 1)}{t_{P0} \cdot v_0} + \frac{2}{{t_{P0}}^2} - \frac{{v_0}^2}{l^2}\right) \cdot m$$

Derivation of the error estimates – $c_h$

$$c_h = \left(\frac{a_{max} - g}{v_0} + \frac{2}{t_{P0}}\right) \cdot m$$

$$\frac{\partial c_h}{\partial a_{max}} = \frac{m}{v_0}$$

$$\frac{\partial c_h}{\partial v_0} = -\frac{a_{max} - g}{{v_0}^2} \cdot m$$

$$\frac{\partial c_h}{\partial t_{P0}} = -\frac{2 \cdot m}{{t_{P0}}^2}$$

$$\frac{\partial c_h}{\partial m} = \frac{a_{max} - g}{v_0} + \frac{2}{t_{P0}}$$

$$\Delta c_h = \left|\frac{a_{max} - g}{v_0} + \frac{2}{t_{P0}}\right| \cdot \Delta m + \frac{m}{|v_0|} \cdot \Delta a_{max} + \frac{2 \cdot m}{{t_{P0}}^2} \cdot \Delta t_{P0} + \frac{m \cdot (a_{max} - g)}{{v_0}^2} \cdot \Delta v_0$$

$$\Delta c_h = \left|\frac{G_{max} - 1}{v_0} \cdot g + \frac{2}{t_{P0}}\right| \cdot \Delta m + \frac{m}{|v_0|} \cdot g \cdot \Delta G_{max} + \frac{2 \cdot m}{{t_{P0}}^2} \cdot \Delta t_{P0} + \frac{m \cdot (G_{max} - 1)}{{v_0}^2} \cdot g \cdot \Delta v_0$$

Derivation of the error estimates – $k_h$

$$k_h = \left(\frac{a_{max} \cdot (a_{max} - g)}{{v_0}^2} + \frac{2 \cdot (a_{max} - g)}{t_{P0} \cdot v_0} + \frac{2}{{t_{P0}}^2} - \frac{{v_0}^2}{l^2}\right) \cdot m$$

$$\frac{\partial k_h}{\partial a_{max}} = \left(\frac{2 \cdot a_{max} - g}{{v_0}^2} + \frac{2}{t_{P0} \cdot v_0}\right) \cdot m$$

$$\frac{\partial k_h}{\partial v_0} = \left(-2 \cdot \frac{a_{max} \cdot (a_{max} - g)}{{v_0}^3} - \frac{2 \cdot (a_{max} - g)}{t_{P0} \cdot {v_0}^2} - \frac{2 \cdot v_0}{l^2}\right) \cdot m$$

$$\frac{\partial k_h}{\partial t_{P0}} = \left(-\frac{2 \cdot (a_{max} - g)}{{t_{P0}}^2 \cdot v_0} - \frac{4}{{t_{P0}}^3}\right) \cdot m$$

$$\frac{\partial k_h}{\partial m} = \frac{a_{max} \cdot (a_{max} - g)}{{v_0}^2} + \frac{2 \cdot (a_{max} - g)}{t_{P0} \cdot v_0} + \frac{2}{{t_{P0}}^2} - \frac{{v_0}^2}{l^2}$$

$$\frac{\partial k_h}{\partial l} = 2 \cdot \frac{{v_0}^2}{l^3} \cdot m$$

Result:

$$\Delta k_h = \left|\frac{a_{max} \cdot (a_{max} - g)}{{v_0}^2} + \frac{2 \cdot (a_{max} - g)}{t_{P0} \cdot v_0} + \frac{2}{{t_{P0}}^2} - \frac{{v_0}^2}{l^2}\right| \cdot \Delta m + m \cdot \left|\frac{2 \cdot a_{max} - g}{{v_0}^2} + \frac{2}{t_{P0} \cdot v_0}\right| \cdot \Delta a_{max} + \frac{2 \cdot m}{{t_{P0}}^2}$$
$$\cdot \left|\frac{a_{max} - g}{v_0} + \frac{2}{t_{P0}}\right| \cdot \Delta t_{P0} + 2 \cdot m \cdot \left|\frac{a_{max} \cdot (a_{max} - g)}{{v_0}^3} + \frac{a_{max} - g}{t_{P0} \cdot {v_0}^2} + \frac{v_0}{l^2}\right| \cdot \Delta v_0 + \frac{2 \cdot {v_0}^2 \cdot m}{l^3} \cdot \Delta l$$

$$\Delta k_h = \left|\frac{G_{max} \cdot g^2 \cdot (G_{max} - 1)}{{v_0}^2} + \frac{2 \cdot g \cdot (G_{max} - 1)}{t_{P0} \cdot v_0} + \frac{2}{{t_{P0}}^2} - \frac{{v_0}^2}{l^2}\right| \cdot \Delta m + m \cdot \left|\frac{2 \cdot G_{max} - 1}{{v_0}^2} \cdot g + \frac{2}{t_{P0} \cdot v_0}\right| \cdot g \cdot \Delta G_{max} + \frac{2 \cdot m}{{t_{P0}}^2}$$
$$\cdot \left|\frac{G_{max} - 1}{v_0} \cdot g + \frac{2}{t_{P0}}\right| \cdot \Delta t_{P0} + 2 \cdot m \cdot \left|\frac{G_{max} \cdot g^2 \cdot (G_{max} - 1)}{{v_0}^3} + \frac{G_{max} - 1}{t_{P0} \cdot {v_0}^2} \cdot g + \frac{v_0}{l^2}\right| \cdot \Delta v_0 + \frac{2 \cdot {v_0}^2 \cdot m}{l^3} \cdot \Delta l$$

# Chapter 2.3.2

*Formulae 43*

$$K = \frac{{G_{max}}^2 \cdot m}{{T_0}^2}$$
$$\frac{\Delta K}{K} = 2 \cdot \frac{\Delta G_{max}}{G_{max}} + 2 \cdot \frac{\Delta T_0}{T_0} + \frac{\Delta m}{m} \quad (43)$$

Derivation of the error estimates

$$\frac{\partial K}{\partial G_{max}} = 2 \cdot \frac{G_{max} \cdot m}{{T_0}^2}$$
$$\frac{\partial K}{\partial T_0} = \frac{\partial}{\partial T_0}\left(\frac{{G_{max}}^2 \cdot m}{{T_0}^2}\right) = -2 \cdot \frac{{G_{max}}^2 \cdot m}{{T_0}^3}$$
$$\frac{\partial K}{\partial m} = \frac{{G_{max}}^2}{{T_0}^2}$$

$$\Delta K = 2 \cdot \frac{G_{max} \cdot m}{{T_0}^2} \cdot \Delta G_{max} + 2 \cdot \frac{{G_{max}}^2 \cdot m}{{T_0}^3} \cdot \Delta T_0 + \frac{{G_{max}}^2}{{T_0}^2} \cdot \Delta m =$$
$$= 2 \cdot \frac{{G_{max}}^2 \cdot m}{{T_0}^2} \cdot \frac{\Delta G_{max}}{G_{max}} + 2 \cdot \frac{{G_{max}}^2 \cdot m}{{T_0}^2} \cdot \frac{\Delta T_0}{T_0} + \frac{{G_{max}}^2 \cdot m}{{T_0}^2} \cdot \frac{\Delta m}{m} = 2 \cdot K \cdot \frac{\Delta G_{max}}{G_{max}} + 2 \cdot K \cdot \frac{\Delta T_0}{T_0} + K \cdot \frac{\Delta m}{m}$$

Result:

$$\frac{\Delta K}{K} = 2 \cdot \frac{\Delta G_{max}}{G_{max}} + 2 \cdot \frac{\Delta T_0}{T_0} + \frac{\Delta m}{m}$$

*Formulae 44*

$$E_R = \left(\frac{T_1}{T_0}\right)^2$$
$$\frac{\Delta E_R}{E_R} = 2 \cdot \left(\frac{\Delta T_0}{T_0} + \frac{\Delta T_1}{T_1}\right) \quad (44)$$

Derivation of the error estimates

$$\frac{\partial E_R}{\partial T_0} = \frac{\partial}{\partial T_0}\left({T_1}^2 \cdot {T_0}^{-2}\right) = -2 \cdot {T_1}^2 \cdot {T_0}^{-3} = -2 \cdot \left(\frac{T_1}{T_0}\right)^2 \cdot \frac{1}{T_0}$$
$$\frac{\partial E_R}{\partial T_1} = \frac{\partial}{\partial T_1}\left({T_1}^2 \cdot {T_0}^{-2}\right) = 2 \cdot T_1 \cdot {T_0}^{-2} = 2 \cdot \left(\frac{T_1}{T_0}\right)^2 \cdot \frac{1}{T_1}$$

$$\Delta E_R = \left|-2 \cdot \left(\frac{T_1}{T_0}\right)^2 \cdot \frac{1}{T_0}\right| \cdot \Delta T_0 + \left|2 \cdot \left(\frac{T_1}{T_0}\right)^2 \cdot \frac{1}{T_1}\right| \cdot \Delta T_1 = 2 \cdot E_R \cdot \frac{1}{T_0} \cdot \Delta T_0 + 2 \cdot E_R \cdot \frac{1}{T_1} \cdot \Delta T_1$$

Result:

$$\frac{\Delta E_R}{E_R} = 2 \cdot \frac{\Delta T_0}{T_0} + 2 \cdot \frac{\Delta T_1}{T_1} = 2 \cdot \left(\frac{\Delta T_0}{T_0} + \frac{\Delta T_1}{T_1}\right)$$

*Formulae 45*

$$f_n = \frac{1}{2 \cdot d_0}$$

$$\frac{\Delta f_n}{f_n} = \frac{\Delta d_0}{d_0} \tag{45}$$

Derivation of the error estimates

$$\frac{\partial f_n}{\partial d_0} = \frac{1}{2} \cdot \frac{\partial}{\partial d_0} {d_0}^{-1} = -\frac{1}{2} \cdot {d_0}^{-2} = -\frac{1}{2 \cdot d_0} \cdot \frac{1}{d_0}$$

$$\Delta f_n = \left| -\frac{1}{2 \cdot d_0} \cdot \frac{1}{d_0} \right| \cdot \Delta d_0 = \frac{1}{2 \cdot d_0} \cdot \frac{\Delta d_0}{d_0} = f_n \cdot \frac{\Delta d_0}{d_0}$$

Result:

$$\frac{\Delta f_n}{f_n} = \frac{\Delta d_0}{d_0}$$

*Formulae 46*

$$E^* = \frac{3}{4} \cdot \frac{m \cdot g \cdot G_{max}}{\sqrt{R} \cdot \sqrt{D^3}}$$

$$\frac{\Delta E^*}{E^*} = \frac{\Delta m}{m} + \frac{\Delta G_{max}}{G_{max}} + \frac{1}{2} \cdot \frac{\Delta R}{R} + \frac{3}{2} \cdot \frac{\Delta D}{D} \tag{46}$$

Derivation of the error estimates

$$\frac{\partial E^*}{\partial m} = \frac{3}{4} \cdot \frac{g \cdot G_{max}}{\sqrt{R} \cdot \sqrt{D^3}}$$

$$\frac{\partial E^*}{\partial G_{max}} = \frac{3}{4} \cdot \frac{m \cdot g}{\sqrt{R} \cdot \sqrt{D^3}}$$

$$\frac{\partial E^*}{\partial R} = \frac{3}{4} \cdot \frac{m \cdot g \cdot G_{max}}{\sqrt{D^3}} \cdot \frac{\partial}{\partial R}\left(R^{-\frac{1}{2}}\right) = \frac{3}{4} \cdot \frac{m \cdot g \cdot G_{max}}{\sqrt{D^3}} \cdot \left(-\frac{1}{2}\right) \cdot R^{-\frac{3}{2}} = -\frac{1}{2} \cdot \left(\frac{3}{4} \cdot \frac{m \cdot g \cdot G_{max}}{\sqrt{D^3}} \cdot R^{-\frac{1}{2}}\right) \cdot R^{-1}$$

$$\frac{\partial E^*}{\partial D} = \frac{3}{4} \cdot \frac{m \cdot g \cdot G_{max}}{\sqrt{R}} \cdot \frac{\partial}{\partial D}\left(D^{-\frac{3}{2}}\right) = \frac{3}{4} \cdot \frac{m \cdot g \cdot G_{max}}{\sqrt{R}} \cdot \left(-\frac{3}{2}\right) \cdot D^{-\frac{5}{2}} = -\frac{3}{2} \cdot \left(\frac{3}{4} \cdot \frac{m \cdot g \cdot G_{max}}{\sqrt{R}} \cdot D^{-\frac{3}{2}}\right) \cdot D^{-1}$$

$$\Delta E^* = \frac{3}{4} \cdot \frac{m \cdot g \cdot G_{max}}{\sqrt{R} \cdot \sqrt{D^3}} \cdot \frac{1}{m} \cdot \Delta m + \frac{3}{4} \cdot \frac{m \cdot g}{\sqrt{R} \cdot \sqrt{D^3}} \cdot \Delta G_{max} + \frac{1}{2} \cdot \left(\frac{3}{4} \cdot \frac{m \cdot g \cdot G_{max}}{\sqrt{D^3}} \cdot R^{-\frac{1}{2}}\right) \cdot \frac{1}{R} \cdot \Delta R + \frac{3}{2} \cdot \left(\frac{3}{4} \cdot \frac{m \cdot g \cdot G_{max}}{\sqrt{R}} \cdot D^{-\frac{3}{2}}\right) \cdot \frac{1}{D}$$

$$\cdot \Delta D = E^* \cdot \frac{\Delta m}{m} + E^* \cdot \frac{\Delta G_{max}}{G_{max}} + \frac{1}{2} \cdot E^* \cdot \frac{\Delta R}{R} + \frac{3}{2} \cdot E^* \cdot \frac{\Delta D}{D} =$$

Result:

$$\frac{\Delta E^*}{E^*} = \frac{\Delta m}{m} + \frac{\Delta G_{max}}{G_{max}} + \frac{1}{2} \cdot \frac{\Delta R}{R} + \frac{3}{2} \cdot \frac{\Delta D}{D}$$

*Formulae 47*

$$T_{0,free} : T_{0,hammer} : T_{0,average} = 1 : \sqrt{\frac{l^2}{l^2 - h^2}} : \sqrt{\frac{2 \cdot l^2}{2 \cdot l^2 - h^2}} \tag{47}$$

Derivation and result

$$T_{0,free} : T_{0,hammer} : T_{0,average} = \left(\sqrt{\frac{2 \cdot h}{g}}\right) : \left(\sqrt{\frac{l^2}{l^2 - h^2}} \cdot \sqrt{\frac{2 \cdot h}{g}}\right) : \left(\sqrt{\frac{2 \cdot l^2}{2 \cdot l^2 - h^2}} \cdot \sqrt{\frac{2 \cdot h}{g}}\right) = 1 : \sqrt{\frac{l^2}{l^2 - h^2}} : \sqrt{\frac{2 \cdot l^2}{2 \cdot l^2 - h^2}}$$

*Formulae 48*

$$E_{r,free} : E_{r,hammer} : E_{r,average} = 1 : \frac{l^2 - h^2}{l^2} : \frac{2 \cdot l^2 - h^2}{2 \cdot l^2} \tag{48}$$

Derivation and result

$$E_{R,free} : E_{R,hammer} : E_{R,average} = \left(\frac{T_1}{T_{0,free}}\right)^2 : \left(\frac{T_1}{T_{0,hammer}}\right)^2 : \left(\frac{T_1}{T_{0,average}}\right)^2 = \frac{1}{{T_{0,free}}^2} : \frac{1}{{T_{0,hammer}}^2} : \frac{1}{{T_{0,average}}^2} =$$

$$= \left(\frac{g}{2 \cdot h}\right) : \left(\frac{l^2 - h^2}{l^2} \cdot \frac{g}{2 \cdot h}\right) : \left(\frac{2 \cdot l^2 - h^2}{2 \cdot l^2} \cdot \frac{g}{2 \cdot h}\right) = 1 : \frac{l^2 - h^2}{l^2} : \frac{2 \cdot l^2 - h^2}{2 \cdot l^2}$$

*Formulae 49*

$$k_{free} : k_{hammer} : k_{average} = 1 : \frac{l^2 - h^2}{l^2} : \frac{2 \cdot l^2 - h^2}{2 \cdot l^2} \tag{49}$$

Derivation and result

$$k_{free} : k_{hammer} : k_{average} = \left(\frac{{G_{max}}^2 \cdot m}{{T_{0,free}}^2}\right) : \left(\frac{{G_{max}}^2 \cdot m}{{T_{0,hammer}}^2}\right) : \left(\frac{{G_{max}}^2 \cdot m}{{T_{0,average}}^2}\right) = \frac{1}{{T_{0,free}}^2} : \frac{1}{{T_{0,hammer}}^2} : \frac{1}{{T_{0,average}}^2} =$$

$$= \left(\frac{g}{2 \cdot h}\right) : \left(\frac{l^2 - h^2}{l^2} \cdot \frac{g}{2 \cdot h}\right) : \left(\frac{2 \cdot l^2 - h^2}{2 \cdot l^2} \cdot \frac{g}{2 \cdot h}\right) = 1 : \frac{l^2 - h^2}{l^2} : \frac{2 \cdot l^2 - h^2}{2 \cdot l^2}$$
