# Supplementary material for: Mathematical Framework for the Representation of the Travel of an Accelerometer-Based Texture Testing Device
Source: Sensors (Basel). 2025 May 22;25(11):3273. doi: 10.3390/s25113273 (PMC12157044; doi:10.3390/s25113273)
Supplement: Supplementary file 1 [file sensors-25-03273-s001.zip › Methodology_ revised 2025.pdf]

Methodology

The supplementary Figure 2 can be found in the article, Chapter 2.1.1.

Restitution coefficient and energy restitution.

Assume that a mass m drops from height h driven by gravity g=9.81ms^{-2}. Duration of the first free falling period T0 allows to calculate the effective drop height h_0 by

$$h_0 = \frac{g}{2} \cdot T_0^2$$

Impact velocity, $v_0 = g \cdot T_0$ result in a kinetic energy of $= \frac{m \cdot v_0^2}{2}$. Penetration of the ground causes energy dissipation and a reduced velocity v_1 after the first bounce. The quotient v_1/v_0 is known as the coefficient of restitution CR a property of the collision partners. The velocity after the impact is given accordingly by $v_1 = g \cdot T_1$ with $2 \cdot T_1$ as the time period between the first and the second impact A. Another useful parameter is the energy restitution ER that can be calculated as $ER = CR^2$ or directly from time periods $ER = \left(\frac{T_1}{T_0}\right)^2$ In addition $ER = \frac{E_{pot\_1}}{E_{pot\_0}} = \frac{h_1}{h_0}$ is used for drop tests.

Spring rate

The impact of a mass on an infinite hard surface would cause infinite acceleration. In reality this value is determined by the kinetic energy of the mass, its shape and the elastic properties of both items. This can be modelled as a mass impacting on a spring.

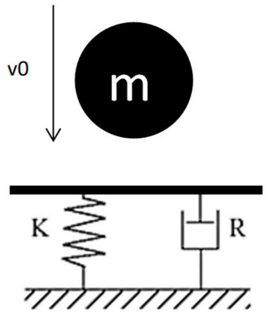

By integration of F= k·x, we get the energy stored in the spring as

$$E = \frac{\mathrm{k}}{2} \cdot x^2 + C.$$

Substituting x = F/k leads to

$$F = m \cdot a = \sqrt{2 \cdot E \cdot \mathrm{k}}$$

and

$$\mathrm{k} = \frac{m^2 \cdot a^2}{2 \cdot E}.$$

The kinetic energy of a mass, free falling after a time t can be written as (v= g·t)

$$E = \frac{m \cdot g^2 \cdot t^2}{2}$$

in previous equation we get

$$\mathrm{k} = m \cdot \frac{a^2}{g^2 \cdot t^2}.$$

And with a = g·G we get

$$\mathrm{k} = \frac{G^2 \cdot m}{t^2}$$

This is the “average” spring rate that links impact energy, mass and peak acceleration.

Force reduction

Maximum acceleration and maximum force Fmax are linked according to Newton´s law F=m·a

Force reduction in % is defined as (2)

$$F_{red} = \left(1 - \frac{F_{max}}{F_{ref}}\right) \cdot 100$$

with F_{ref} as the evoked force when the mass hits an infinite hard surface.

According to the mass / spring model with F=k·x, F can be replaced by k accordingly

$$F_{red} = \left(\frac{k_{ref}}{k + k_{ref}}\right) \cdot 100$$

The spring rate k_{ref} should be selected appropriately according to the task and materials involved. In case of equestrian surfaces it may be useful to determine k_{ref} from the force /distance relation of representative hoofs by a material testing machine. Preliminary measurements revealed a value for k of 1000N/mm.

Penetration depth

Double integration of the acceleration over time during ground contact gives the penetration depth. However, a(t) can be considered sinusoidal as a reasonable approximation. Under the assumption of the latter and the known starting velocity v_0 derived from the drop time the penetration depth is approximated by

Johannes Peter Schramel, amended by Harald Paulsen

$$x = \frac{2}{\pi} \cdot v_0 \cdot t_{P0}$$

With t_{P0} as the time between ground contact and peak acceleration the factor $\frac{2}{\pi}$ is the ratio between the area under the sinusoidal curve and the circumlocutory rectangle.

Replacing v_0 by $g \cdot T_0$ lead to

$$x = g \cdot t_{P0} \cdot (T_0 - \frac{2 \cdot G \cdot t_{P0}}{\pi^2})$$

In case a(t) is considered triangular one get

$$x = g \cdot t_{P0} \cdot (T_0 - \frac{G \cdot t_{P0}}{6})$$

Remark: 1/6 =0.166; $2/\pi^2$= 0.2025

Young's modulus

The contact between the spherical mass m and the elastic sample can be described by Hertz's contact theory (3). The normal force occurring during contact is

$$F = \frac{4}{3} \cdot E^* \cdot \sqrt{R} \cdot \sqrt{d^3}$$

as a function of the modulus of elasticity E*, the radius R of the mass m and the penetration depth d.

In comparison with

$$F = m \cdot g \cdot G_{max}$$

results in the following for the Young's modulus

$$E^* = \frac{3}{4} \cdot \frac{m \cdot g \cdot G_{max}}{\sqrt{R} \cdot \sqrt{d^3}}$$

References

(1)Falcon, E., Laroche, C., Fauve, S., Coste, C. 1998 Behaviour of one inelastic ball bouncing repeatedly off the ground European Physical Journal B 3, 45-57.

(2)FIFA Quality Concept Handbook of Test Methods for Football Turf January 2012 Edition

(3) Popov, V. L. 2010 Kontaktmechanik und Reibung Springer Vieweg Berlin, Heidelberg, 63-64
